# Iodinated PSMA Ligands as XFI Tracers for Targeted Cell Imaging and Characterization of Nanoparticles

**DOI:** 10.3390/ijms252211880

**Published:** 2024-11-05

**Authors:** Svenja Kerpa, Malte Holzapfel, Theresa Staufer, Robert Kuhrwahl, Marina Mutas, Stefan Werner, Verena R. Schulze, Pascal Nakielski, Neus Feliu, Elke Oetjen, Jannis Haak, Florian Ziegler, Rasmus Buchin, Jili Han, Wolfgang J. Parak, Florian Grüner, Wolfgang Maison

**Affiliations:** 1 Department of Chemistry, Institute of Pharmacy, Universität Hamburg, Bundesstrasse 45, 20146 Hamburg, Germany; svenja.kerpa@uni-hamburg.de; 2 Fraunhofer Institute for Applied Polymer Research IAP, Center for Applied Nanotechnology CAN, Universität Hamburg, Bundesstrasse 45, 20146 Hamburg, Germany; malte.holzapfel@iap.fraunhofer.de (M.H.); m.mutas@uke.de (M.M.); verena.schulze@iap.fraunhofer.de (V.R.S.); pascal.nakielski@iap.fraunhofer.de (P.N.); neus.feliu.torres@iap.fraunhofer.de (N.F.); 3 University of Hamburg and Center for Free-Electron Laser Science, Luruper Chaussee 149, 22761 Hamburg, Germany; theresa.staufer@uni-hamburg.de (T.S.); robert.florian.kuhrwahl@cfel.de (R.K.); jannis.haak@cfel.de (J.H.); florian.ziegler@cfel.de (F.Z.); paul.rasmus.buchin@cfel.de (R.B.); 4 Department of Tumor Biology, University Medical Center Hamburg-Eppendorf, Martinistr. 52, 20246 Hamburg, Germany; st.werner@uke.de; 5 Mildred Scheel Cancer Career Center HaTriCS4, University Medical Center Hamburg-Eppendorf, Martinistr. 52, 20246 Hamburg, Germany; 6 Institute of Clinical Pharmacology and Toxicology, University Medical Center Hamburg-Eppendorf, Martinistr. 52, 20246 Hamburg, Germany; e.oetjen@uke.de; 7 Department of Physics, Universität Hamburg and Center for Hybrid Nanostructures (CHyN), Luruper Chaussee 149, 22761 Hamburg, Germany; jhan@physnet.uni-hamburg.de (J.H.); wolfgang.parak@uni-hamburg.de (W.J.P.)

**Keywords:** tumor targeting, PSMA, XFI, prostate cancer, quantum dots

## Abstract

Prostate cancer is the second most commonly diagnosed cancer in men worldwide. Despite this, current diagnostic tools are still not satisfactory, lacking sensitivity for early-stage or single-cell diagnosis. This study describes the development of small-molecule tracers for the well-known tumor marker prostate-specific membrane antigen (PSMA). These tracers contain a urea motif for PSMA-targeting and iodinated aromatic moieties to allow detection via X-ray fluorescence imaging (XFI). Tracers with a triiodobenzoyl moiety allowed the specific targeting and successful imaging of PSMA+ cell lines with XFI. The XFI-measured uptake of 7.88 × 10^−18^ mol iodine (I) per cell is consistent with the uptake of known PSMA tracers measured by other techniques such as inductively coupled plasma mass spectrometry (ICP-MS). This is the first successful application of XFI to tumor cell targeting with a small-molecule tracer. In addition, iodinated tracers were used for the characterization of quantum dots (QDs) conjugated to PSMA-targeting urea motifs. The resulting targeted QD conjugates were shown to selectively bind PSMA+ cell lines via confocal microscopy. The immobilized iodinated targeting vectors allowed the determination of the tracer/QD ratio via XFI and ICP-MS. This ratio is a key property of targeted particles and difficult to measure by other techniques.

## 1. Introduction

**Figure 1:**
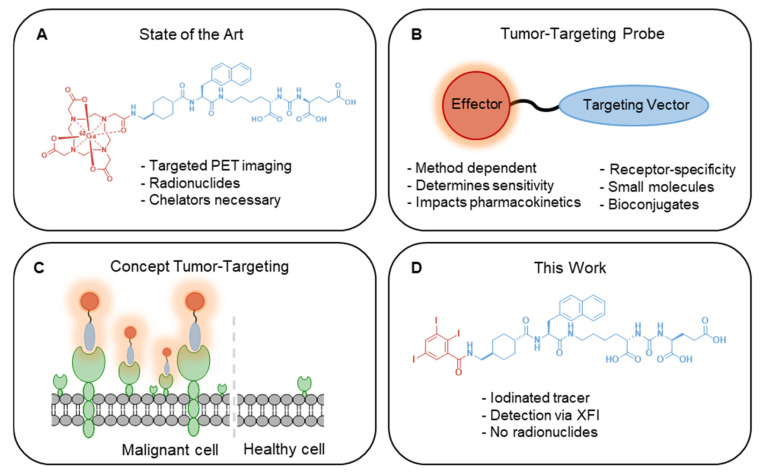
(**A**) Structure of ^68^Ga-PSMA 617 as molecular probe for targeted PET imaging of prostate cancer. (**B**) Schematic drawing of typical molecular probe for tumor targeting. (**C**) Concept of tumor targeting with bifunctional probes. (**D**) Structure of nonradioactive iodinated PSMA-targeted probes for XFI.

Imaging techniques with a high sensitivity and resolution are essential for the early clinical diagnosis of cancer because they allow the non-invasive examination of tissue. Clinical methods differ considerably in terms of cost, sensitivity and patient safety [1]. Techniques like computed tomography (CT) or magnetic resonance imaging (MRI) are often used to image the morphology of tissue [2,3]. Methods with a high sensitivity such as positron emission tomography (PET, often combined with CT) [4,5] single-photon emission computed tomography (SPECT) [6,7] and near-infrared fluorescence (NIR) can be used for the targeted imaging of disease [8,9]. These methods have excellent sensitivity (10^−12^–10^−15^ M) [10] and in some cases allow the elucidation of processes on a cellular level and hence the earliest possible detection of malignant cells. A good example is the use of radiolabeled targeting vectors for the prostate-specific membrane antigen (PSMA) [11,12,13,14]. ^68^Ga-PSMA-617 (Figure 1A) and ^177^Lu-PSMA-617, for example, have been applied successfully to the clinical diagnosis and treatment of prostate cancer via PET [15,16,17,18,19]. The approach combines the favorable pharmacokinetic properties of small molecular drugs with PSMA as an excellent cell surface tumor marker [20,21,22].

However, each of the highly sensitive imaging techniques mentioned above has drawbacks. SPECT offers a reasonably high sensitivity for labeling and investigating biochemical processes, but has a limited resolution and is cost-intensive [23]. PET/CT provides high-resolution images and enables the quantification of biochemical changes, but is expensive, requires a radiochemical facility and is associated with radiation exposure to individuals and the environment [24,25]. Optical imaging has the highest sensitivity and resolution but is limited to superficial applications due to light scattering and tissue absorption [26].

In general, each tissue-targeted pharmaceutical (Figure 1B) is composed of a vector (e.g., a tumor-selective antibody, peptide or small molecule), directing the drug to the target tissue and a conjugated effector molecule containing the tracer to be detected by the imaging method (e.g., a radioisotope or a dye) (Figure 1C). This concept depends on suitable tumor markers such as PSMA or integrins and high-affinity binders [27,28,29]. The in vivo efficacy of tumor-targeting probes is affected by the molecular structures of the effector and the targeting vector. While the importance of the latter for tissue targeting is obvious, the effects of the effector moiety for the pharmacokinetic properties of the probe are often neglected. However, effectors such as metal chelators or dyes contribute significantly to the overall molecular mass of the probe and can therefore have a large influence on its pharmacokinetic properties. Their replacement by smaller effector groups is thus attractive.

X-ray fluorescence imaging (XFI) is a non-invasive alternative method with a much higher penetration depth compared to optical imaging [30]. In addition, it provides quantitative results for the tracer analyte [31,32]. XFI is based on the emission of characteristic photons in the hard X-ray range following excitation by a scanning X-ray beam. The method does not require radioisotope labeling of targeted pharmaceuticals but simply medium- to high-Z-elements in the structure of interest. Moreover, it allows the highly sensitive detection of several elements simultaneously and has thus a unique multi-tracking capability [33]. Iodine is particularly interesting for applications in this context, because it can easily be introduced into targeted pharmaceuticals [34]. In contrast to other bulky tracers such as dyes or radiometal chelates, the introduction of iodine has only a minor impact on the overall size and pharmacokinetic properties of the drug.

XFI has been used recently for imaging in vitro [35,36,37,38] and also in vivo [39]. A major limitation for a broader application of XFI in tumor imaging is the fact that measurements of highest sensitivity are currently only possible at synchrotron facilities, which provide only limited access and are impractical for clinical applications [40]. Therefore, more compact X-ray sources have been developed in recent years, either based on the novel laser–plasma accelerator technology, or on conventional X-ray tubes [40]. First-demonstration studies using a benchtop system combining XFI and CT (XFCT) showed a substantially higher detection sensitivity of XFCT compared to CT imaging alone [41]. This report describes the combination of these technical advances with the design of new targeted pharmaceuticals for the tumor marker PSMA. The resulting conjugates allow the targeting of PSMA+ prostate cancer cells with probes of low molecular weight. In addition, they are valuable tool compounds for nano-biochemistry.

## 2. Results

**Scheme 1:**
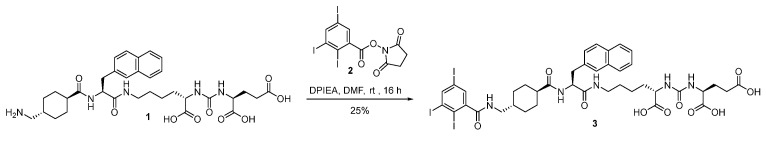
Synthesis of PSMA targeted XFI tracer **3**.

XFI demands medium- to high-Z elements with sufficiently high energetic characteristic emissions like I, Cd or Pd. Apart from the tracer element used, it is also advantageous to attach multiple tracer atoms to a targeting vector, to increase the sensitivity of detection [42]. This is often achieved by the conjugation of nanoparticle tracers (e.g., Pd or Au nanoparticles) [36]. Alternatively, targeted pharmaceuticals of low molecular weight might also contain more than one heavy tracer element. At this point, it is notable that iodine has ideal emission properties for XFI detection and is easy to introduce into pharmaceuticals of low molecular weight [43]. As a suitable targeting vector, a fragment of the known radiopharmaceutical PSMA-617 was chosen. PSMA-617 contains a urea motif for prostate cancer targeting and a conjugated macrocyclic chelator bearing the radiometal for PET imaging [18]. Replacing the chelator with triiodobenzoic acid (TIBA) as an effector yields a potential PSMA-directed XFI tracer with three iodine atoms. This compound was first evaluated in silico for PSMA-binding. Docking experiments of PSMA-617 (as a reference compound) and the new compound **3** were performed with two different protein structures (3BI1 and 4mcp) (Figure S2). The docking scores for both compounds were comparable, indicating that the introduction of the triiodobenzoyl motif in **3** does not compromise PSMA-binding (see Figure S1 for docking poses and scores). The synthesis of XFI tracer **3** was achieved by the active ester coupling of triiodobenzoate NHS-ester **2** to the targeting urea motif of PSMA-617 **1**, as outlined in Scheme 1 (for a detailed synthetic scheme including starting materials see Scheme S1).

### 2.1. XFI Measurements with Prostate Cancer Cell Lines

**Figure 2:**
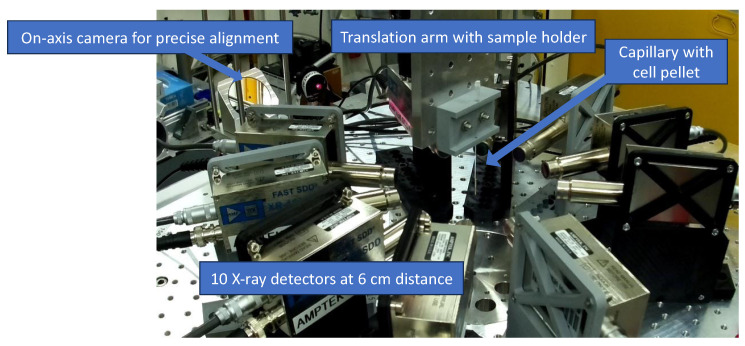
A picture of the custom-built experimental setup at the P21.1 beamline, consisting of 10 radiation detectors, on a platform which allows the scanning of samples through the incident X-ray beam. The on-axis camera on the left side allows for the precise alignment of the samples and shows the direction of the incident X-ray beam.

To investigate the cell binding of **3**, in vitro assays were performed with PSMA(+) and PSMA(−) PC3 cells. Briefly, cells were incubated with a 50 nm solution of **3** for 30 min and subsequently washed thoroughly with PBS to remove all unbound compounds. The cell pellets were finally measured by XFI after centrifugation. The iodine content of each cell line was determined via XFI post-incubation at the P21.1 beamline of the PETRA III synchrotron (DESY, Hamburg, Germany). A monochromatic incident X-ray energy of about 53 keV was used, which is a good compromise between background reduction on the one hand and a fluorescence cross-section on the other hand, as described in detail in the literature [39]. The prepared cell pellets were mounted on a dedicated holder to allow scanning through the incident X-ray beam of a 1 mm × 1 mm area, resulting in a spatial resolution of 1 mm. A total of ten silicon drift detectors (SDDs) of 50 mm^2^ collimated area and 1 mm sensor thickness from Amptek (XR-100 FAST SDD, Amptek Inc., Bedford, MA, USA) were positioned around the target at a distance of 6 cm from its center and under detection angles ranging from 30° up to 150° in 30° step sizes to cover a large fraction of the solid angle as fluorescence photons were emitted isotropically. Figure 2 shows a picture of the experimental setup, which consisted of a custom-made imaging platform with a translation arm, to allow the scanning of the centrifuged cell pellets contained within thin glass capillaries.

**Scheme 2:**
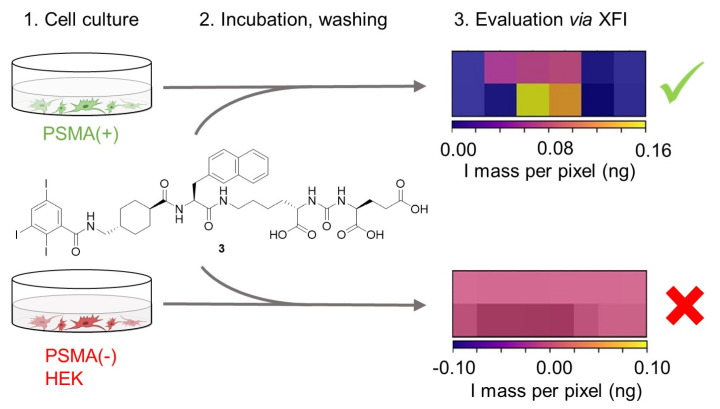
Schematic drawing of cell assay with HEK, PSMA(+/−) cell lines, tracer **3** and XFI determination of iodine. Both maps show reconstructed iodine mass per pixel. Green check indicates detection of iodine in PSMA (+) cells, whereas the red x indicates no iodine detection in PSMA (−) cells.

The resulting iodine mass maps for each cell line had significantly different iodine contents, as measured by XFI (Scheme 2). Whereas roughly 0.5 ng of iodine was measured for a cell pellet with approximately 5 × 10^5^ PSMA(+) PC3 cells, no significant amounts of iodine were detected for a similar number of PSMA(−) PC3 and HEK cells. This indicates the successful targeting of PSMA with the iodine-containing targeting vector **3**.

The iodine mass of 0.5 ng observed by XFI analysis for a cell pellet with 5 × 10^5^ PSMA(+) PC3 cells after treatment with **3** corresponds to 7.88 × 10^−18^ mol I per cell. This value compares reasonably with the amount of tracer observed for similar targeting molecules by other techniques. The cellular uptake of Eu-PSMA-617 has previously been measured via ICP-MS for PSMA(+) LNCaP cells [44] and has been found in this work to be 5.54 × 10^−18^ mol Eu per cell for PSMA(+) PC3 cells. Unfortunately, a direct comparison of the cellular uptake of the same targeting ligand **3** via ICP-MS and XFI analysis is not possible. The harsh acidic and oxidative digestion necessary for ICP-MS analysis of biological samples leads to the formation of volatile iodine and thus a loss of analyte from the sample, making ICP-MS unsuitable for the trace analysis of iodine from cell samples [45,46]. However, the values obtained suggest a similar cellular uptake of both probes **3** and Eu-PSMA-617, which is reasonable because both contain the same targeting vector.

### 2.2. Iodinated Tracers for Characterization of Nanoparticle Conjugates via XFI and ICP-MS

**Scheme 3:**
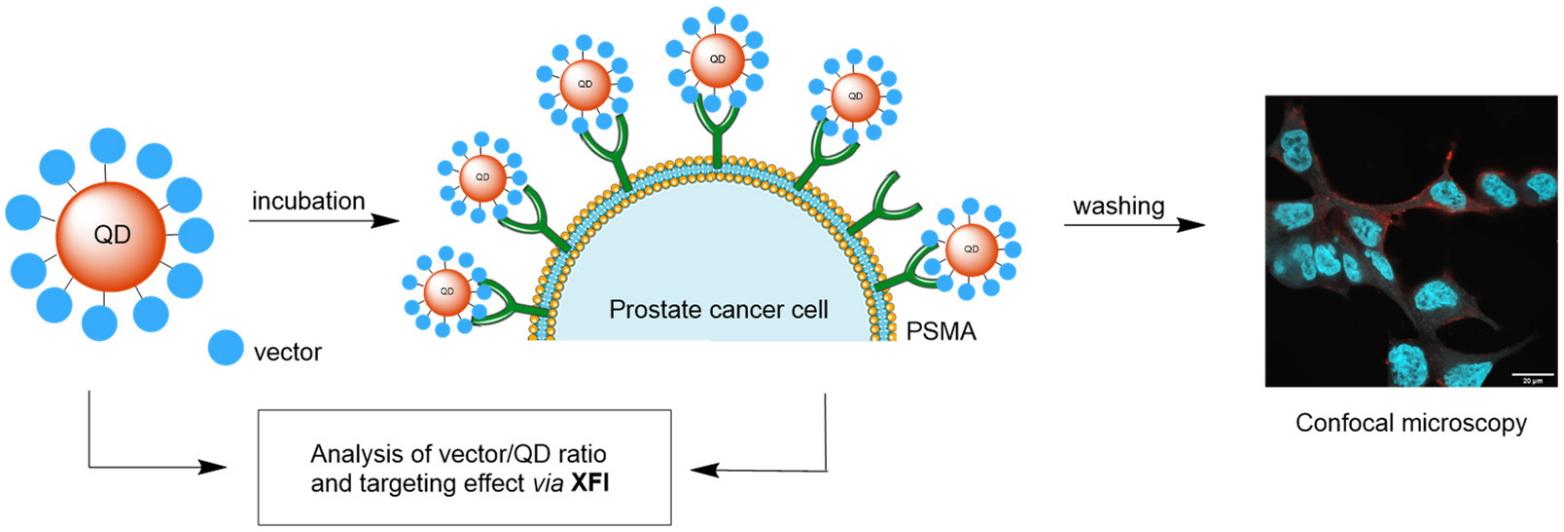
Schematic drawing of PSMA-targeted QD-based bimodal probes for detection via XFI and confocal microscopy.

Although the majority of current targeted nanoparticle (NP) systems are polymer- or lipid-based NPs [47], inorganic NPs like quantum dots (QDs) can advance the field due to their tailored size, outstanding optical properties, high photostability and excellent quantum yield [48]. Targeted QDs are typically stabilized with suitable surface ligands, providing functional groups for the conjugation of targeting vectors. The stoichiometry of this conjugation process is often hard to control but it is a key variable for biological application [49]. The loading density of the targeting vector on the particle can influence the targeting ability of the conjugate dramatically [50]. On the one hand, a high loading density may cause high-avidity binding to certain tumor-specific epitopes. On the other hand, a high loading density of targeting vectors can also decrease binding affinity to the target, as, for example, by steric or charge crowding. It is therefore important to determine the stoichiometry of the NP/vector-conjugate. XFI can be used for simultaneous multi-element analysis (multi-tracking). It therefore allows the characterization of NP conjugates if suitable elements (like Cd and I) are present in the NP and the targeting vector. Following this hypothesis, polymer-coated CdS/CdSe-based QDs (26 nm, as measured by dynamic light scattering in H_2_O, for details see Table S1) were conjugated with iodinated targeting vectors for PSMA. The targeting ability of these QD conjugates was evaluated in a cell assay with confocal microscopy. In parallel, the QD/vector stoichiometry was evaluated by the ratio between the XFI-determined content of Cd and I (Scheme 3).

**Scheme 4:**
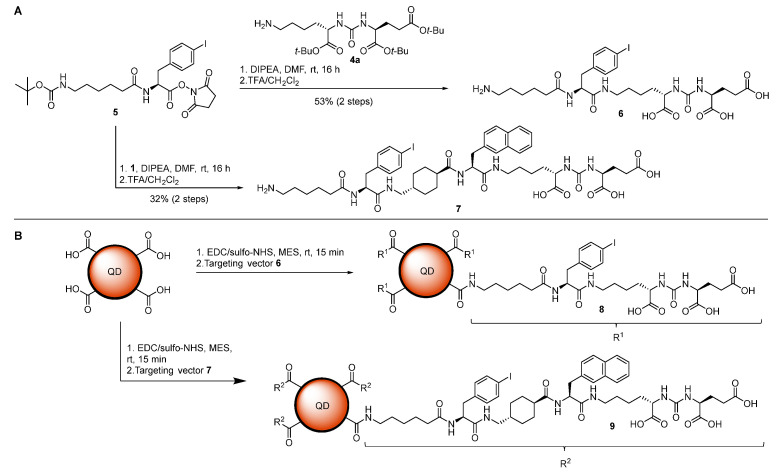
(**A**) Synthesis of iodinated targeting vectors **6** and **7** from building block **5** via active ester-mediated peptide coupling and subsequent acidic cleavage of protecting groups. (**B**) Modification of Cd/Se QDs via active ester-mediated peptide coupling of targeting vectors **6** and **7**. Black ring around QDs represents polymer coating.

Fluorescent core–shell CdSe/CdS QDs were synthesized and supplied by the Fraunhofer IAP—Center for Applied Nanotechnology, department of Quantum Materials. These hexane-dispersed particles were polymer coated and subsequently transferred into aqueous media by an established protocol [51]. The concentration was determined by UV-vis absorption spectroscopy and ICP-MS. The carboxylate-functionalized polymer coating ensured biocompatibility and allowed further modification with targeting ligands via active ester-mediated amide formation. As targeting vectors, we focused on **6** and **7**, which are both derived from previously studied PSMA-targeting motifs (Scheme 4A) [18,52]. Conjugate **7** is a derivative of PSMA-617, while **6** is derived from PSMA-11. A terminal fragment, **5,** containing the tracer element iodine and aminohexanoic acid was coupled via active ester method to **1** and **4a,** respectively. Subsequent TFA deprotection gave the free targeting vectors **6** and **7** (for a detailed synthetic scheme including starting materials see Scheme S1).

With the targeting vectors **6** and **7** in hand, conjugation to the polymer-coated QDs was achieved by the activation of the QDs with EDC/sulfo-NHS and subsequent treatment with **8** and **9** (Scheme 4B). The resulting conjugates **8** and **9** were isolated by centrifugation. Particle concentration was derived from the Cd content determined by ICP-MS (see Supplementary Materials Table S2 for calculation and particle characterization).

**Figure 3:**
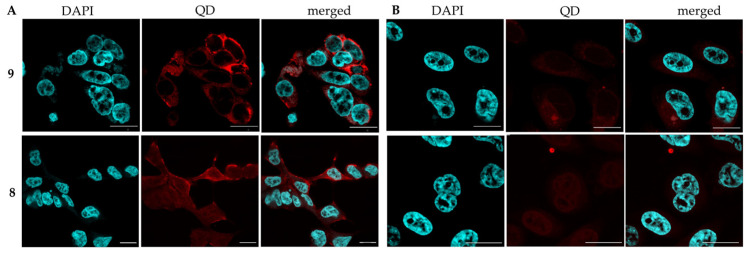
Confocal microscopy images of (**A**) PSMA(+) LNCaP and (**B**) PSMA(−) PC-3 cells after incubation with 10 nm **9** or **10**, respectively. Cell nuclei were stained by DAPI (blue); QDs appear red. Scale bar represents 20 µm.

The conjugates **8** and **9** were evaluated via confocal microscopy in a cell assay with PSMA(+) LNCaP and PSMA(−) PC3 cells (Figure 3) [53,54]. Non-functionalized QDs were used as negative control and the respective confocal images are depicted in Figure S1.

The confocal images showed that both conjugates **8** and **9** successfully targeted PSMA(+) LNCaP cells. In contrast, no cell labeling under the same incubation conditions was observed in PSMA(−) PC3 cells. The resulting data reveal a specific targeting of **8** and **9** to PSMA(+) cells. The stoichiometry of **8** and **9** was analyzed by XFI: 10 µL of a solution of the QD conjugate **9** was transferred into a glass capillary and was analyzed for its Cd and I content. This time, a prototype laboratory XFI system was used. This system allows XFI measurements without the need for synchrotron beamtimes. Though the measured XFI spectra are comparable, the laboratory system requires much longer scan times compared to the synchrotron, but offers much easier access. The measurement indicated a quantity of 2.1 µg Cd and 68.8 ng I for **8**, 2.4 µg Cd and 28.6 ng I for **9**, and 1.8 µg Cd and 0.0 ng I for QDs without conjugated targeting vectors. The Cd amounts were also analyzed via ICP-MS and found to be almost identical. However, iodine measured in ICP-MS was inconclusive, underlining the benefits of utilizing XFI for a simultaneous determination of Cd and I content. The Cd and I amounts measured correspond to a ratio of 26 targeting vectors per QD (corresponding to ~1.4 molecules per nm^2^) for **8**. A lower ratio of 11 targeting vectors per QD (~0.6 per nm^2^) was determined for **9** (Table 1). The surface-coverage was derived from the TEM-determined particle size under the assumption of spherical particles (see Supplementary Materials).

## 3. Discussion

This proof-of-concept study describes the first example of targeted cancer imaging by XFI using small molecular probes. These probes make use of novel targeting vectors for the tumor marker PSMA and contain iodine as a tracer element for XFI analysis. The targeting vectors have all been assembled from a urea-based scaffold for PSMA-targeting and an iodinated acyl compound via a modular synthetic strategy. The resulting targeting probe **2** is a direct analog of PSMA-617, a known pharmaceutical for PET imaging of cancer. In the XFI-probe **2**, the metal chelator of PSMA-617 is substituted for a triiodobenzoyl group. Probe **2** was evaluated in an in vitro cell assay and the iodine content of each incubation experiment was determined using XFI. The iodine mass maps revealed the successful targeting of PSMA(+) cell lines, determining approximately 7.88 × 10^−18^ mol iodine per cell, whereas PSMA(−) cells showed no significant enrichment of iodine. The visualization and quantification via XFI indicates the successful targeting of PSMA with the iodine-containing probe **2** and suggests a similar binding behavior of this compound to PSMA compared to Eu-PSMA-617. For the latter compound, 5.54 × 10^−18^ mol Eu tracer per cell was determined by ICP-MS analysis. This number is comparable to the value obtained by XFI for analog **2** in this study. No accumulation of the analyzed elements was found in PSMA(−) cells in both assays, confirming the specificity of the probes for PSMA. To the best of our knowledge, this is the first successful imaging of targeted small molecules via XFI in a cell model. Being able to use targeted small-molecule probes has a clear advantage over existing methods for XFI imaging (e.g., with nanoparticle tracers) with respect to pharmacokinetics. Given the rapidly evolving field of XFI, we assume that this technique will be useful for ligand screening, cellular and tissue distribution studies or even in vivo imaging without radioactive tracer molecules in the future.

Furthermore, XFI was utilized as a characterization tool for NP conjugates. The loading of targeting vectors on NPs is an important parameter for targeted drug delivery systems and targeted imaging probes. Despite the importance of vector loading on NPs for the study of multimerization effects, the determination of the NP/vector stoichiometry is a challenging task. To address this issue, two iodine-labeled targeting vectors, **6** and **7,** were conjugated to QDs. In vitro assays with the QD conjugates revealed the successful targeting of the QD/vector conjugates **8** and **9** in confocal microscopy images of PSMA(+) cells, while no enrichment of the targeting probes was observed in PSMA(−) cells. This indicates a specific binding of probes **8** and **9** to PSMA(+) cells. It is notable that the QD/vector conjugates **8** and **9** have been found to be non-toxic towards PC3 and LNCaP cells (Table S3) up to a 50 nM concentration, while the incubation for the confocal cell imaging was performed at 10 nM concentrations. In addition, XFI analysis of the QD/vector conjugates revealed the Cd/I ratio of the probes. Assuming a homogenous distribution of targeting vectors on the QDs, the XFI-determined surface densities of targeting vectors for probes **8** and **9** were 26 per particle versus 11 per particle. The loading of targeting vectors is thus slightly different on both probes **8** and **9**. The somewhat lower loading of **9** on the QDs might be due to the higher steric demand of this ligand compared to **8**. It is interesting that this difference in vector loading seems to have an impact on the binding properties of the resulting particles. The conjugate **8** (higher surface loading with targeting vector **7**) showed significantly less intensive staining of PSMA(+) cell lines than **9,** although the PSMA-targeting motif of both probes **8** and **9** is almost identical. Steric or charge crowding of targeting ligands might be the reason for this effect. This finding underlines the importance of the NP/vector stoichiometry for biological applications. The effect of NP/vector loading on targeting efficacy deserves further investigation, because multimerization effects have been reported to have an influence on the binding affinity of targeting vectors to PSMA [54]. It should be noted that the tool compounds **6** and **7** allow the averaged evaluation of the NP/vector ratio only. It thus provides useful information on the estimated loading of vectors per NP, but it does not exclude a possible inhomogenous distribution of vectors per NP.

Overall, these findings demonstrate the potential of XFI as a non-invasive detection method for targeted applications with small molecules and as a tool for particle analysis. The incorporation of appropriate iodine tracers can be achieved by simple chemical conjugation techniques. XFI thus allows the evaluation of the cellular uptake of small molecules and is therefore useful for the evaluation of ligands for tumor targeting without the need for radioactive tracers. The iodinated probes presented specifically addresses the cell surface marker PSMA and thus the cells expressing this marker. In consequence, these cells can be selectively detected via XFI. This opens the door for targeted cancer (or, more generally, disease) imaging with XFI tracers, much like the current clinical state of the art for cancer imaging, which uses radiotracers for techniques like positron emission tomography. With the dynamic development in instrumentation, a transfer to in vivo applications in laboratory setups is also on the horizon. Furthermore, XFI allows the otherwise challenging evaluation of surface modifications on nanoparticles stimulating the development of theranostics and the design of targeted nanoparticle conjugates.

## 4. Materials and Methods

General. Reagents and starting materials were purchased by commercial sources and used without further purification. All non-deuterated organic solvents were purchased from VWR Chemicals in HPLC grade (VWR International GmbH, Darmstadt, Germany). Deuterated NMR solvents were purchased from Deutero (Deutero GmbH, Kastellaun, Germany). Water was purified with a Merck Millipore Mili-Q filter system. Reversed-phase column chromatography was performed on C18 ec silica (Macherey & Nagel GmbH & Co. KG, Düren, Germany, 100–50 µm). NMR measurements were performed on a Bruker Avance I 400 MHz and a Bruker Avance I 500 MHz. Chemical shifts (δ) are expressed in parts per millions (ppm) (Bruker BioSpin, Ettlingen, Germany). Analytical HPLC-MS was performed on an Agilent HPLC System 1260 Infinity II (Agilent Technologies, Waldbronn, Germany) with an EC 150/2 Nucleodur C18 HTec, 5 μm, linked to a Bruker ESI mass spectrometer. HRMS was performed on Bruker MicroTOF-Q II. For HRMS and LC-MS chromatograms, the Agilent 6224 ESI-TOF was coupled to an Agilent HPLC 1200. ICP-MS measurements were performed in triplicate on an Agilent Technologies 7700 series ICP-MS. Before the measurement took place, the ICP-MS setup was calibrated with a freshly prepared serial dilution of Cd and I (Carl Roth). The calibration curve was constructed using different elemental concentrations starting from 0 to 500 ppb (parts per billion, equivalent to μg/L).

Synthesis of triiodobenzoic acid-labeled targeting vector **3**. The starting materials **1** and **2** were synthesized according to slightly modified procedures from the literature [18,55]. Detailed protocols are listed in the Supplementary Materials. The amine **1** (3.0 mg, 4.5 µmol, 1 eq) was dissolved in 2 mL dry DMF. After the addition of DIPEA (12.5 µL, 72.0 µmol, 16 eq), the active ester **2** (3.8 mg, 6.4 mmol, 1.4 eq) was added. The reaction was stirred under N_2_ for 24 h. After removing the solvent in vacuo, the residue was dissolved in H_2_O/CH_3_CN with 0.1% HCO_2_H and purified by column chromatography on RP-18 silica gel (H_2_O/CH_3_CN with 0.1% HCO_2_H). The product was obtained as a colorless powder (1.3 mg, 1.14 µmol, 25%).

Synthesis of iodine-labeled targeting vector **6**. Active ester **5** (70 mg, 0.14 mmol, 1 eq) was dissolved in 15 mL dry DMF. After the addition of DIPEA (50 µL, 0.16 mmol, 2 eq), compound **4a** (95 mg, 0.16 mmol, 1.1 eq) was added. The reaction was stirred under N_2_ for 16 h. After removing the solvent in vacuo, the residue was dissolved in H_2_O/CH_3_CN with 0.1% HCO_2_H, purified with flash column chromatography and subsequently dissolved in CH_2_Cl_2_/TFA (3 mL, 1:1). The reaction was stirred at rt for 3 h, before the solvent was removed with N_2_ and the residue was dissolved in CH_2_Cl_2_. The solution was neutralized by the addition of NEt_3_. After removing the solvent in vacuo, the residue was dissolved in H_2_O/CH_3_CN and purified by column chromatography on RP-18 silica gel (H_2_O/CH_3_CN with 0.1% HCO_2_H). The title compound **6** was obtained (52 mg, 0.074 mmol, 67% (over two steps)) as a colorless powder.

Synthesis of iodine-labeled targeting vector **7**. The free amine **1** (13.8 mg, 0.021 mmol, 1 eq) was dissolved in 2 mL dry DMF. After the addition of DIPEA (7 µL, 0.042 mmol, 2 eq), the active ester **5** (12.8 mg, 0.023 mmol, 1.1 eq) was added. The reaction solution was stirred for 16 h under a N_2_ atmosphere. After the removal of the solvent in vacuo, the residue was purified by column chromatography on RP-18 silica gel. The product was obtained as a colorless powder (8.3 mg, 0.007 mmol, 32%), from which a portion (5 mg, 0.004 mmol) was subsequently dissolved in CH_2_Cl_2_/TFA (1:1, 2 mL). The reaction solution was stirred for 3 h at rt, before the solvent was removed under a N_2_ stream. The residue was dissolved in CH_2_Cl_2_ and the solution was neutralized by adding NEt_3_. After the removal of the solvent in vacuo, the residue was purified by column chromatography on RP-18 silica gel. A colorless powder of **8** (4 mg, 0.004 mmol, quant.) was obtained.

Cell culture. The PSMA expressing prostate cancer cell line LNCaP was obtained at DSMZ (Deutsche Sammlung von Mikroorganismen und Zelllinien #ACC 256) and grown in RPMI 1640 medium supplemented with FCS (10%), penicillin (1%) and streptomycin (1%). The HEK cell line was obtained at DSMZ (Deutsche Sammlung von Mikroorganismen und Zelllinien #ACC 305) and grown in DMEM medium. The prostate cancer cell line PC3 was obtained at DSMZ (Deutsche Sammlung von Mikroorganismen und Zelllinien #ACC 465) and was grown in RPMI 1640/Ham F12 (1:1) medium supplemented with FCS (10%), penicillin (1%) and streptomycin (1%). The PSMA(+) cell line was obtained from Udo Schumacher. Details can be found in the literature [56,57]. The presence of the gene for the expression of PSMA was confirmed by quantitative PCR.

Cell assays for XFI: Cell incubation was performed at 37 °C under 5% CO_2_, while the assay was performed at rt. The experimental procedure was as follows: first, cells were seeded in 6-well plates using 100,000 PSMA(−) PC3 cells or 100,000 PSMA(+) PC3 cells. Subsequently, the cells were incubated for 72 h in 2.5 mL medium and washed once with 1 mL PBS, before treatment with a 50 nM ligand dissolved in medium for 30 min. After incubation, the wells were washed three times with 1 mL PBS and harvested in 0.5 mL PBS. The cells were counted using a Neubauer counting chamber and 5 × 10^5^ cells were then centrifuged at 2000 rpm for 3 min and the supernatant was removed. The cells were resuspended in 0.01 mL PBS and transferred to glass capillaries. A further centrifugation at 1000 rpm for 1 min was performed before the cell pellets were measured by XRF.

Synthesis of QD conjugates: The phase transfer into aqueous media was performed according to an established protocol [58] by mixing hexane-dispersed CdSe/CdS QDs (1000 µL, 3.08 µm, for details see Supplementary Materials) with poly-(isobutylene-alt-maleic anhydride)-graft-dodecyl (PMA, 1353 µL, 0.5 m in CHCl_3_) in a 4 mL glass vial. The solution was subsequently diluted with CHCl_3_ (1000 µL) and was then moved overnight on a shaker at rt. After that, CHCl_3_ was removed in vacuo to dryness. To transfer the polymer-coated QDs into an aqueous solution, NaOH (2000 µL, 0.1 m) was added and sonicated for 15 min. Aliquotes (1000 µL) of dissolved particles were collected in 1.5 mL Eppendorf tubes. The procedure was repeated until the particles were completely dissolved. All collected aliquots were then centrifuged (30,000 g, 1 h, 15 °C). The supernatant was again centrifuged (30,000 g, 1 h, 15 °C) to maximize the yield. All pellets were combined, dissolved in H_2_O (1000 µL) and centrifuged (30,000 g, 1 h, 15 °C). The resulting supernatant was removed and the resulting pellet was dissolved in H_2_O, yielding polymer-coated QDs with carboxylates for further conjugations.

QD conjugates **8** and **9** were synthesized by mixing polymer-coated QDs (1 eq) with EDC (10000 eq) and sulfo-NHS (10000 eq) in 2-(*N*-morpholino)ethanesulfonic acid (MES) buffer for 15 min at rt. The dispersion was filtered through 30 kDa MWCO centrifuge filters (8000 rfc, 8 min) and dispersed in PBS (200 µL). The procedure was repeated again and the resulting dispersion was mixed with the respective targeting vector (**6** or **7**, 5000 eq) and was moved on a shaker for 16 h at rt. Finally, the dispersion was filtered through 30 kDa MWCO centrifuge filters (8000 rfc, 8 min, rt) and dispersed in PBS (200 L) three times. The concentrations of the resulting particles were determined by both ICP-MS and XFI.

Confocal microscopy measurements. Confocal microscopy images were taken on an Olympus FV3000. Images were performed with an excitation wavelength of 405 nm, with a maximum emission at 601–680 nm for quantum dots and maximum emission at 415–472 nm for DAPI.

Cell assays for confocal microscopy. Cells were incubated at a temperature of 37 °C in a 5% CO_2_ atmosphere. The assay was performed at rt. Initially, the cells (either 50,000 PSMA(+) LNCaP cells or 50,000 PSMA(−) PC-3 cells) were plated in 4-well slides followed by an incubation period of 24 h. The cells were then washed twice with PBS and fixed by adding paraformaldehyde (4%) for a period of 15 min, followed by washing the slides twice with PBS. To permeabilize the cell membrane, the cells were treated with Triton (0.2%), and after two more washes with PBS, the cells were treated with a blocking solution of 1% BSA in PBS for 30 min. The cells were incubated with a 10 nm solution of the QDs for 30 min. After washing the slides three times with PBS, the cell nuclei were stained using DAPI. After washing the slides again three times with PBS, each well was mounted with a drop of SlowFade Gold Antifade Mountant (Invitrogen) and sealed with a coverslip for subsequent confocal measurements.

## Figures and Tables

**Table 1:** Measured I and Cd masses and calculated ratios of I and Cd in 10 µL aliquots, as determined by XFI and ICP-MS for all applied substances.

Sample	Mass Cd by XFI (µg)	Mass I (ng)	Mass Cd by ICP-MS (µg)	Ratio (I/QD)
8	2.1	68.8	1.7	26
9	2.4	28.6	2.4	11
QDs	1.8	0	1.8	0

## Data Availability

The data that support the findings of this study are available from the corresponding author, upon reasonable request.

## Supplementary Materials

The following supporting information can be downloaded at https://www.mdpi.com/article/10.3390/ijms252211880/s1. References [59,60,61] are cited in the Supplementary Materials.
