# Supplementary material for: Iodinated PSMA Ligands as XFI Tracers for Targeted Cell Imaging and Characterization of Nanoparticles
Source: Int J Mol Sci. 2024 Nov 5;25(22):11880. doi: 10.3390/ijms252211880 (PMC11594147; doi:10.3390/ijms252211880)
Supplement: Supplementary file 1 [file ijms-25-11880-s001.zip › ijms-2956600-supplementary.pdf]

Supporting Information

# Table of Contents


Quantum Dots ........ 2
Determination of QD concentration from mass concentration of Cd ........ 3
ICP-MS data ........ 3
Cytotoxicity studies ........ 4
Confocal measurements of QDs ........ 4
qPCR of HEK, PC3 (-), PC3 (+) and LNCaP cells ........ 5
Scheme S1. Synthesis of the targeting vectors (3, 6, 7) ........ 6
Urea (1) ........ 7
TIBA-NHS (2) ........ 10
TIBA-PSMA 617 (3) ........ 12
Boc-AHX-IPA-NHS (5) ........ 13
Urea (Boc-6) ........ 15
Urea (6) ........ 17
Urea (7) ........ 20
Urea (1a) ........ 21
Urea (1b) ........ 23
Urea (1c) ........ 24
Urea (1d) ........ 27
Boc-Naphtylalanin (II) ........ 28
Boc-Naphtylalanin-AE (III) ........ 30
Boc-Tranexamic acid (V) ........ 32
Boc-Tranexamic acid-NHS ester (VI) ........ 34
Boc-AHX-NHS (VII) ........ 36
Boc-AHX-IPA (VIII) ........ 38
Docking studies ........ 40
References ........ 40

## Quantum Dots

Giant-shell CdSe/CdS Particles were synthesized by the Fraunhofer IAP – Center for Applied Nanotechnology (CAN). Detailed characterization data is listed in Table S1.

**Table S1.** Characterization data and visualization of the utilized quantum dots. The absorption and emission spectra are shown as well as TEM images of the particles.

| Data | | |
|---|---|---|
| LOT number: Cd-0-288-2fz | | |
| **Absorption spectroscopy** | | |
| Absorption maximum | HWHM | |
| - | - | |
| | | |
| **Fluorescence Spectroscopy** | | |
| Emission-maximum | FWHM | Quantum Yield[1] |
| 623 nm | 33 nm | 84% |
| [1] **measured by integrating sphere (exc. at 450 nm)** | | |
| | | |
| **Transmission electron microscopy** | | |
| Shape | Diameter | |
| spherical | $d_{QD}$ = 13.5(±2.1) nm | |
| | | |
| **Dynamic light scattering** | | |
| Hydrodynamic radius ($H_2O$, Intensity): $R_{hyd}$ = 26 nm | | |
| | | |
| **Thermogravimetric analysis** | | |
| Mass concentration: $b_{Cd}$ = 11.5 mg/mL<br>Particle concentration: $c_{QD}$ = 3.08 µM | | |
| | | |
| **Inductively coupled plasma mass spectrometry** | | |
| Mass concentration: $b_{Cd}$ = 12.2 mg/mL<br>Particle concentration: $c_{QD}$ = 3.27 µM | | |

**Visualization**

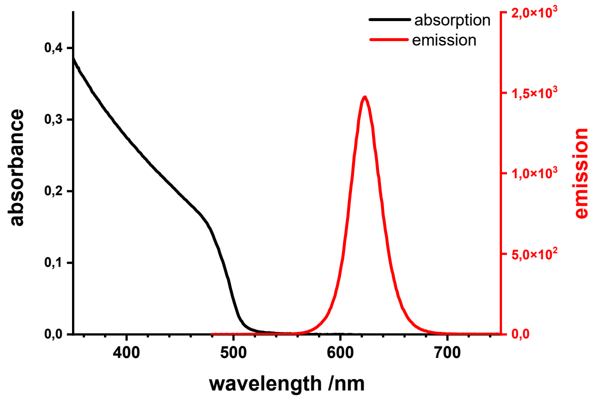


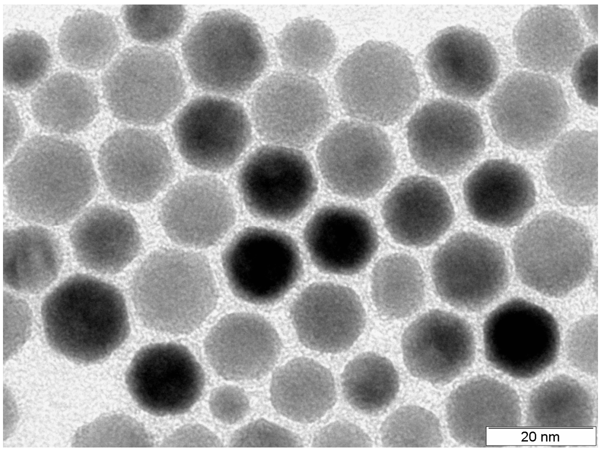


Absorbtion spectra were recorded on a Varian Cary 50 UV-vis photometer. Fluorescence spectra were measured with a Varian Cary Eclipse fluorescence spectrometer. Transmission electron microscopy images were captured on a Jeol JEM-1011 microscope. Dynamic light scattering measurements were conducted on a Malvern Zetasizer Nano ZS. Thermogravimetric analysis was performed with a Netzsch TG 209 *F1* Iris. Inductively coupled plasma mass spectrometry was performed in triplicates on an Agilent Technologies 7500cs series ICP-MS.

**Determination of QD concentration from mass concentration of Cd**

To convert the mass concentrations of Cd measured by ICP-MS and TGA into a particle concentration, a approximated Cd-contend per QD was derived from the TEM-determined particle size. Assuming spherical particles the particle Volume $V_{QD}$ was calculated from the particle diameter $d_{QD}$ (S1).

$$V_{QD} = 4/3\,\pi(d_{QD}/2)^3 \tag{S1}$$

$V_{QD}$ Particle volume ($m^3$)

$d_{QD}$ Particle size (m)

To determine the Cd-content per particle $m_{Cd}$, the Volume $V_{QD}$ was divided by the density $\rho_{CdS}$ of CdS, since it accounts for the vast majority of giant shell-particles core material. Since Cd accounts for 78% of the mass of CdS, this was factored in the calculation (S2).

$$m_{Cd} = 0.78(V_{QD}/\rho_{CdS}) \tag{S2}$$

$m_{Cd}$ Cd content per QD (g)

$\rho_{CdS}$ Density CdS (4820 kg/$m^3$)

With this, a particle concentration can be derived from ICP-MS results, by dividing the measured mass concentrations of Cd by the calculated Cd-content per particle $m_{Cd}$ and Avogardos's constant $N_A$.

$$c_{QD} = b_{Cd}/m_{Cd}N_A \tag{S3}$$

$c_{QD}$ QD concentration (mol/L)

$b_{Cd}$ Measured Cd mass concentration (µg/L)

$N_A$ Avogadro constant ($6.022\ 10^{-23}\ mol^{-1}$)

**ICP-MS data**

To guarantee the ionization of all elements and a precise measurement, all particles had to be prepared following an established protocol.[1] From each substance, aliquots (2 µL) were treated with *aqua regia* (500 µL) and left for 24 h at rt, before aqueous HCl (2%) was added *ad* 2500 µL.

**Table S2.** ICP-MS-measured Cd content with the respected Cd content in the original aliquot, as well as the Cd content in 10 µL, which mirrors the sample volume in XFI.

| **Sample** | **Cd content** (µg/L) | **RSD** | **Stock $b_{Cd}$** (µg/L) | **Cd content in 10 µL** (µg/10 µL) |
|---|---|---|---|---|
| Blank | 0.2 | 3.4 | 266.5 | 0.0 |
| **8** | 133.0 | 0.7 | 166374.2 | 1.7 |
| **9** | 195.8 | 3.4 | 244942.0 | 2.4 |
| QDs | 147.5 | 1.1 | 184533.8 | 1.8 |

**Cytotoxicity studies**

The studies were conducted according to a previously established protocol.[2] Both, PC3 ($10^4$ cells per well) and LNCaP ($2\cdot10^4$ cells per well) cells were seeded and were incubated for 24 h in a 96-well plate at 37 °C and 5% $CO_2$ with complete RPMI media (10% FBS and 1% P/S) for LNCaP cells and complete RPMI/Ham F12 media (10% FBS and 1% P/S) for PC3 cells. Afterwards a dilution series of QDs dispersed in cell media were added to the cell in triplicates. After incubation for 24 h at 37 °C and 5% $CO_2$, the QD solution was removed and a total of 100 μL from a 0.02 g/L solution of resazurin (7-hydroxy-10-oxidophenoxazin-10-ium-3-one) in cell media was added into the wells. After 4 h of incubation, the fluorescence intensity was measured (excitation 560 nm, emission 590 nm) using a Tecan infinite M200 plate-reader and i-Control 1.4 software. This test is based on the irreversible reduction of resazurin to the pink and highly fluorescent resorufin. To analyze the data, the average of the background was subtracted from the maximum value. Fluorescence values obtained from control wells containing cells incubated without the addition of QDs were considered as 100 % viable cells. The determined viability is listed in Table S33.

**Table S3.** Cytotoxicity studies of PC3 and LNCaP, incubated with a dilution series of QDs. PC3 was performed in triplicates. LNCaP was performed in duplicates.

| **Conc. $c_{Qd}$** (nM) | **Viability PC3** (%) | **Viability LNCaP** (%) |
|---|---|---|
| 0.02 | 101(±3.8) | 95(±9.4) |
| 0.05 | 102(±4.1) | 96(±4.8) |
| 0.1 | 100(±2.1) | 102(±0.4) |
| 0.2 | 100(±1.4) | 102(±3.2) |
| 0.4 | 101(±1.4) | 102(±4.8) |
| 1 | 100(±2.5) | 98(±3.3) |
| 2 | 101(±2.3) | 97(±2.0) |
| 3 | 99(±1.2) | 99(±1.8) |
| 6 | 99(±3.3) | 97(±4.0) |
| 13 | 99(±2.8) | 96(±2.4) |
| 25 | 96(±3.4) | 93(±3.2) |
| 50 | 86(±7.4) | 84 |

**Confocal measurements of QDs**

Confocal measurements of PSMA(+) LNCaP and PSMA(-) PC3 cells were performed as described in the experimental. Here, the non-conjugated QDs were used as a negative control and incubated at a concentration of 10 nM and incubated the respective cells for 30 min at 37 °C following the described proceedings in **Cell assays for confocal microscopy**.

Figure S1 shows the measured confocal images of A) PSMA(+) LNCaP and B) PSMA(-) PC3 cells after incubation with unconjugated QDs.

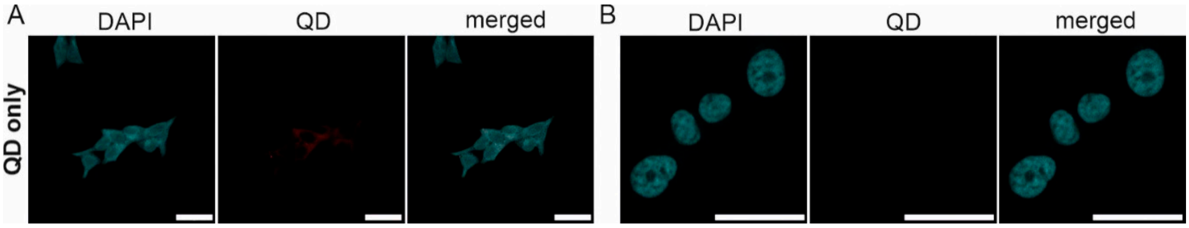


**Figure S1.** Confocal microscopy images of (A) PSMA(+) LNCaP and (B) PSMA(-) PC-3 cells after incubation with 10 nM of QDs, respectively. Cell nuclei were stained by DAPI (blue), QDs appear red. Scale bar represents 40 µm.

**qPCR of HEK, PC3 (-), PC3 (+) and LNCaP cells**

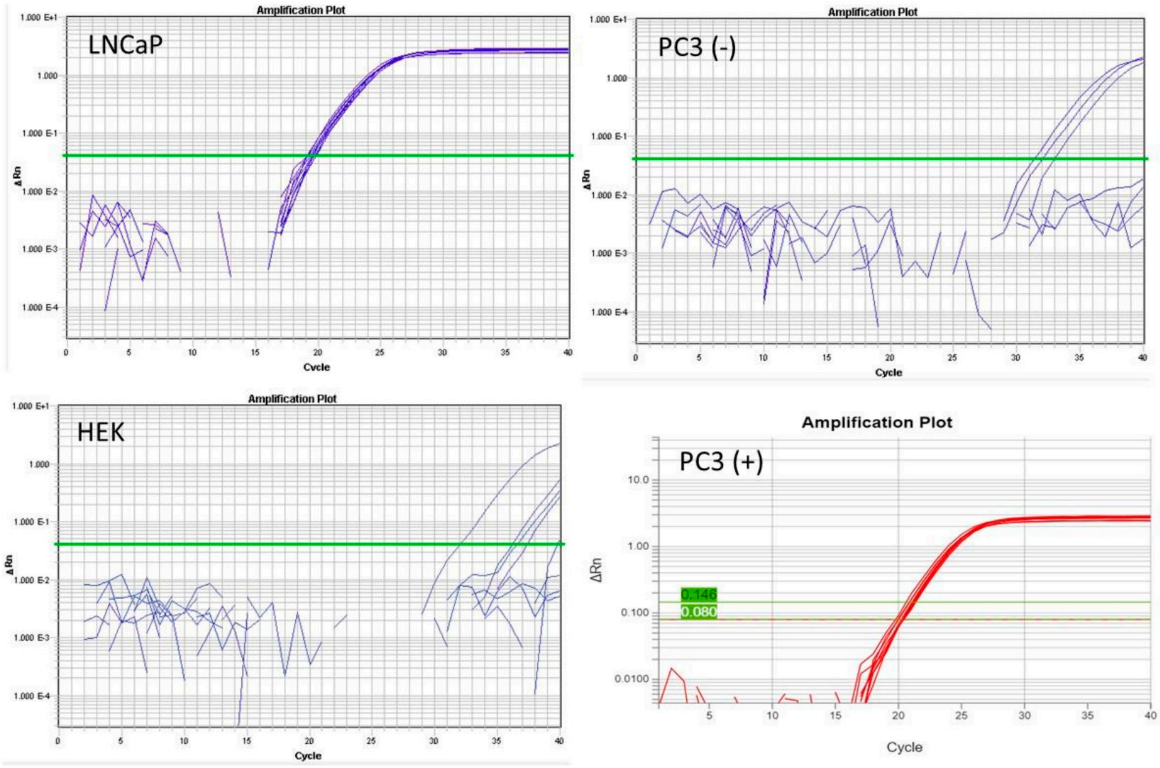

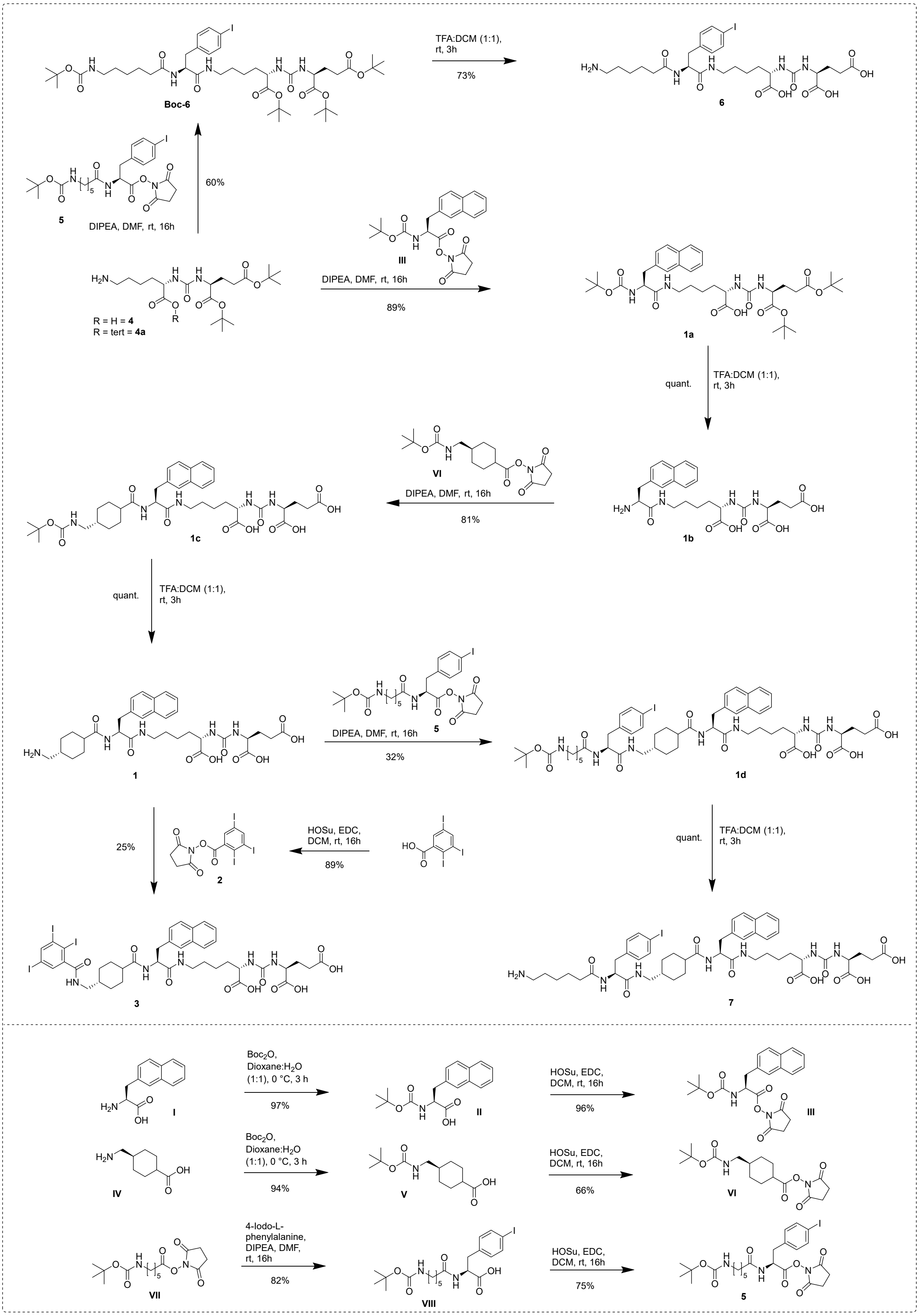


**Scheme S1. Synthesis of the targeting vectors (3, 6, 7)** and the corresponding intermediates.

**Urea (1)**

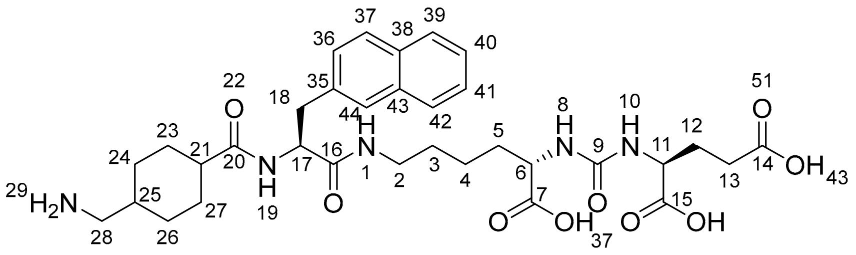

**1c** (204 mg, 0.27 mol) was dissolved in 8 mL $CH_2Cl_2$/TFA (1:1). The reaction was stirred for 3 h at room temperature. The solvent was then removed *via* an $N_2$ stream, the residue dissolved in $CH_2Cl_2$ and the solution neutralized by addition of $NEt_3$. After removal of the solvent *in vacuo*, the residue was dissolved in $H_2O/CH_3CN$ and purified by column chromatography on RP-18 silica gel ($H_2O/CH_3CN$ with 0.1% $HCO_2H$). A colorless powder of 177 mg (270 µmol, quant.) was obtained.

**$^1$H NMR** (600 MHz, DMSO): δ 11.92 (br.s., 3H, H- carbon acid), 7.99 – 7.93 (m, 2H, H-1, H-19), 7.84 (d, $^3J$ = 7.1 Hz, 1H, H-39), 7.73 (t, $^3J$ = 8.4 Hz, 2H, H-40, H-41), 7.71 (br.s., 2H, H-29), 7.68 (s, 1H, H-44), 7.48 – 7.42 (m, 2H, H-37, H-36), 7.39 (d, $^3J$ = 8.4 Hz, 1H, H-42), 6.34 (d, $J$ = 8.2 Hz, 1H, H-8), 6.30 (d, $J$ = 8.3 Hz, 1H, H-10), 4.53 (td, $^3J$ = 9.0, 5.0 Hz, 1H, H-17), 4.10 (td, $^3J$ = 8.2, 5.2 Hz, 1H, H-6), 4.01 (td, $^3J$ = 8.3, 5.0 Hz, 1H, H-11), 3.14 – 3.09 (dd, $^2J$ = 13.7 Hz, $^3J$ = 5.0 Hz, 1H, H-18a), 3.08 –2.96 (m, 2H, H-2), 2.94 – 2.89 (dd, $^2J$ = 13.7 Hz, $^3J$ = 9.6 Hz, 1H, H-18b), 2.65 – 2.55 (m, 2H, H-28), 2.37 – 2.17 (m, 2H, H-23a/27a), 2.08 (tt, $J$ = 12.2, 3.4 Hz, 1H, H-21), 1.94-1.87 (m, 1H, H-5a), 1.75 – 1.62 (m, 4H, H-5b, H-13, H-24a/26a), 1.62 – 1.54 (m, 1H, H-12a), 1.51 – 1.44 (m, 2H, H-12b, H-24b/26b), 1.44 – 1.36 (m, 1H, H-25), 1.32 (m, 2H, H-3), 1.28 – 1.16 (m, 3H, H-4, H-23a/27a), 1.07 – 0.99 (m, 1H, H-23b/27b), 0.95 – 0.80 (m, 2H, H-24b/26b).

**$^{13}$C NMR** (DMSO-$d_6$, 151 MHz): δ (ppm) 174.7 (C-20), 174.5 (C-7), 174.1 (C-15), 173.7 (C-14), 171.0 (C-16), 157.3 (C-9), 135.7 (C-Naphtyl), 132.8 (C-Naphtyl), 131.7 (C-Naphtyl), 127.8 (C-Naphtyl), 127.4 (C-Naphtyl), 127.4 (C-Naphtyl), 127.3 (C-Naphtyl), 127.2 (C-Naphtyl), 125.9 (C-Naphtyl), 125.3 (C-Naphtyl), 53.6 (C-18), 52.3 (C-17), 51.6 (C-6), 44.3 (C-11), 43.1 (C-28), 38.3 (C-21), 38.1 (C-2), 34.9 (C-13), 31.6 (C-25), 29.8 (C-44/46), 28.8 (C-5), 28.7 (C-43/47), 28.6 (C-43/47), 28.3 (C-44/46), 27.9 (C-3), 27.5 (C-12), 22.5 (C-4).

**HRMS** (ESI) *m/z* calculated for $C_{33}H_{45}IN_5O_9$: 656.3296, found: 656.3292 $[M+H]^+$.

**IR:** $\tilde{\nu}$max ($cm^{-1}$) = 3310, 2978, 2874, 2361, 2342, 1589, 1393, 1319, 1242, 1084, 961

**DC:** $R_f$ = 0.20 ($CH_2Cl_2$/MeOH/ 1% AcOH 5:1, v/v, ninhydrin).

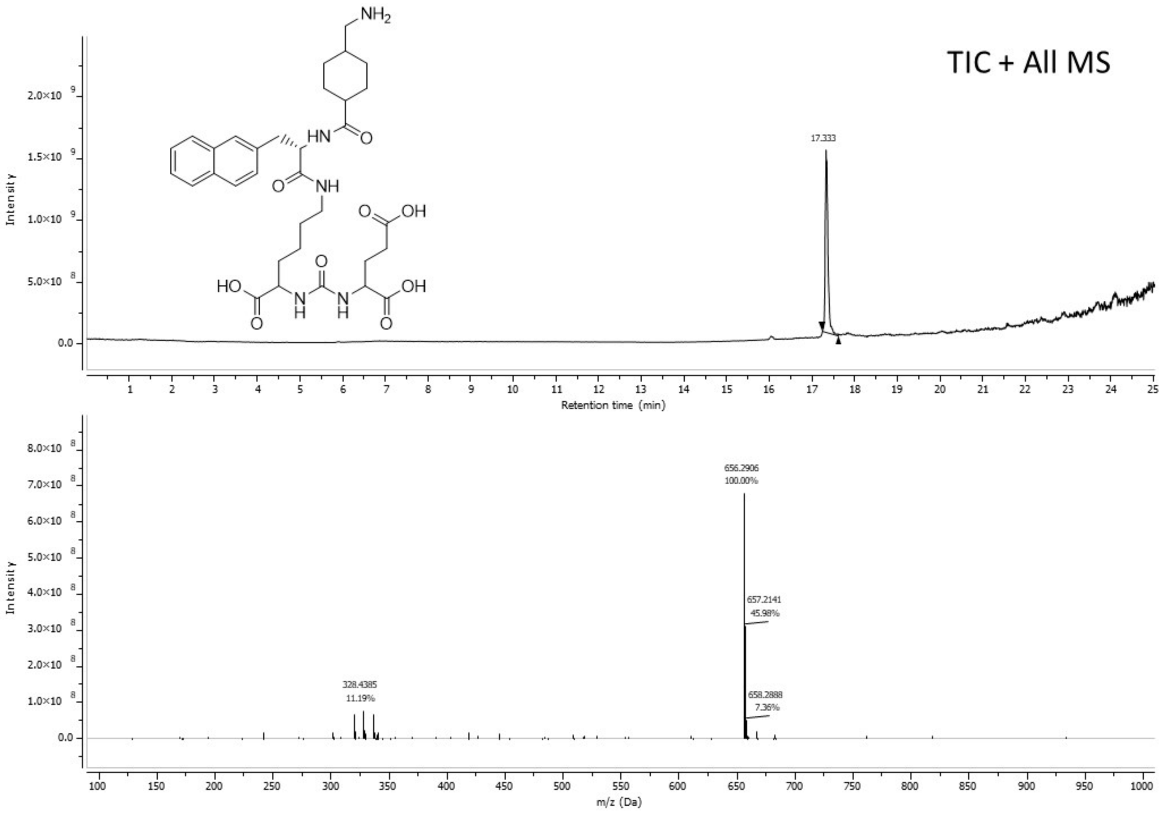


HPLC conditions: EC 150/2 Nucleodur C18 HTec, 5 µm column from Macherey & Nagel (4.6 × 100 mm) was used as stationary phase and the following gradient of MeCN in $H_2O$ with 0.1 % formic acid was used as mobile phase:

| Time | $H_2O$ | MeCN | Flow |
|---|---|---|---|
| 3 | 95 | 5 | 0.25 |
| 20 | 5 | 95 | 0.25 |
| 21 | 95 | 5 | 0.25 |
| 25 | 95 | 5 | 0.25 |

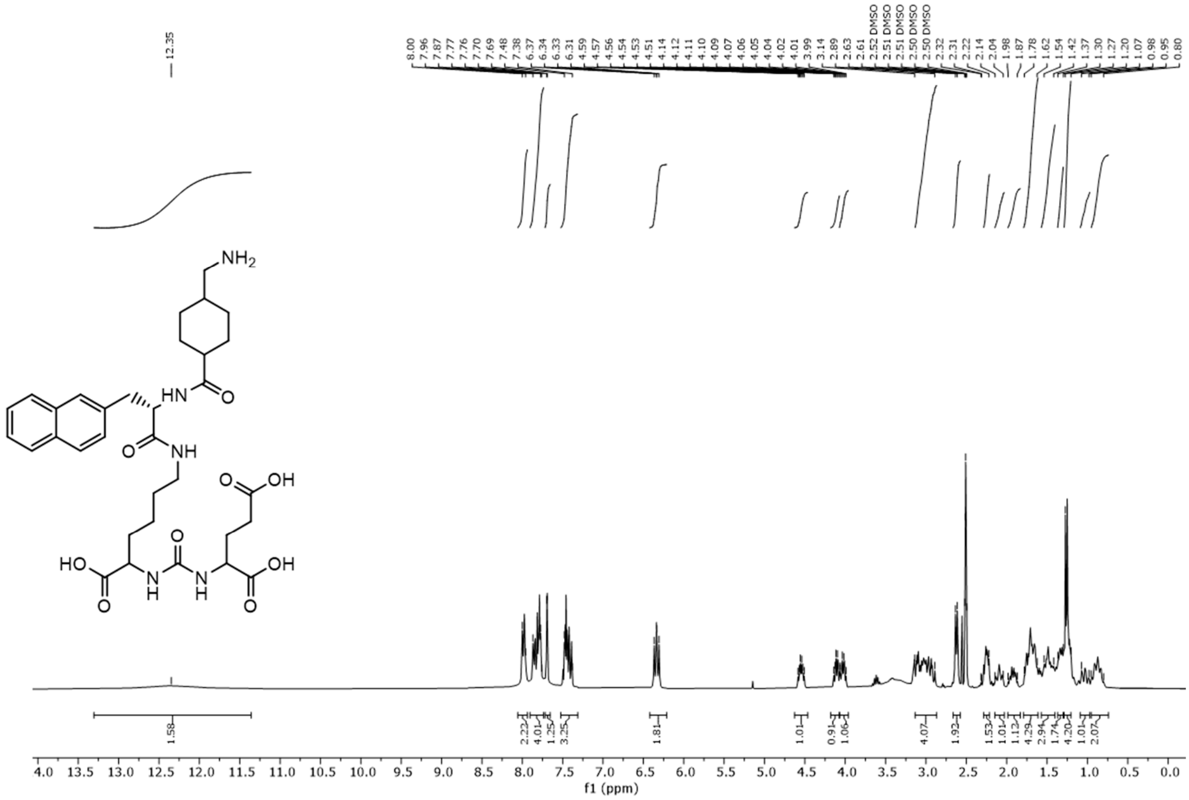
f1 (ppm)

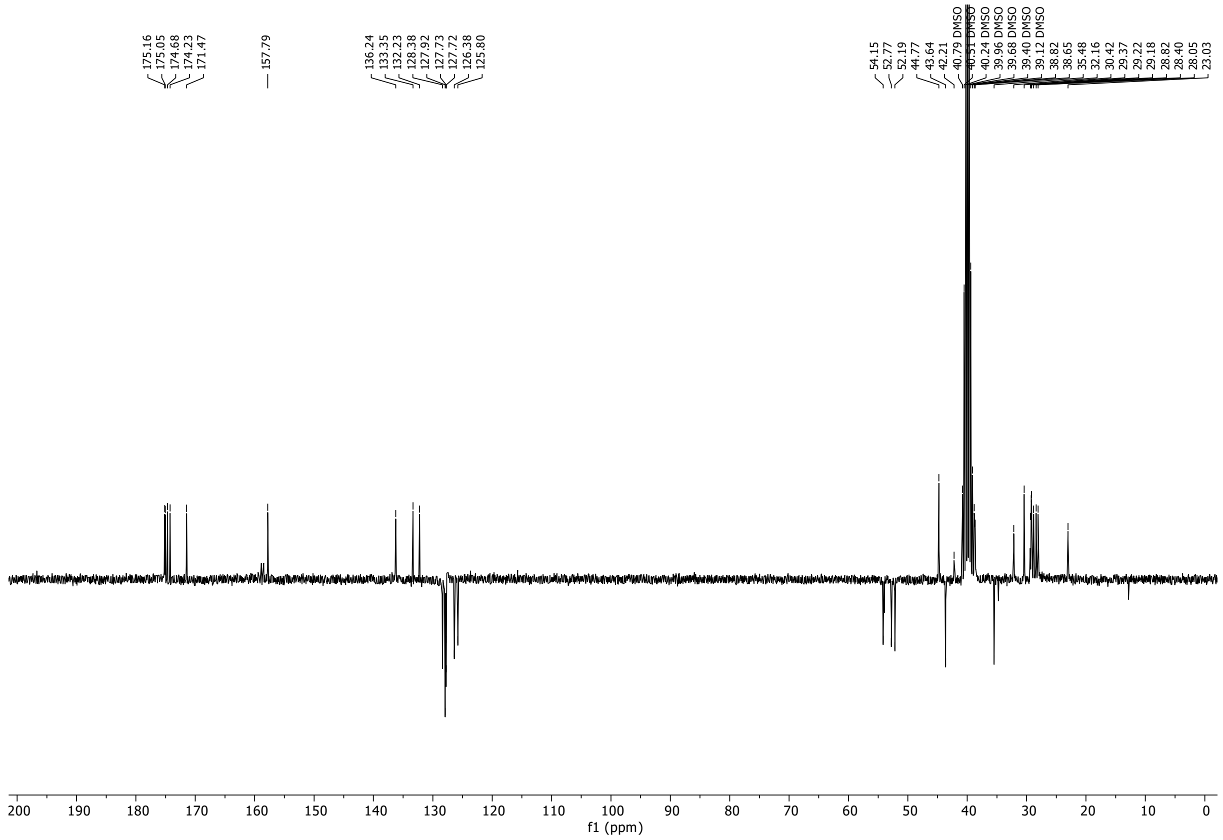
f1 (ppm)

**TIBA-NHS (2)**

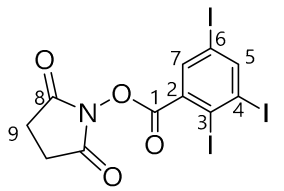


Triiodobenzoic acid (TIBA) (1.04 g, 2.09 mmol, 1 equiv.) was dissolved with *N*-hydroxysuccinimd (280.8 mg, 2.44 mmol, 1.44 equiv.) in dry $CH_2Cl_2$ (30mL). *N*-(3-dimethylaminopropyl)-*N′*-ethyl-carbodiimidehydrochloride (EDC) (467.8 mg, 2.44 mmol, 1.47 equiv.) was added to the solution and stirred under nitrogen atmosphere overnight at room temperature. The mixture was washed with $H_2O$ (3 x 10 mL), the combined organic phases were dried over $Na_2SO_4$ and the solvent was then removed under reduced pressure. The mixture was purified on silica gel (pentane/EtOAc 4:1 - 1:1) and the product was obtained as a white powder (1.09 g, 1.83 mmol, 89%).

**$^1$H NMR** (400 MHz, Chloroform-*d*) δ = 8.43 (d, *J* = 2.0 Hz, 1H, H-5), 8.06 (d, *J* = 2.0 Hz, 1H, H-7), 2.91 (s, 4H, H-9).

The impurity at 5.3 ppm is $CH_2Cl_2$.

**$^{13}$C NMR** (126 MHz, $CDCl_3$) δ = 169.0 (C-8), 161.3 (C-1), 151.1 (C-2), 138.7 (C-7), 135.8 (C-4), 114.8 (C-6), 108.4 (C-3), 93.9 (C-6), 26.1 (C-9).

$^1$H-NMR spectrum

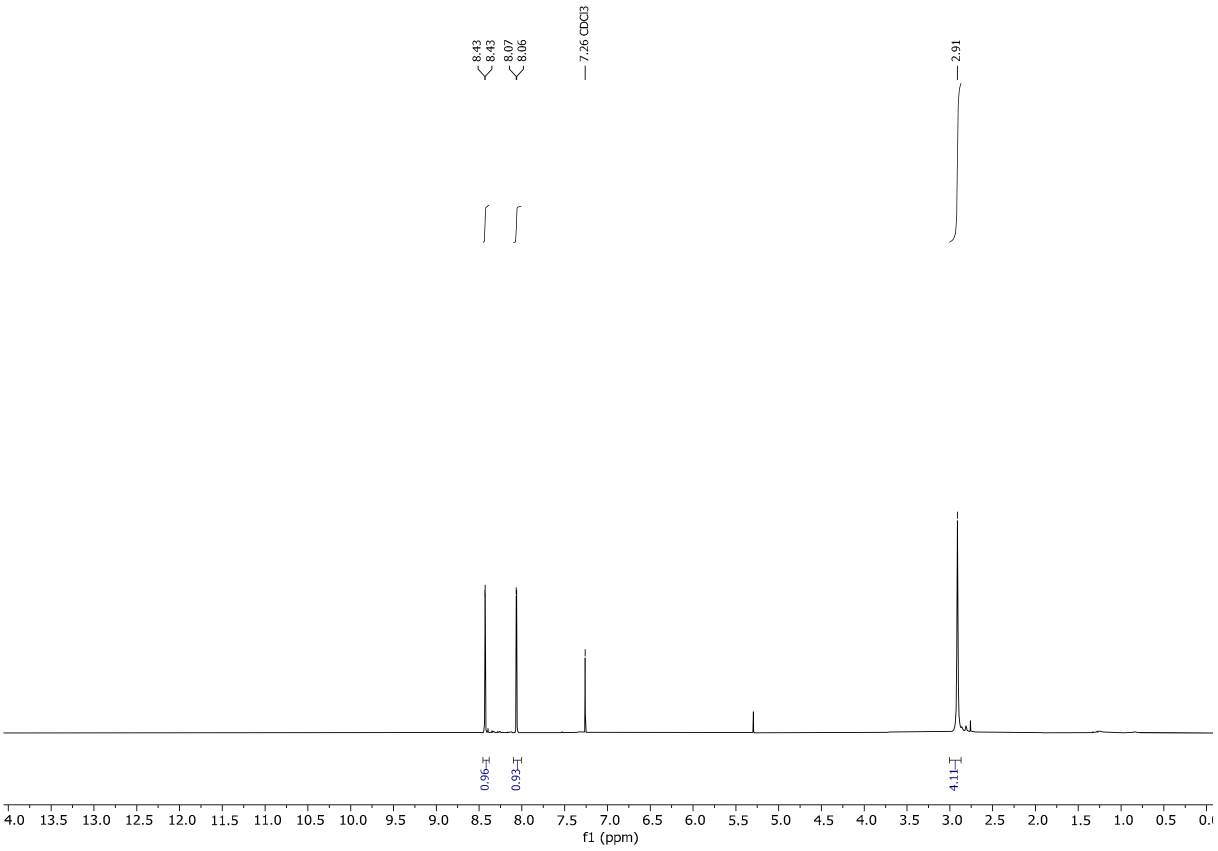


$^{13}$C-NMR spectrum

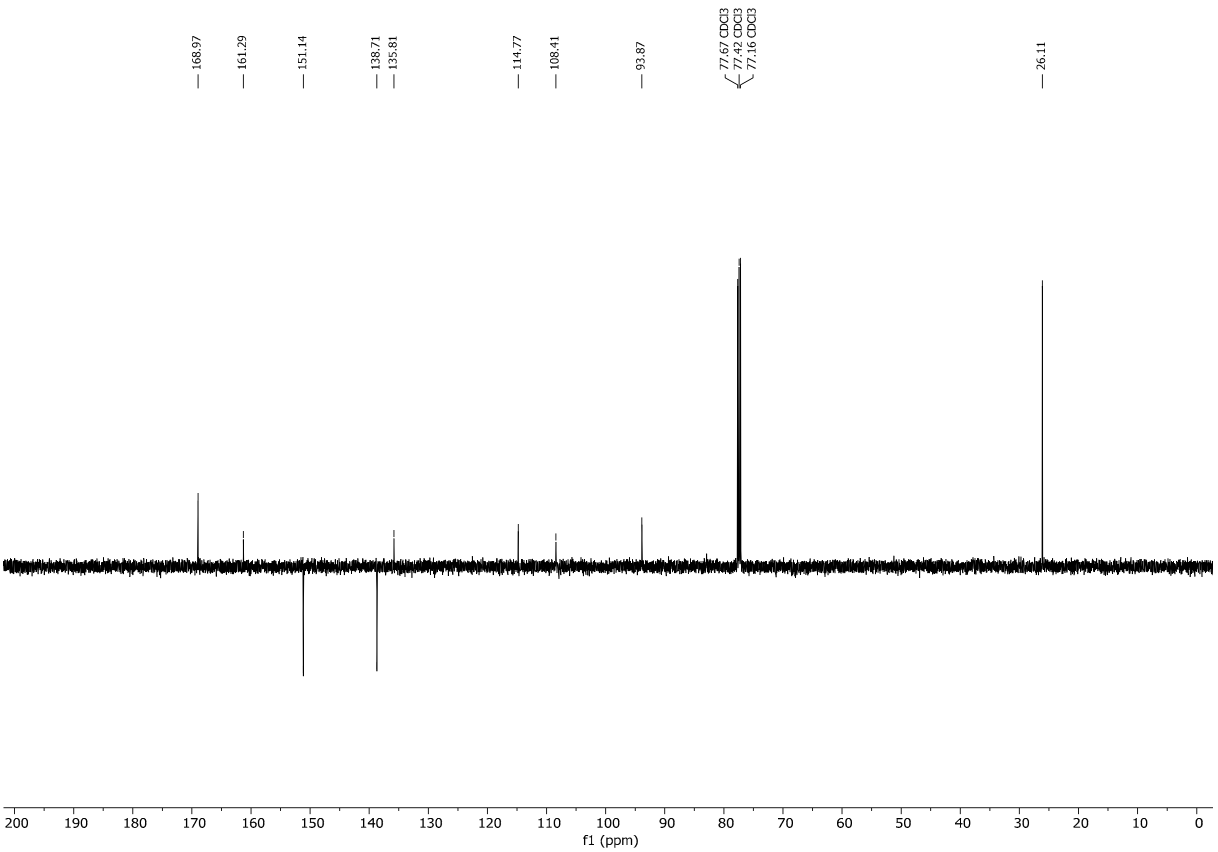

**TIBA-PSMA 617 (3)**

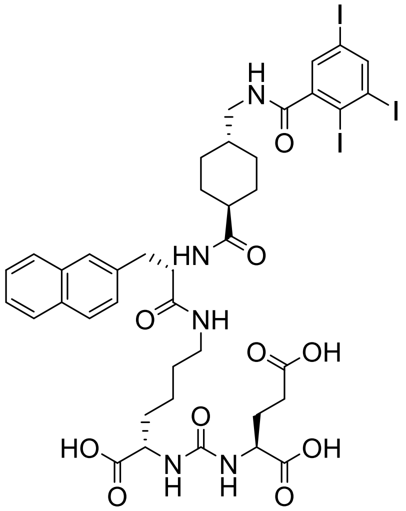

**1** (3.0 mg, 4.5 µmol, 1 equiv.) was dissolved in 2 mL dry DMF. After the addition of DIPEA (12.5 µL, 72.0 µmol, 16 equiv.), **2** (3.8 mg, 6.4 mmol, 1.4 equiv.) was added. The reaction was stirred under $N_2$ for 24 hours. After removing the solvent *in vacuo*, the residue was dissolved in $H_2O/CH_3CN$ with 0.1% $HCO_2H$ and purified by column chromatography on RP-18 silica gel ($H_2O/CH_3CN$ with 0.1% $HCO_2H$). The product was obtained as a colorless powder (1.3 mg, 1.14 µmol, 25%).

**HRMS** (ESI) *m/z* calculated for $C_{40}H_{46}I_3N_5O_{10}$: 1136.0306, found: 1136.0313 $[M-H]^+$.

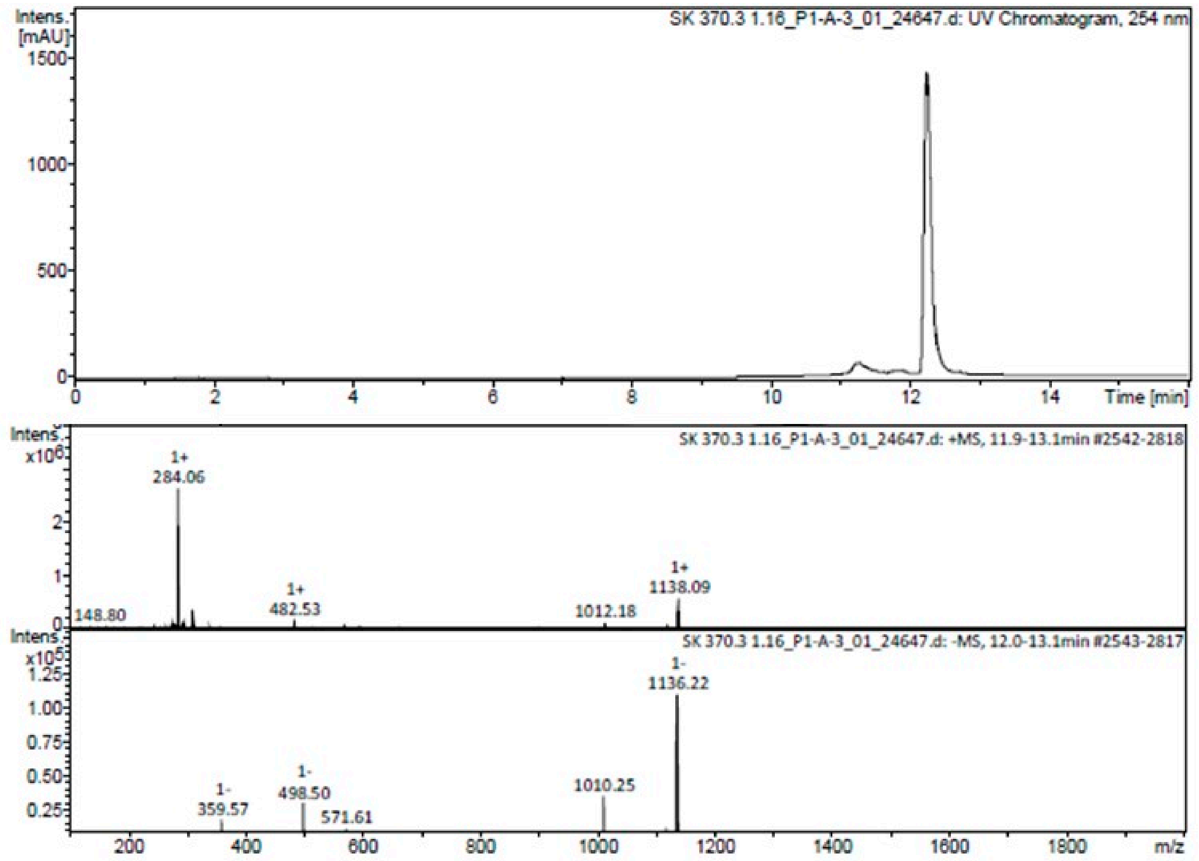


HPLC conditions: EC 150/2 Nucleodur C18 HTec, 5 µm column from Macherey & Nagel (4.6 × 100 mm) was used as stationary phase and the following gradient of MeCN in $H_2O$ with 0.1 % formic acid was used as mobile phase:

| Time | $H_2O$ | MeCN | Flow |
|---|---|---|---|
| 3 | 95 | 5 | 0.25 |
| 14 | 5 | 95 | 0.25 |
| 16 | 95 | 95 | 0.25 |

**Boc-AHX-IPA-NHS (5)**

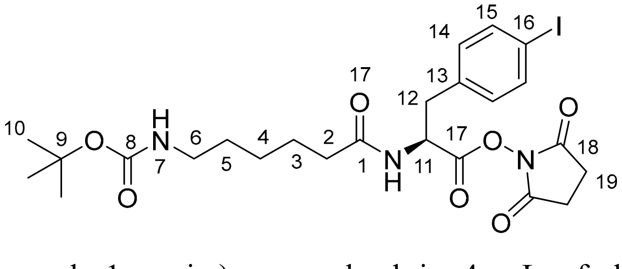

**VIII** (212.8 mg, 42 mmol, 1 equiv.) was solved in 4 mL of dry $CH_2Cl_2$ and *N*-(3-dimethylaminopropyl)-*N*'-ethyl-carbodiimide hydrochloride (EDC) (97.1 mg, 50.7 mmol, 1.2 equiv.), and *N*-hydroxysuccinimide (58.3 mg, 50.7 mmol, 1.2 equiv.) were added. The mixture was stirred for 20 h at room temperature under $N_2$ atmosphere and washed with water (3 x 4 mL). The combined organic phases were dried over $Na_2SO_4$ and the solvent was removed under reduced pressure. The product obtained as a white powder (190.0 mg, 31.6 mmol, 75%).

$^{1}$**H NMR** (600 MHz, MeOD): $\delta$ [ppm] = 7.63 (d, $^{3}J$ = 8.3 Hz, 2H, H-15), 7.00 (d, $^{3}J$ = 8.3 Hz, 2H, H-14), 4.67 (dd, $^{3}J$ = 9.5, 5.4 Hz, 1H, H-11), 3.16 – 3.11 (m, 1H, Ha-12), 3.00 (t, $^{3}J$ = 7.1 Hz, 2H, H-6), 2.92 – 2.86 (m, 1H, Hb-12), 2.68 – 2.67 (m, 4H, H-19), 2.16 – 2.13 (m, 2H, H-2), 1.51 (p, $^{3}J$ = 7,4 Hz, 2H, H-5), 1.45 – 1.40 (m, 11H, H-3, H-10), 1.24 – 1.19 (m, 2H, H-4).
$^{13}$**C NMR** (151 MHz, MeOD): δ 174.6 (C-1), 173.5 (C-18), 172.0 (C-17), 157.1 (C-8), 137.3 (C-15), 136.8 (C-13), 131.0 (C-14), 91.3 (C-16), 78.4 (C-9), 53.3 (C-11), 39.8 (C-6), 36.4 (C-12), 35.1 (C-2), 29.2 (C-3), 27.4 (C-10), 25.8 (C-4), 25.1 (C-5), 24.9 (C-19).
**HRMS** (ESI) *m/z* calculated for $C_{24}H_{32}IN_3O_7$: 624.1177, found: 624.1165 [M+Na]$^{+}$
**TLC:** $R_f$ = 0.30 (*n*-pentane/EtOAc 1:1, v/v, ninhydrin).

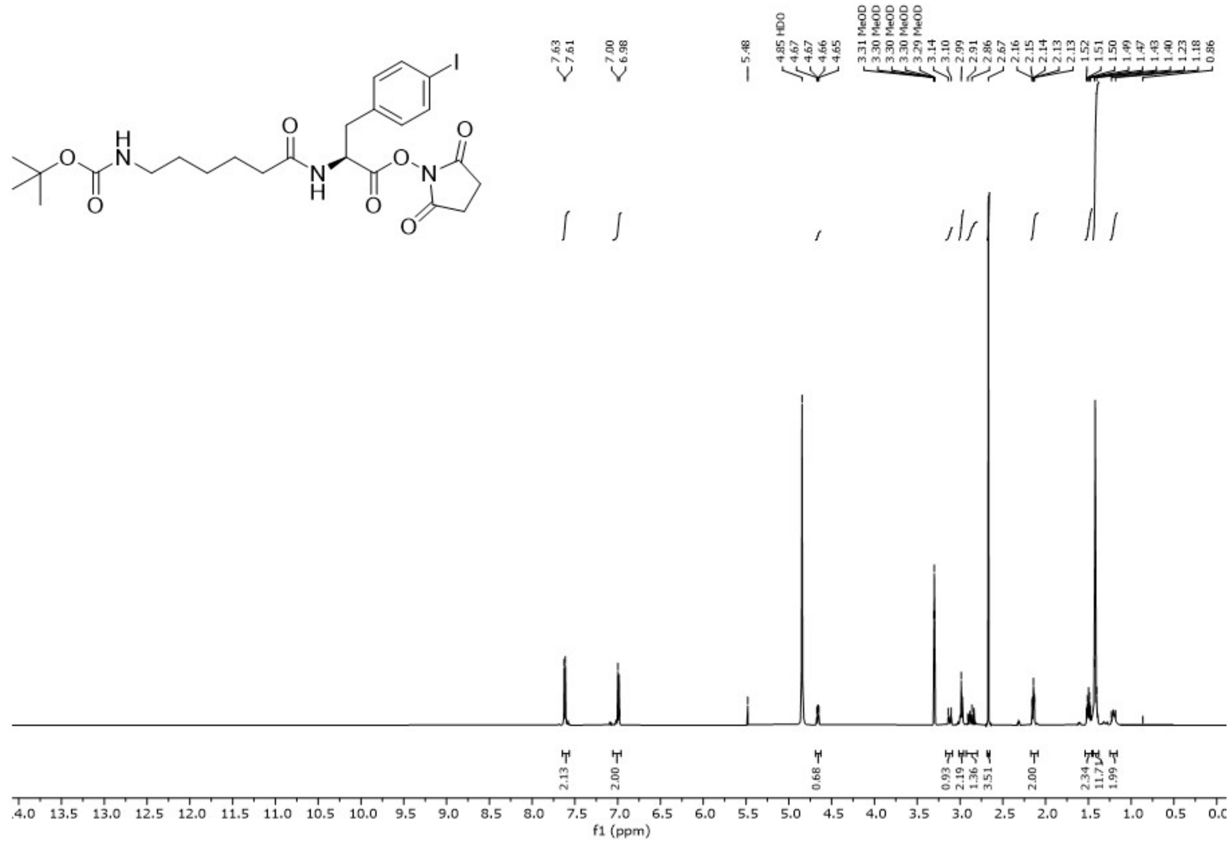

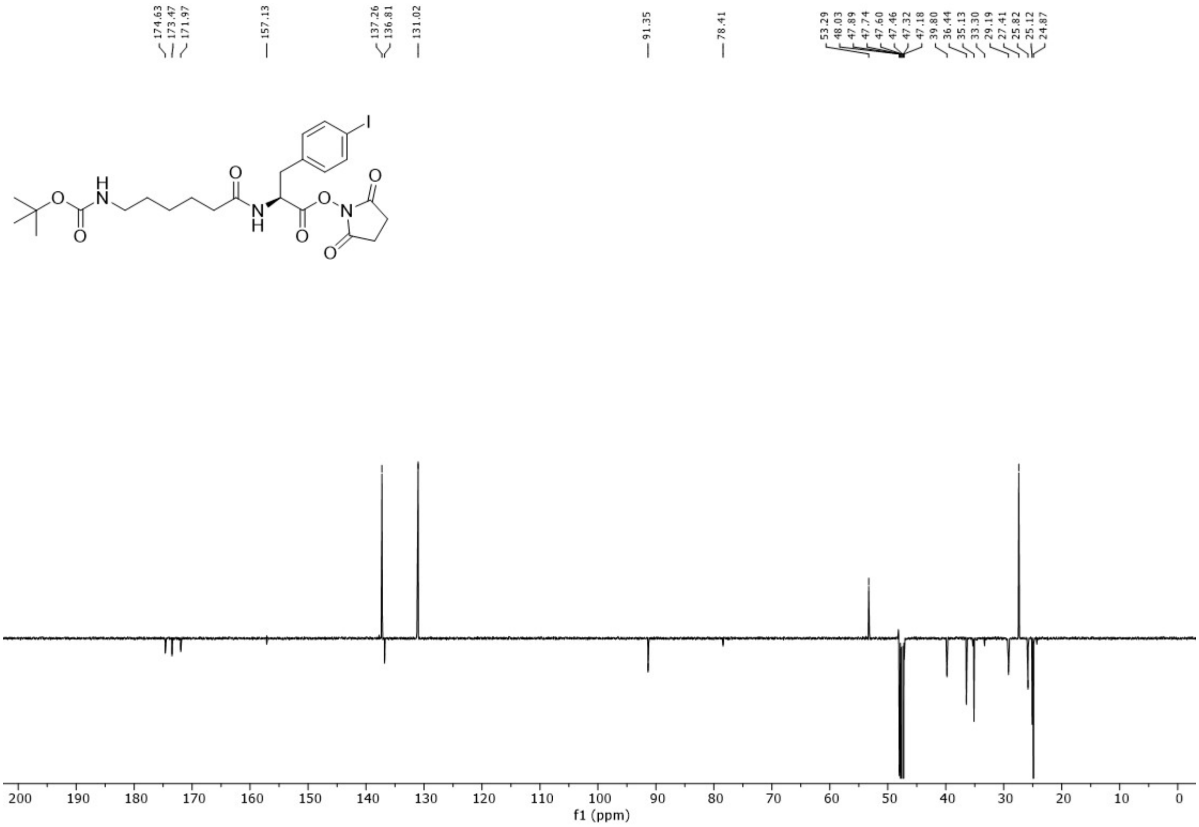


**Urea (Boc-6)**

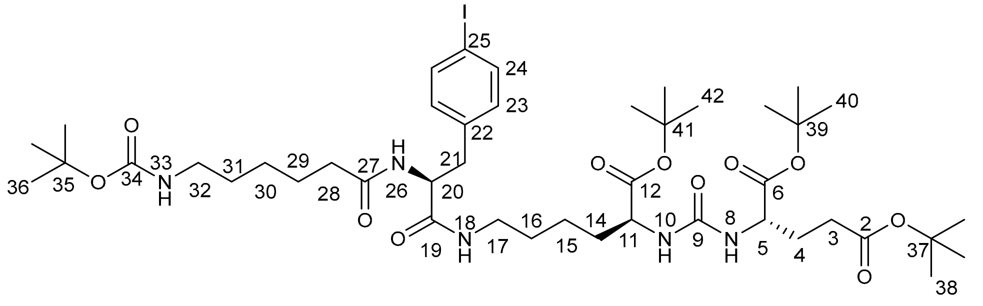

**4a**[3] (70 mg, 143 µmol) was dissolved in 15 mL dry DMF. After the addition of DIPEA (50 µL, 300 µmol), **5** (96 mg, 160 µmol) was added. The reaction was stirred under $N_2$ for 16 hours. After removing the solvent *in vacuo*, the residue was dissolved in $H_2O/CH_3CN$ with 0.1% $HCO_2H$ and purified by column chromatography on RP-18 silica gel ($H_2O/CH_3CN$ with 0.1% $HCO_2H$). The product was obtained as a colorless powder (82 mg, 102 µmol, 60%).

**$^1$H NMR** (400 MHz, DMSO-*d*$_6$) δ = 7.98-7,92 (m, 2H, H-18), 7.60 (d, *J* = 8.2 Hz, 2H, H-24), 7.03 (d, *J* = 8.1 Hz, 2H, H-23), 6.70 (t, *J* = 5.7 Hz, 1H, H-33), 6.30 (d, *J* = 8.4 Hz, 1H, H-8), 6.25 (d, *J* = 8.2 Hz, 1H, H-10), 4.44 (td, *J* = 9.0, 5.1 Hz, 1H, H-20), 4.05 (td, *J* = 8.4, 5.1 Hz, 1H, H-10), 3.96 (td, *J* = 8.0, 5.3 Hz, 1H, H-5), 3.08-2.95 (m, 2H, H-32), 2.90-2.83 (m, 3H, H-17, H-21a), 2.76-2.65 (m, 1H, H-21b), 2.30-2.17 (m, 2H, H-3), 2.02 (t, *J* = 7.4 Hz, 2H, H-28),

1.92-1.83 (m, 1H, H-4), 1.72-1.45 (m, 5H, H-4, H-16, H-14), 1.41 - 1.36 (m, 37H, H-tert), 1.34-1.21 (m, 7H, H-29, H30, H-31), 1.12-1.04 (m, 2H, H-15).

**$^{13}$C NMR** (101 MHz, DMSO) δ = 172.7 (C-2), 172.4 (C-12), 172.3 (C-6), 171.9 (C-19), 171.2 (C-27), 157.6 (C-9), 156.0 (C-34), 138.3 (C-22), 137.2 (C-24), 132.1 (C-23), 92.4 (C-25), 81.0 (C-41), 80.7 (C-39), 80.2 (C-37), 77.7 (C-35), 54.1 (C-20), 53.5 (C-11), 52.6 (C-5), 38.8 (C-32), 38.7 (C-17), 38.0 (C-21), 35.6 (C-28), 32.1 (C-3), 31.3 (C-14), 29.7 (C-31), 29.1 (C-4), 28.7 (C-42), 28.2 (C-40), 28.2 (C-36), 28.1 (C-38), 26.3 (C-16), 25.4 (C-30), 22.9 (C-15), 22.9 (C-29).

**HRMS (ESI):** *m/z* ber. für $C_{44}H_{72}IN_5O_{11}Na$: 996.4165, gef.: 996.4186 $[M+Na]^+$.

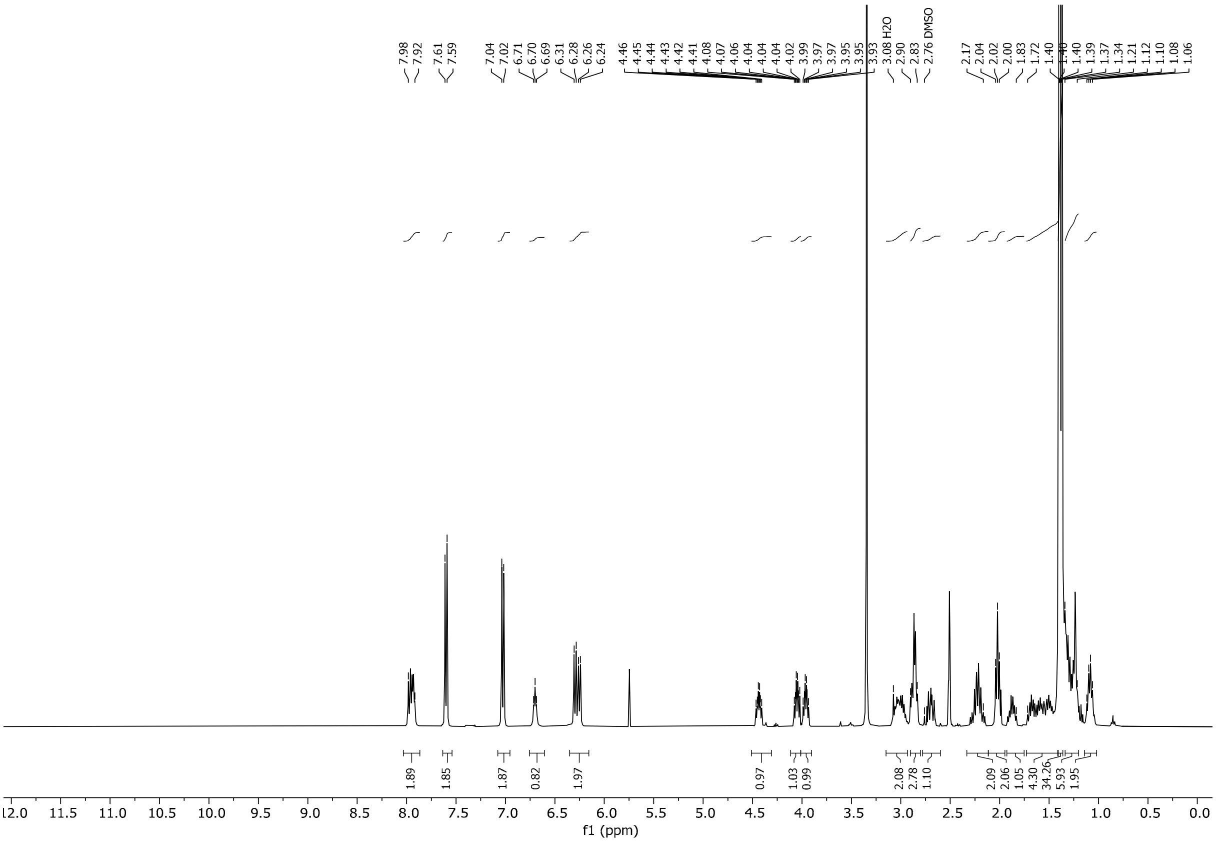

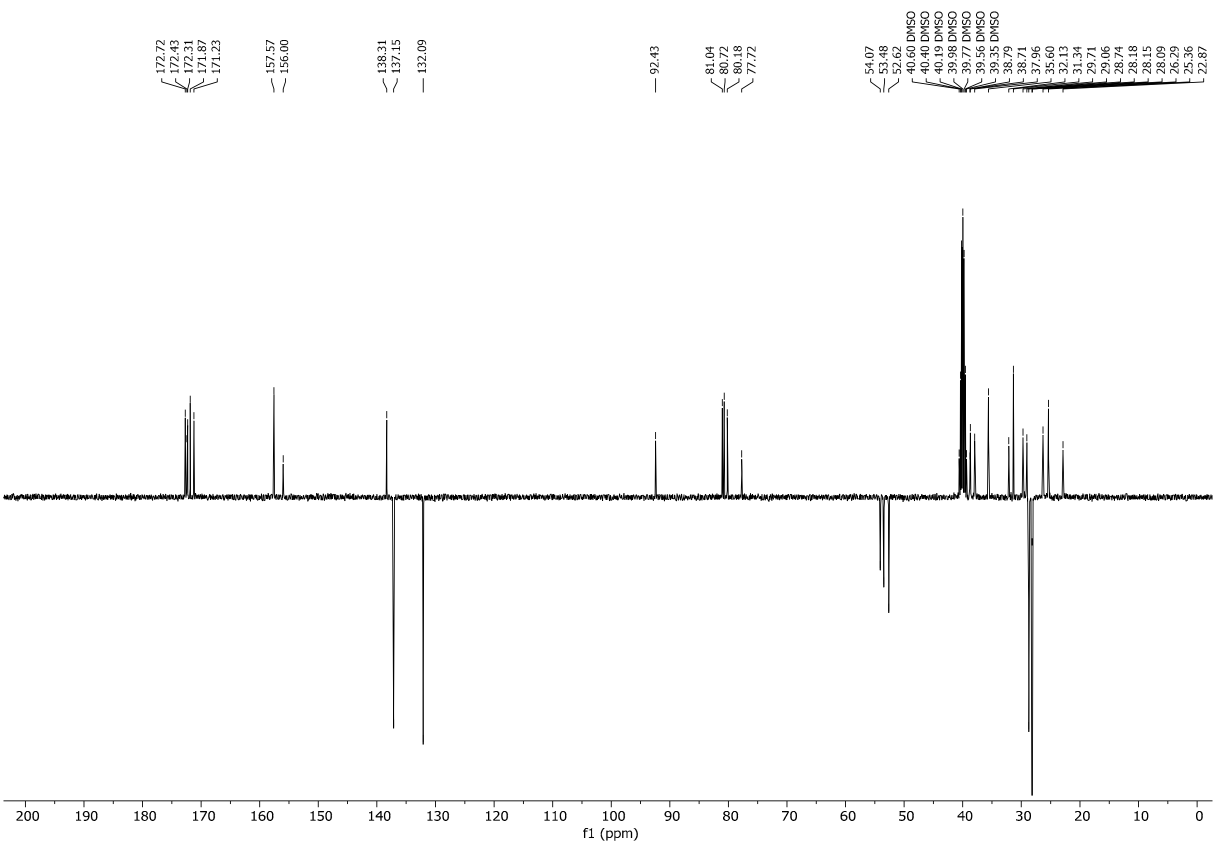


**Urea (6)**

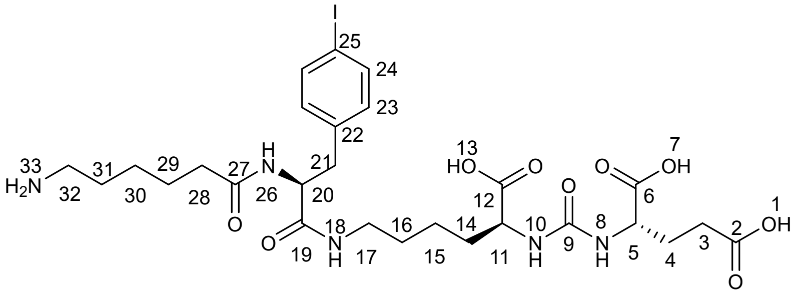

**Boc-6** (81 mg, 101 µmol) was dissolved in 3 mL $CH_2Cl_2$/TFA (1:1). The reaction solution was stirred at room temperature for 3 hours. Subsequently, the solvent was removed with $N_2$ and the residue was dissolved in $CH_2Cl_2$. The solution was neutralized by the addition of $NEt_3$. After removing the solvent *in vacuo*, the residue was dissolved in $H_2O/CH_3CN$ and purified by column chromatography on RP-18 silica gel ($H_2O/CH_3CN$ with 0.1% $HCO_2H$). The compound was obtained (52 mg, 74 µmol, 73%) as a colorless powder.

**$^1$H NMR** (300 MHz, DMSO-*d*$_6$) δ = 12.46 (s, 1H, $H_{Carbonsäure}$), 8.04 - 7.97 (m, 2H, H-26, H-18), 7.85 - 7.71 (m, 2H, H-33), 7.61 (d, J = 8.2 Hz, 2H, H-24), 7.04 (d, J = 8.3 Hz, 2H, H-23), 6.34 (t, J = 9.4 Hz, 2H, H-10, H-8), 4.47 - 4.39 (m, 1H, H-20), 4.14 - 4.00 (m, 2H, H-11, H-5), 3.01 (p, *J* = 6.8 Hz, 1H, H-32), 2.89 (dd, J = 13.6, 4.9 Hz, 1H, H-21), 2.77 - 2.65 (m, 3H, H-17, H-

21), 2.28 - 2.21 (m, 2H, H-3), 2.04 (t, $J$ = 7.4 Hz, 2H, H-28), 1.96 - 1.87 (m, 1H, H-4), 1.77 - 1.61 (m, 2H, H-4, H-14) 1.54 - 1.11 (m, 11H, H-14, H-16, H-30, H-31, H-29, H-15).

**$^{13}$C NMR** (101 MHz, DMSO-$d_6$) $\delta$ = 174.6 (C-2), 174.2 (C-12), 173.8 (C-6), 171.9 (C-19), 170.9 (C-27), 157.4 (C-9), 138.0 (C-22), 136.7 (C-24), 131.7 (C-23), 92.0 (C-25), 53.7 (C-20), 52.3 (C-11), 51.7 (C-5), 38.8 (C-32), 38.4 (C-17), 37.5 (C-21), 34.9 (C-28), 31.8 (C-3), 29.9 (C-14), 28.7 (C-31), 27.6 (C-4), 26.8 (C-16), 25.3 (C-30), 24.6 (C-29), 22.6 (C-15).

**HRMS (ESI):** *m/z* calc. for $C_{27}H_{40}IN_5O_9$: 706.1944, found.: 706.1910 $[M+H]^+$.

**IR:** $\tilde{\nu}$max (cm$^{-1}$) = 3305.99, 2931.80, 2862.36, 1705.07, 1635.64, 1543.05, 1400.32, 1203.58, 1006.84

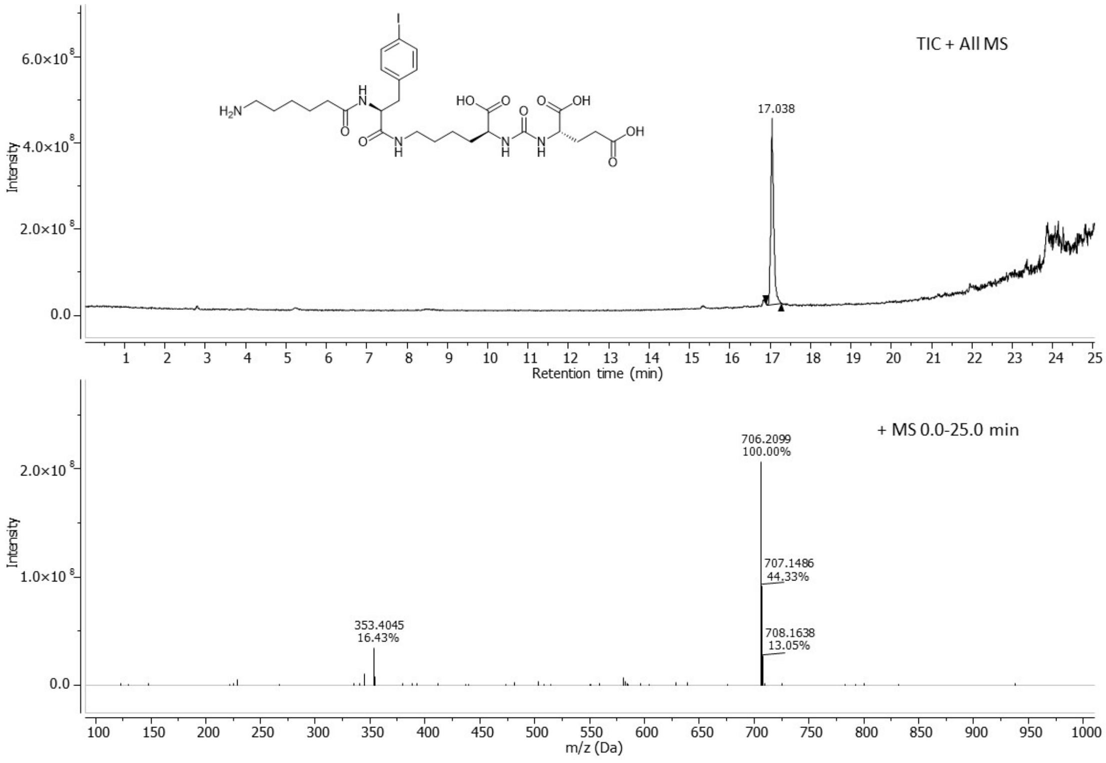


HPLC conditions: EC 150/2 Nucleodur C18 HTec, 5 µm column from Macherey & Nagel (4.6 × 100 mm) was used as stationary phase and the following gradient of MeCN in $H_2O$ with 0.1 % formic acid was used as mobile phase:

| Time | $H_2O$ | MeCN | Flow |
|---|---|---|---|
| 3 | 95 | 5 | 0.25 |
| 20 | 5 | 95 | 0.25 |
| 21 | 95 | 5 | 0.25 |
| 25 | 95 | 5 | 0.25 |

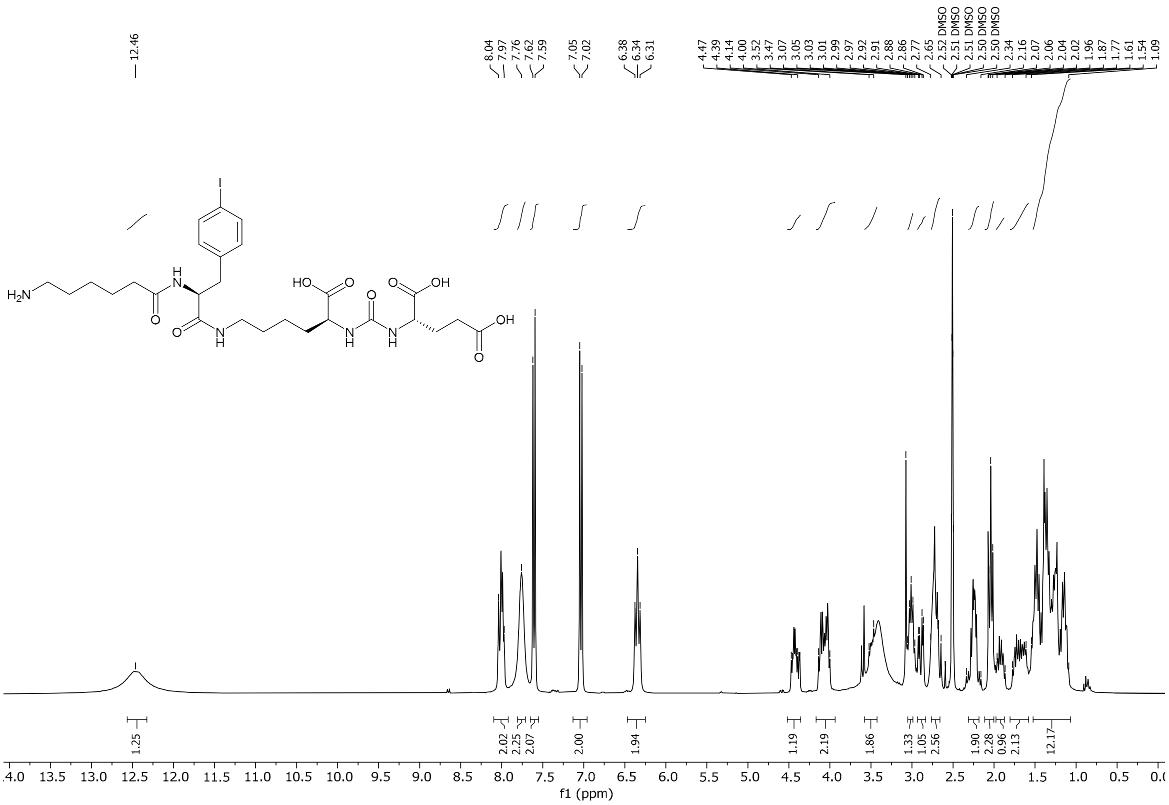

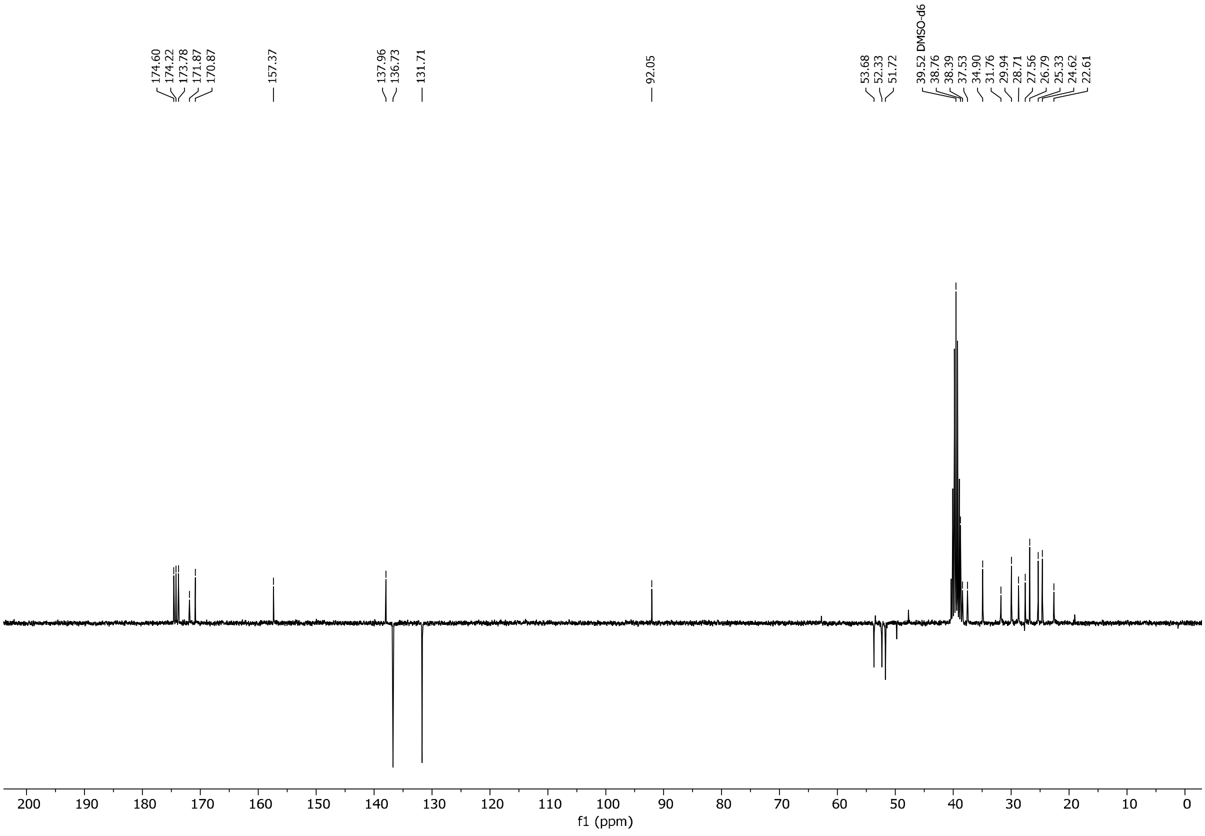

**Urea (7)**

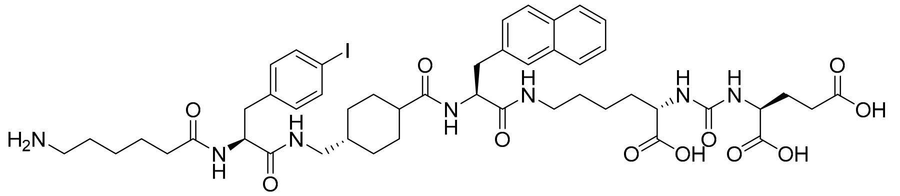

**1d** (5 mg, 44 µmol) was dissolved in 2 mL $CH_2Cl_2$/TFA (1:1). The reaction solution was stirred for 3 h at room temperature. The solvent was removed *via* $N_2$, the residue was dissolved in $CH_2Cl_2$ and the solution was neutralized by adding $NEt_3$. After removal of the solvent *in vacuo*, the residue was dissolved in $H_2O/CH_3CN$ and purified by column chromatography on RP-18 silica gel ($H_2O/CH_3CN$ with 0.1% $HCO_2H$). A colorless powder of 4 mg (44 µmol, quant.) was obtained.

**HRMS (ESI):** *m/z c*alculated for $C_{48}H_{64}IN_7O_{11}$: 1042.3781, found: 1042.3765 $[M+H]^+$
**IR** (ATR): $\tilde{\nu}$ [cm$^{-1}$] = 3278, 2927, 2858, 1705, 1627, 1543, 1485, 1442, 1392, 1211, 1126, 1060, 1006, 856, 632.
**DC:** $R_f$ = 0.50 ($CH_2Cl_2$/MeOH 5:1, v/v, ninhydrin).
**Smp.**: 160°C

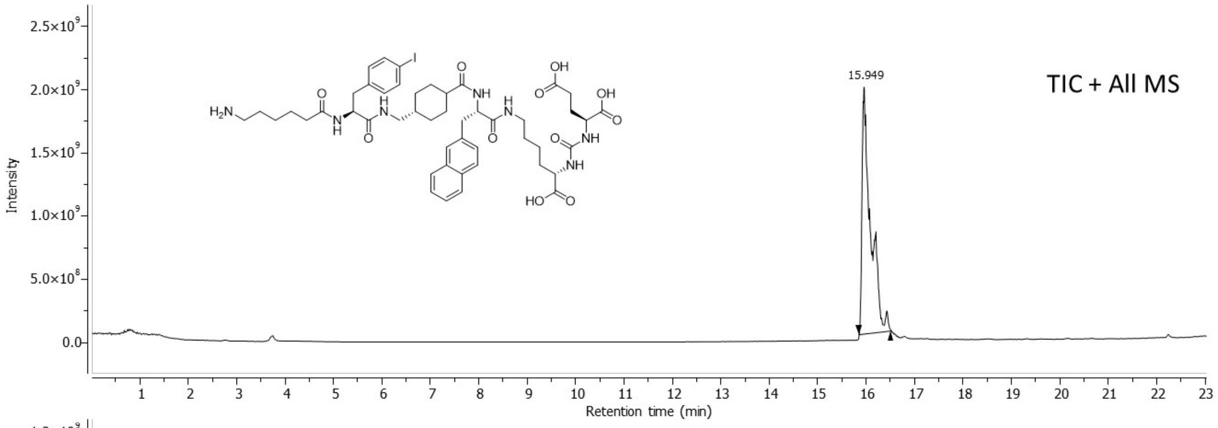


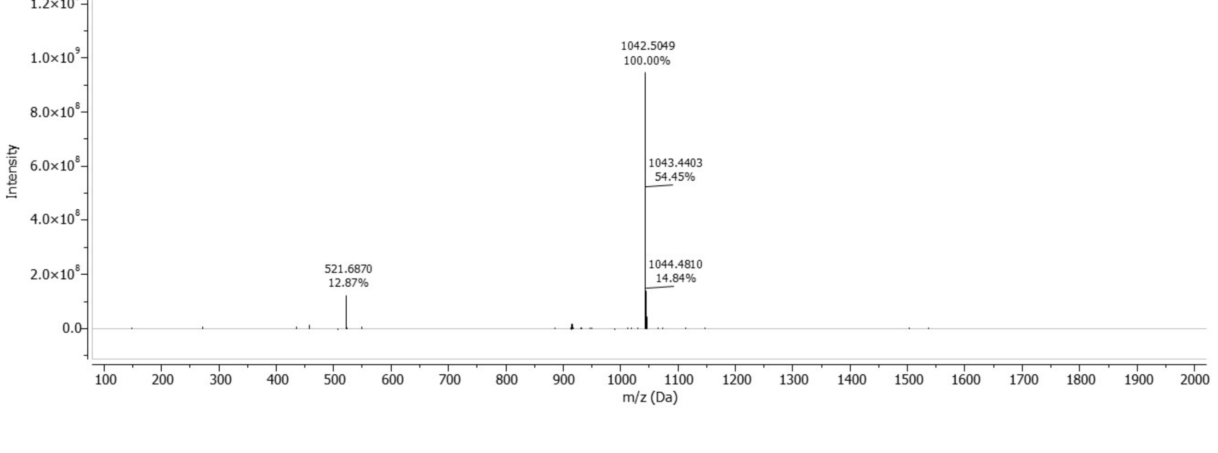

HPLC conditions: EC 150/2 Nucleodur C18 HTec, 5 µm column from Macherey & Nagel (4.6 × 100 mm) was used as stationary phase and the following gradient of MeCN in $H_2O$ with 0.1 % formic acid was used as mobile phase:

| Time | $H_2O$ | MeCN | Flow |
|---|---|---|---|
| 3 | 95 | 5 | 0.25 |
| 20 | 5 | 95 | 0.25 |
| 21 | 95 | 5 | 0.25 |
| 23 | 95 | 5 | 0.25 |

**Urea (1a)**

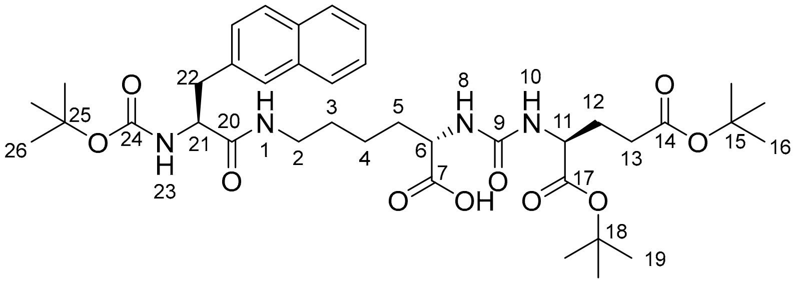


**4**[3] (Lys-Urea-Glu) (440 mg, 1.00 mmol, 1 equiv.) was dissolved in 20 mL of $CH_2Cl_2$. After the addition of DIPEA (0.44 mL, 2.5 mmol, 2.5 equiv.), **III** (504 mg, 1.20 mmol, 1.2 equiv.) was added. The reaction solution was stirred for 16 h. After removal of the solvent *in vacuo*, the residue was dissolved in $H_2O/CH_3CN$ with 0.1% $HCO_2H$ and purified by column chromatography on RP-18 silica gel ($H_2O/CH_3CN$ with 0.1% $HCO_2H$). The product was obtained as a white powder (651 mg, 0.89 mmol, 89%).

**$^1$H NMR** (300 MHz, $CD_3OD$): δ 7.82 – 7.72 (m, 3H, $H_{naphtyl}$), 7.68 – 7.67 (m, 1H, $H_{naphtyl}$), 7.48 – 7.37 (m, 3H, $H_{naphtyl}$), 4.36 (t, $^3J$ = 7.5 Hz, 1H, 21-H), 4.22-4.15 (m, 2H, 6-H, 11-H), 3.24 – 3.98 (m, 4H, 2-H, 22-H), 2.34-2.28 (m, 2H, 13-H), 2.09 – 1.97 (m, 1H, 12-Ha), 1.87 – 1.76 (m, 1H, 12-Hb), 1.72 – 1.65 (m, 1H, 5-Ha), 1.59-1.51 (m, 1H, 5-Hb), 1.46 (s, 9H, 19-H), 1.44 (s, 9H, 16-H), 1.32 (s, 9H, 26-H), 1.27-1.21 (m, 4H, 3-H, 4-H).

**$^{13}$C NMR** (75 MHz, $CD_3OD$): δ 173.9 (C-7), 172.4 (C-14), 157.7.6 (C-17), 156.5 (C-20), 156.0 (C-24), 134.7 ($C_{naphtyl}$), 133.3 ($C_{naphtyl}$), 132.1 ($C_{naphtyl}$), 128.02 ($C_{naphtyl}$), 127.83 ($C_{naphtyl}$), 127.54 ($C_{naphtyl}$), 127.33 ($C_{naphtyl}$), 126.89 ($C_{naphtyl}$), 125.69 ($C_{naphtyl}$), 125.24 ($C_{naphtyl}$), 82.1 (C-15), 80.8 (C-18), 80,1 (C-25), 55.84 (C-21), 52.97 (C-6), 52.55 (C-11), 39.7 (C-2), 39.0 (C-22), 32.2 (C-5), 31.4 (C-13), 29.3 (C-12), 28.29 (C-26), 28.0 (C-19), 27.94 (C-16), 23.8 (C-3), 22.8 (C-4).

**HRMS** (ESI) *m/z* calculated for $C_{36}H_{56}IN_4O_{10}$: 729.4075, found: 729.4041 $[M+H]^+$.

**DC:** $R_f$ = 0.50 ($CH_2Cl_2$/MeOH 5:1, v/v, ninhydrin).

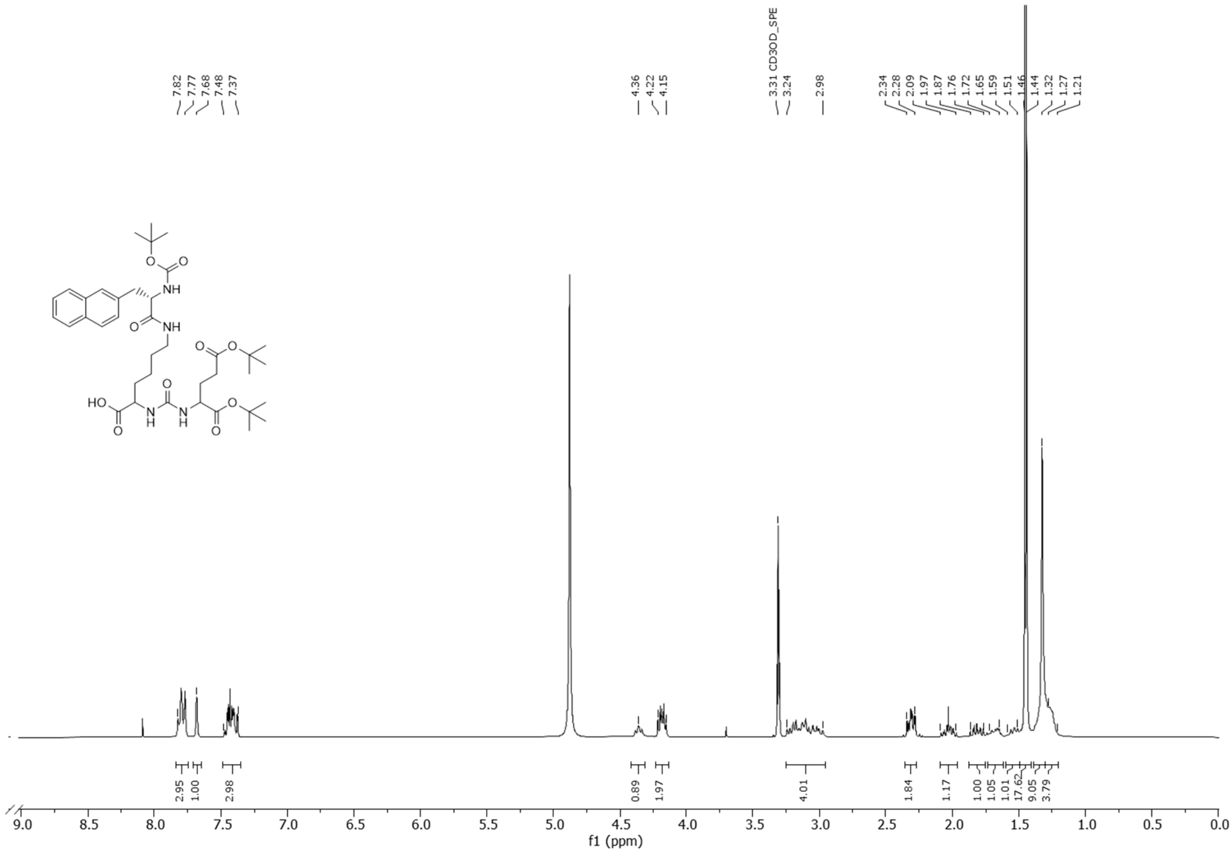
7.82
7.77
7.68
7.48
7.37
4.36
4.22
4.15
3.31 CD3OD_SPE
3.24
2.98
2.34
2.28
2.09
1.97
1.87
1.76
1.72
1.65
1.59
1.51
1.46
1.44
1.32
1.27
1.21
2.95
1.00
2.98
0.89
1.97
4.01
1.84
1.17
1.00
1.05
1.01
17.62
9.05
3.79
f1 (ppm)

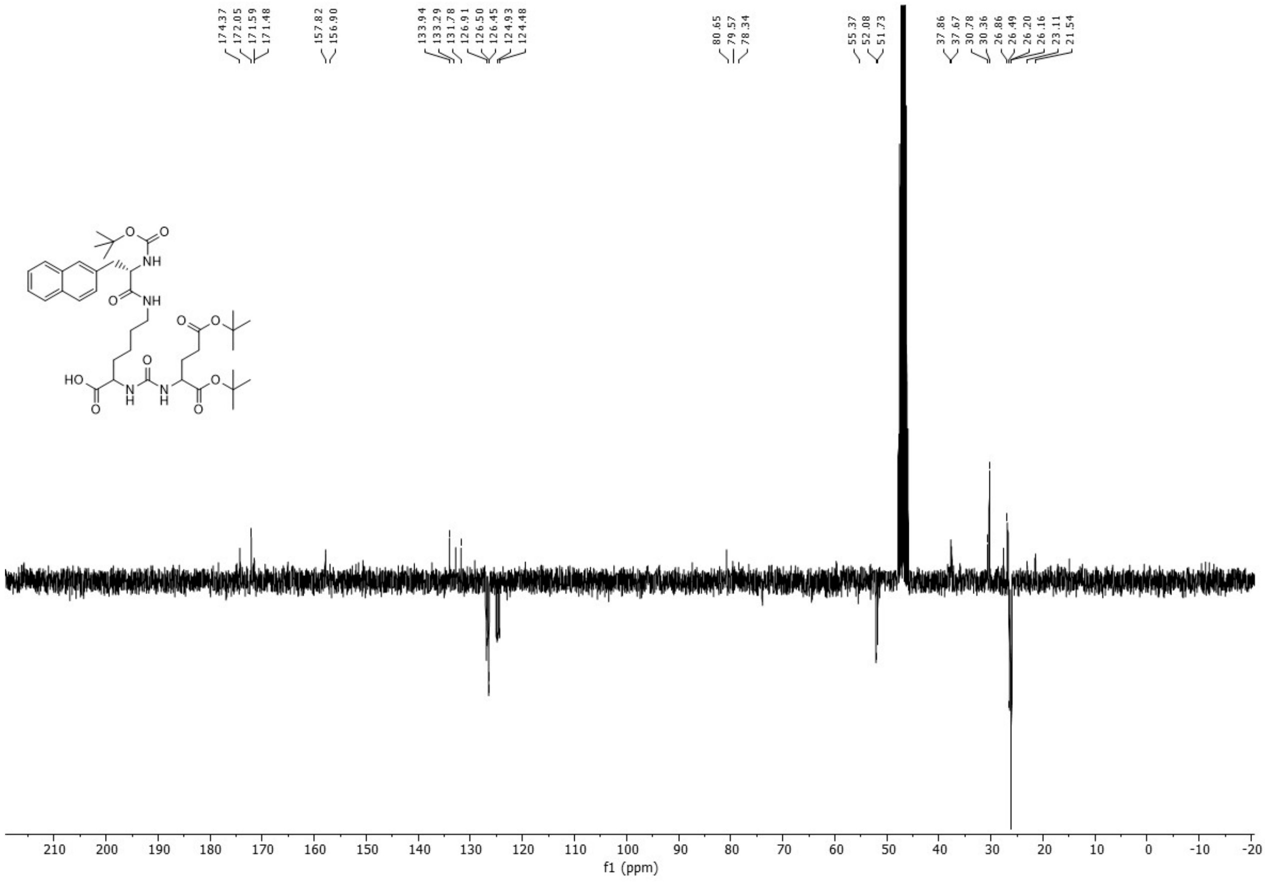
174.37
172.05
171.59
171.48
157.82
156.90
133.94
133.29
131.78
126.91
126.50
126.45
124.93
124.48
80.65
79.57
78.34
55.37
52.08
51.73
37.86
37.67
30.78
30.36
26.86
26.49
26.20
26.16
23.11
21.54
f1 (ppm)

**Urea (1b)**

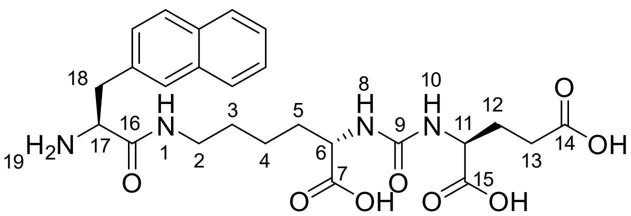


**1a** (631 mg, 0.87 mmol) was dissolved in 16 mL $CH_2Cl_2$/TFA (1:1). The reaction solution was stirred for 16 h at room temperature. The solvent was then removed *via* an $N_2$ stream. Residual solvent was removed under vacuum. 450 mg (0.87 mmol, quant.) of a white powder was obtained.

**$^1$H NMR** (300 MHz, Methanol-$d_4$) δ 7.94 – 7.82 (m, 3H, H-naphthyl), 7.77 – 7.74 (m, 1H, H-naphthyl), 7.51 – 7.48 (m, 2H, H-naphthyl), 7.42 – 7.39 (m, 1H, H-naphthyl), 4.37 – 4.25 (m, 1H, H-11), 4.20 – 4.04 (m, 2H, H-6, H-17), 3.28 – 3.15 (m, 2H, H-2), 3.09 – 2.97 (m, 1H, H-18a), 2.70 – 2.54 (m, 1H, H-18b), 2.47 – 2.32 (m, 2H, H-13), 2.23 – 1.98 (m, 1H, H-5a), 1.95 – 1.78 (m, 1H, H-5b), 1.73 – 1.40 (m, 2H, H-12), 1.36 – 1.14 (m, 4H, H-3, H-4).

**HRMS** (ESI) *m/z* calculated for $C_{25}H_{32}IN_4O_8$: 517.2298, found: 517.2298 $[M+H]^+$.

**DC:** $R_f$ = 0.50 ($CH_2Cl_2$/MeOH/1% AcOH 5:1, v/v, ninhydrin).

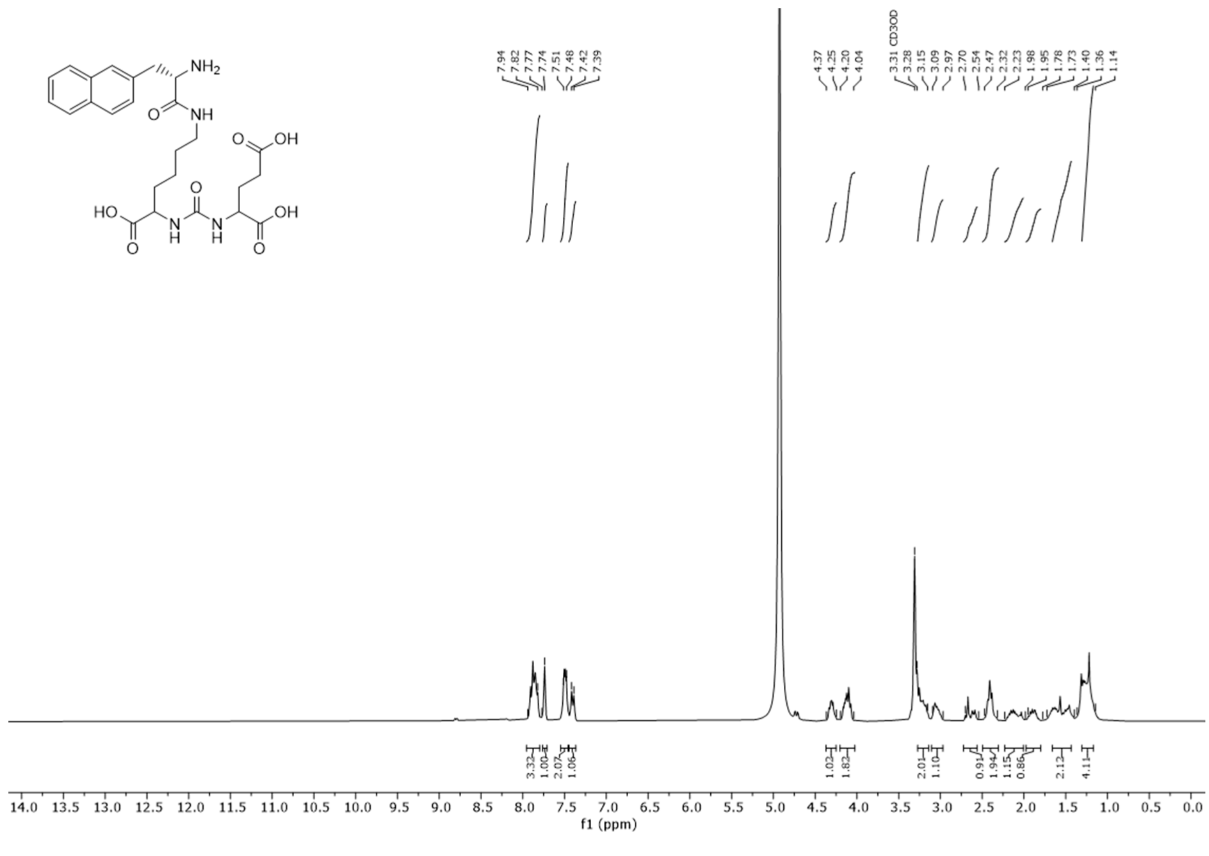

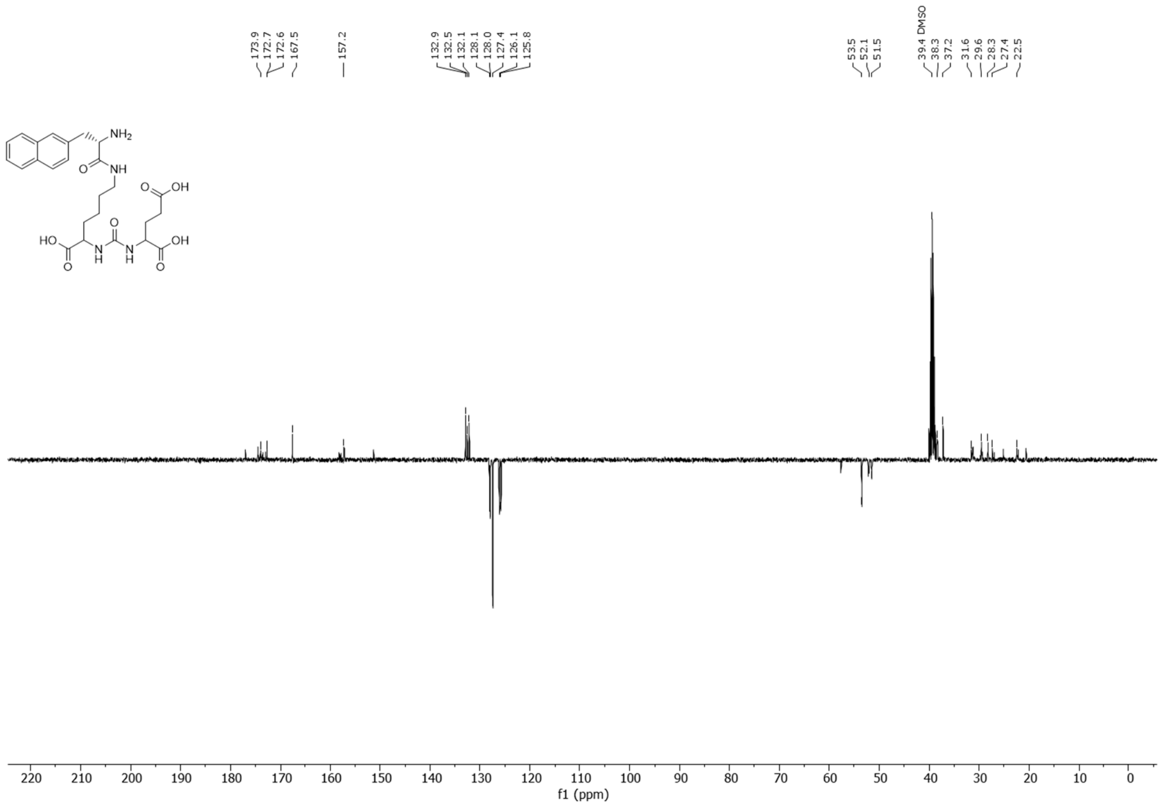

**Urea (1c)**

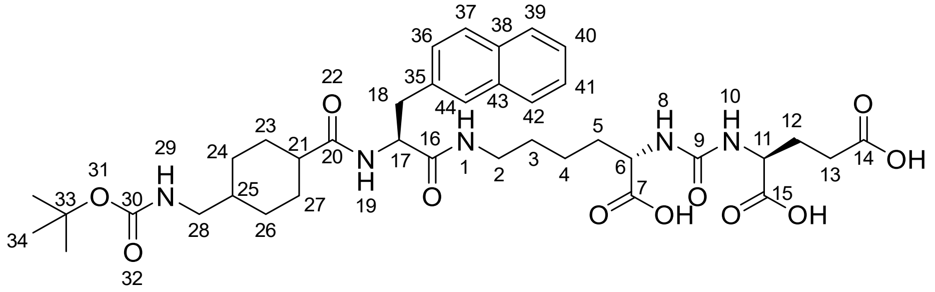

**1b** (38 mg, 74 µmol, 1 equiv.) was dissolved in 10 mL dry $CH_2Cl_2$. After the addition of DIPEA (130 µL, 0.74 mmol, 10 equiv.), **VI** (32 mg, 89 µmol, 1.2 equiv.) was added. The reaction solution was stirred for 16 h under $N_2$. After removal of the solvent *in vacuo*, the residue was dissolved in $H_2O/CH_3CN$ with 0.1% $HCO_2H$ and purified by column chromatography on RP-18 silica gel ($H_2O/CH_3CN$ with 0.1% $HCO_2H$). The product was obtained as a colorless powder (45 mg, 60 µmol, 81%)

**$^1$H NMR** (600 MHz, DMSO-*d*$_6$): δ 12,42 (br.s., 3H, H-carboxylic acid), 7.95 – 7.89 (m, 2H, 3-H, 19-H), 7.86 – 7.85 (m, 1H, 39-H), 7.81 – 7.77 (m, 2H, 42-H, 37-H), 7.68 (s, 1H, 44-H), 7.48 – 7.43 (m, 2H, 41-H, 40-H), 7.39 (dd, $^3J$ = 8.4, $^3J$ = 1.7 Hz, 1H, 36-H), 6.74 (t, $^3J$ = 6.0 Hz, 1H, 29-H), 6.32 (d, $^3J$ = 8.3 Hz, 1H, 10-H), 6.28 (d, $^3J$ = 8.3 Hz, 1H, 8-H), 4.52 (td, $^3J$ = 9.2 Hz, $^3J$

= 5.2 Hz, 1H, 17-H), 4.10 (td, $^3J$ = 8.3 Hz, $^3J$ =5.3 Hz, 1H, 11-H), 4.02 (td, $^3J$ = 8.3 Hz, $^3J$ = 5.1 Hz, 1H, 6-H), 3.10 (dd, $^2J$ = 13.7 Hz, $^3J$ = 5.2 Hz, 1H, 18-Ha), 3.07 – 3.03 (m, 1H, 2-Ha), 3.0 – 2.96 (m, 1H, 2-Hb), 2.92 (dd, $^2J$ = 13.7 Hz, $^3J$ = 9.2 Hz, 1H, 18-Hb), 2.72 (t, $^3J$ = 6.0 Hz, 2H, 28-H), 2.30 – 2.18 (m, 2H, 13-H), 2.05 (tt, $^3J$ = 12.1 Hz, $^3J$ = 3.3 Hz, 1H, 21-H), 1.94-1.87 (m, 1H, 12-$H_a$), 1.74-1.67 (m, 1H, 12-$H_b$), 1.66 – 1.61 (m, 1H, 24/26-Ha), 1.60 – 1.53 (m, 2H, 5-$H_a$, 23/27i-Ha), 1.50 – 1.43 (m, 2H, 5-$H_b$, 24/26-Ha), 1.36 (s, 9H, 34-H), 1.34 – 1.29 (m, 2H, 3-H), 1.27 – 1.15 (m, 4H, 4-H, 25-H, 23/27-$H_b$), 1.06 – 0.99 (m, 1H, 23/27-$H_b$), 0.82 – 0.74 (m, 2H, 24/26-$H_b$).

**$^{13}$C NMR** (151 MHz, DMSO-$d_6$): δ 175.0 (C-20), 174.6 (C-7), 174.2 (C-15), 173.8 (C-14), 171.0 (C-16), 157.3 (C-9), 155.8 (C-30), 135.8 (C-35), 132.9 (C-43), 131.8 (C-38), 127.9 (C-36), 127.5 (C-39, C-44), 127.3 (C-42), 126.0 (C-41), 125.4 (C-40), 77.3 (C-33), 53.7 (C-17), 52.3 (C-6), 51.7 (C-11), 46.1 (C-28), 43.7 (C-21), 38.4 (C-2), 37.4 (C-25), 31.7 (C-5), 30.0 (C-13), 29.6 (C-24/26), 29.4 (C-24/26), 28.8 (C-23/27), 28.7 (C-23/27), 28.4 (C-3), 28.3 (C-34), 27.6 (C-12), 22.6 (C-4).

**HRMS** (ESI) *m/z* calculated for $C_{38}H_{53}IN_5O_{11}$: 756.3820, found: 756.3819 $[M+H]^+$.

**DC:** $R_f$ = 0.30 ($CH_2Cl_2$/MeOH/1% AcOH 5:1, v/v, ninhydrin).

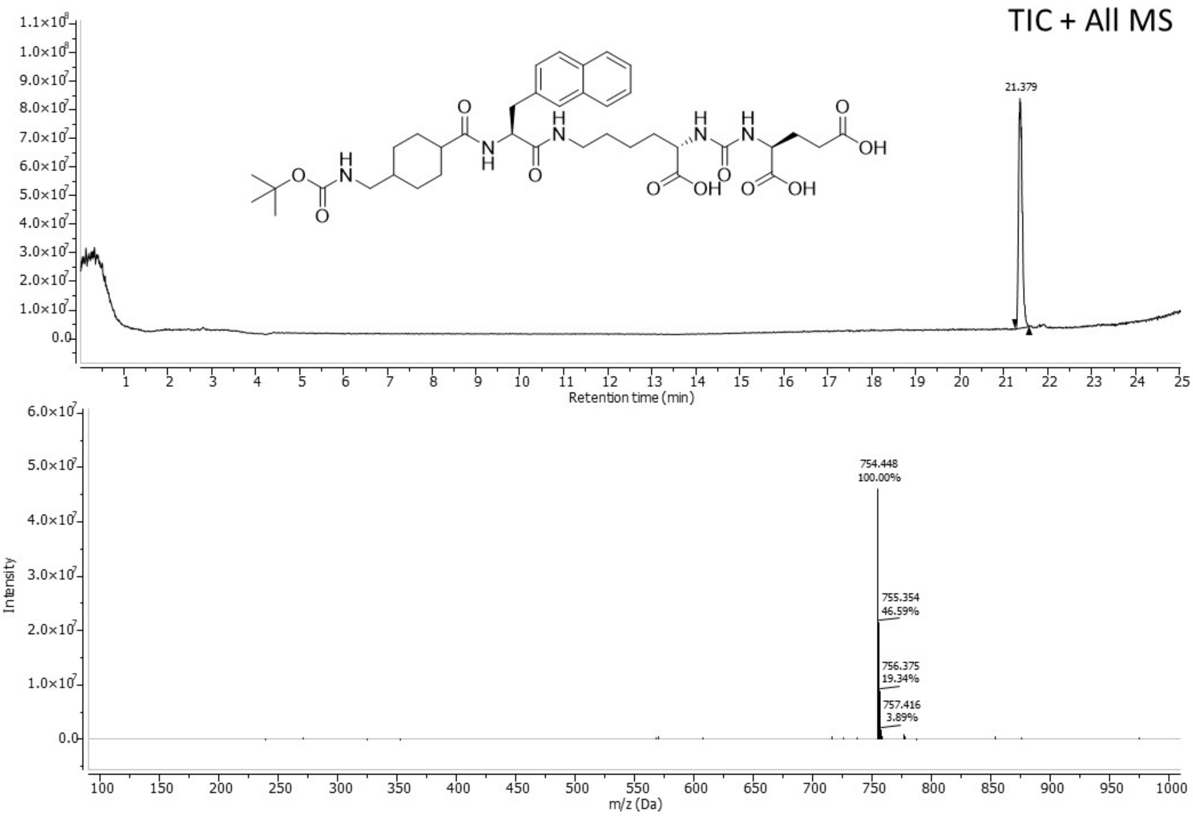

HPLC conditions: EC 150/2 Nucleodur C18 HTec, 5 µm column from Macherey & Nagel (4.6 × 100 mm) was used as stationary phase and the following gradient of MeCN in $H_2O$ with 0.1 % formic acid was used as mobile phase:

| Time | $H_2O$ | MeCN | Flow |
|---|---|---|---|
| 3 | 95 | 5 | 0.25 |
| 20 | 5 | 95 | 0.25 |
| 21 | 95 | 5 | 0.25 |
| 25 | 95 | 5 | 0.25 |

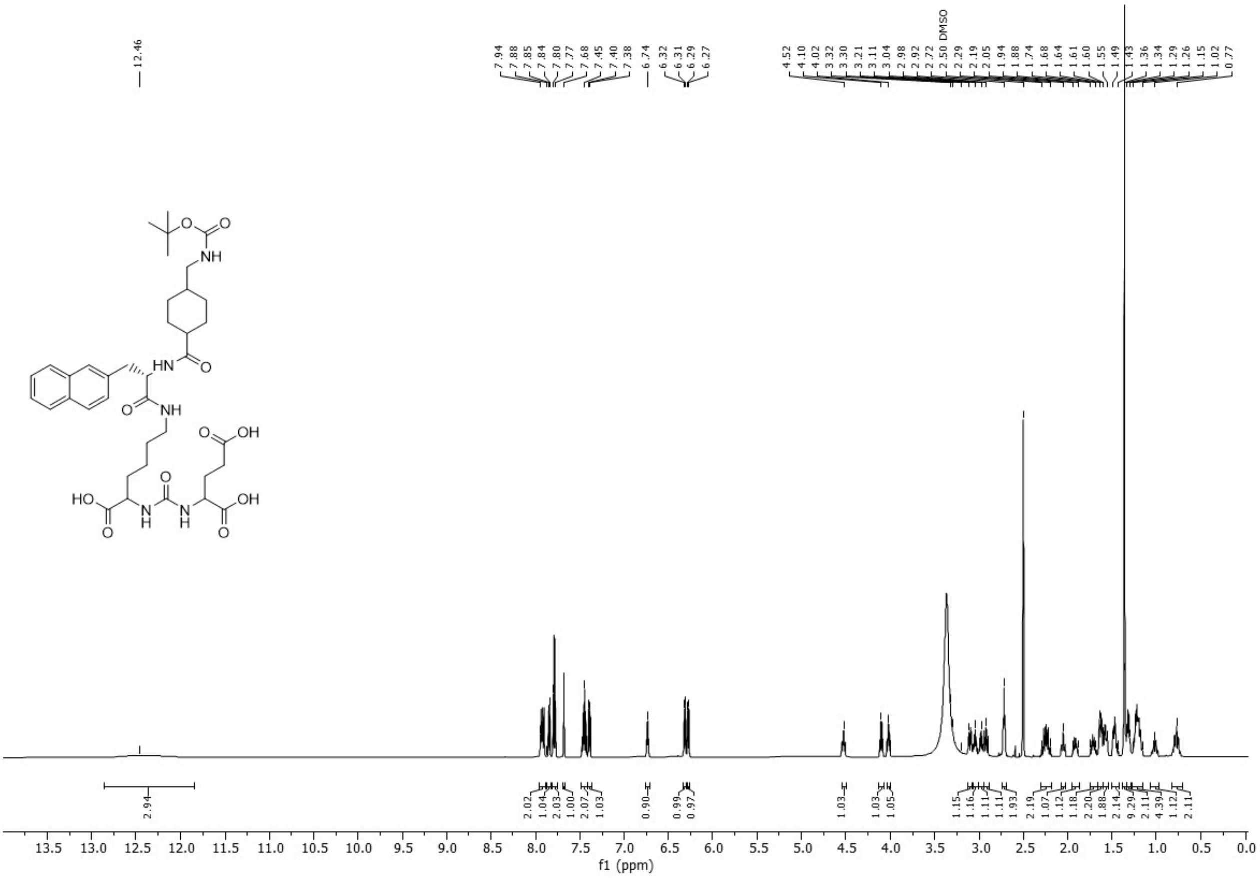

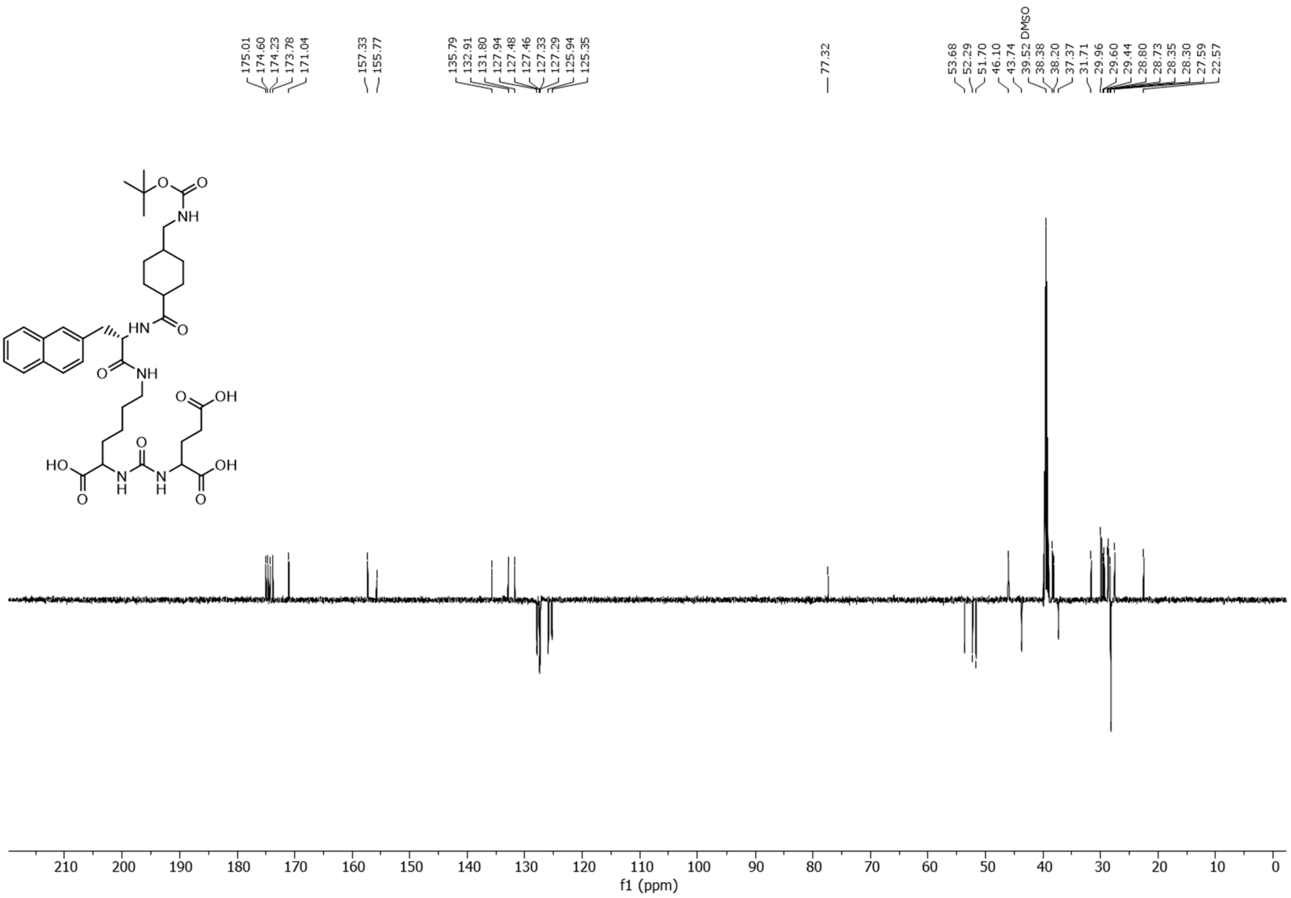


**Urea (1d)**

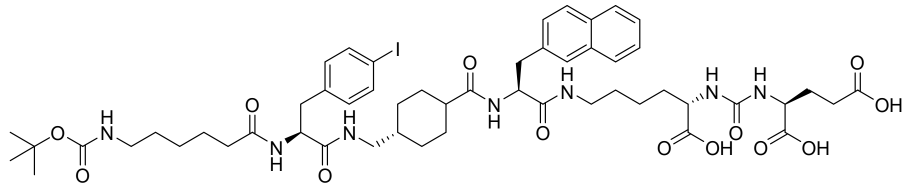

**1** (14 mg, 21 µmol, 1 equiv.) was dissolved in 2 mL dry DMF. After the addition of DIPEA (7 µL, 42 µmol, 2 equiv.), **5** (13 mg, 23 µmol, 1.1 equiv.) was added. The reaction was stirred for 16 h under $N_2$. After removal of the solvent *in vacuo*, the residue was dissolved in $H_2O/CH_3CN$ with 0.1% $HCO_2H$ and purified by column chromatography on RP-18 silica gel ($H_2O/CH_3CN$ with 0.1% $HCO_2H$). The product was obtained as a colorless powder (8 mg, 7 µmol, 32%).

**HRMS** (ESI) *m/z* calculated for $C_{53}H_{72}IN_7O_{13}$: 1142.4311, found: 1142.4316 $[M+H]^+$.
**DC:** $R_f$ = 0.46 ($CH_2Cl_2$/MeOH 5:1, v/v, ninhydrin).

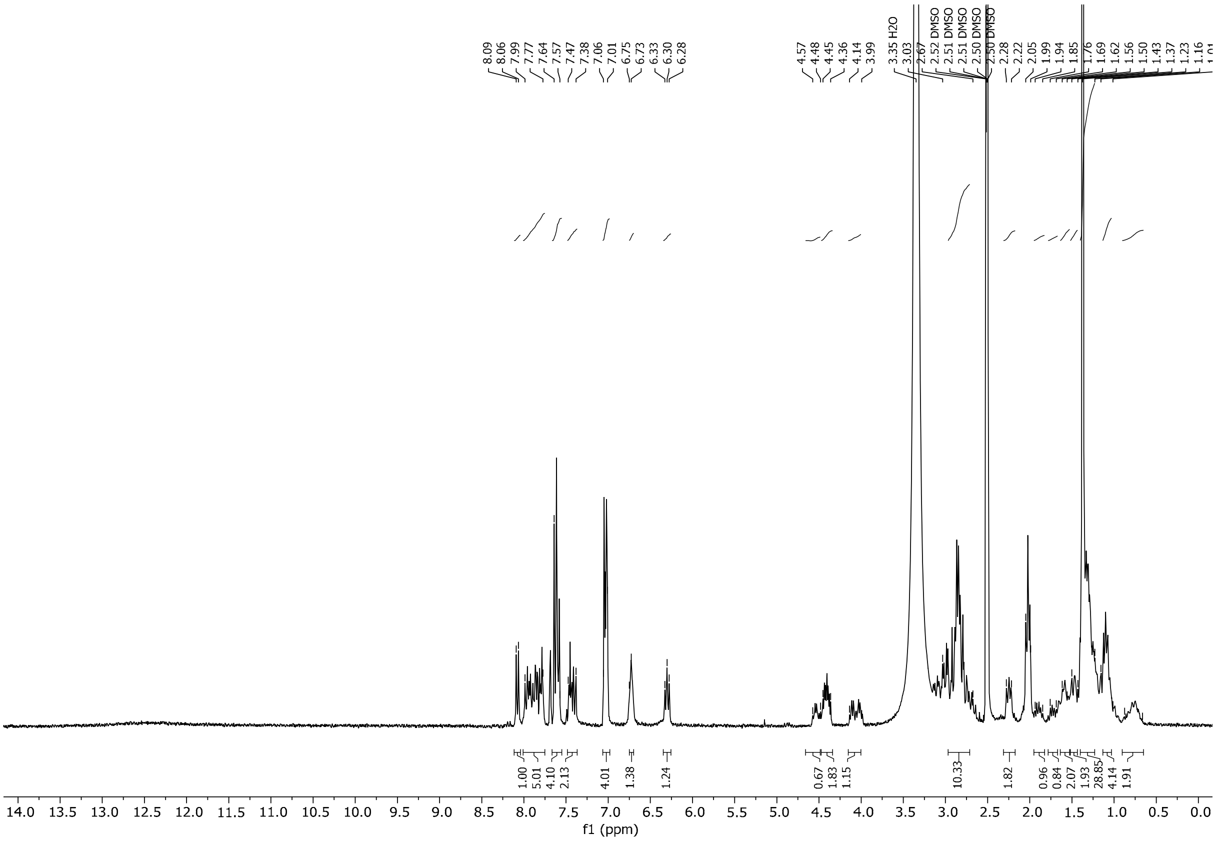


**Boc-Naphtylalanin (II)**

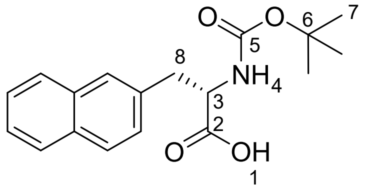

(*S*)-3-(2-Naphthyl)-alanine **I** (0.80 g, 3.7 mmol, 1.0 equiv.) was dissolved in a mixture of dioxane/ $H_2O$ (2:1) and cooled to 0°C. An aqueous solution of 1M NaOH (3.7 mL) was added followed by $Boc_2O$ (0.89 mg, 4.1 mmol, 1.1 equiv.). The mixture was stirred for 3 h at room temperature. The mixture was concentrated under reduced pressure and the basic aqueous residue was washed with EtOAc (50 mL). The aqueous phase was acidified to pH 1 with 1M HCl and washed with EtOAc (3 x 50 mL). The combined organic phases were dried over $Na_2SO_4$ and the solvent was removed under reduced pressure. The product was obtained as a colorless solid (1.1 g, 3.6 mmol, 97%).

**$^1$H NMR** (500 MHz, Methanol-*d*$_4$): δ [ppm] = 7.71 – 7.67 (m, 3H, H-ar), 7.59 – 7.55 (m, 1H, H-ar), 7.36 – 7.27 (m, 3H, H-ar), 4.36 (dd, *J* = 9.2, 5.1 Hz, 1H, H-3), 3.27 – 3.22 (m, 1H, Ha-8), 2.97 (dd, *J* = 13.9, 9.1 Hz, 1H, Hb-8), 1.22 (s, 9H, H-7).

**$^{13}$C NMR** (126 MHz, MeOD) *δ* [ppm] = 171.0 (C-5), 150.2 (C-2), 128.6 (C-ar), 128.2 (C-ar), 128.1 (C- ar), 127.2 (C-ar), 122.9 (C-ar), 122.3 (C-ar), 122.1 (C-ar), 120.8 (C-ar), 120.4 (C-ar), 75.0 (C-6), 50.8 (C-3), 32.6 (C-8), 22.9 (C-7).

**HRMS (ESI):** *m*/*z* calculated for $C_{18}H_{21}NO_4$: 338.1363, found: 338.1350 $[M+Na]^+$.

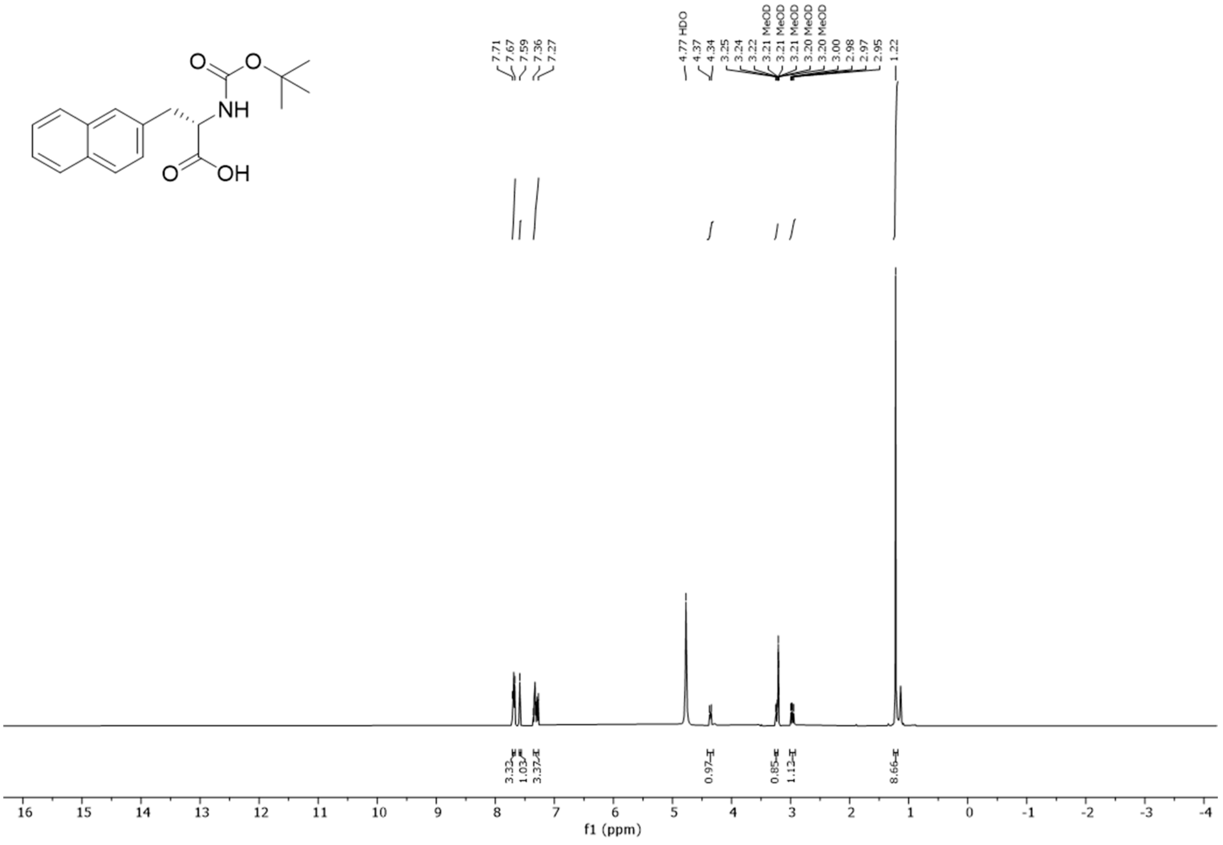

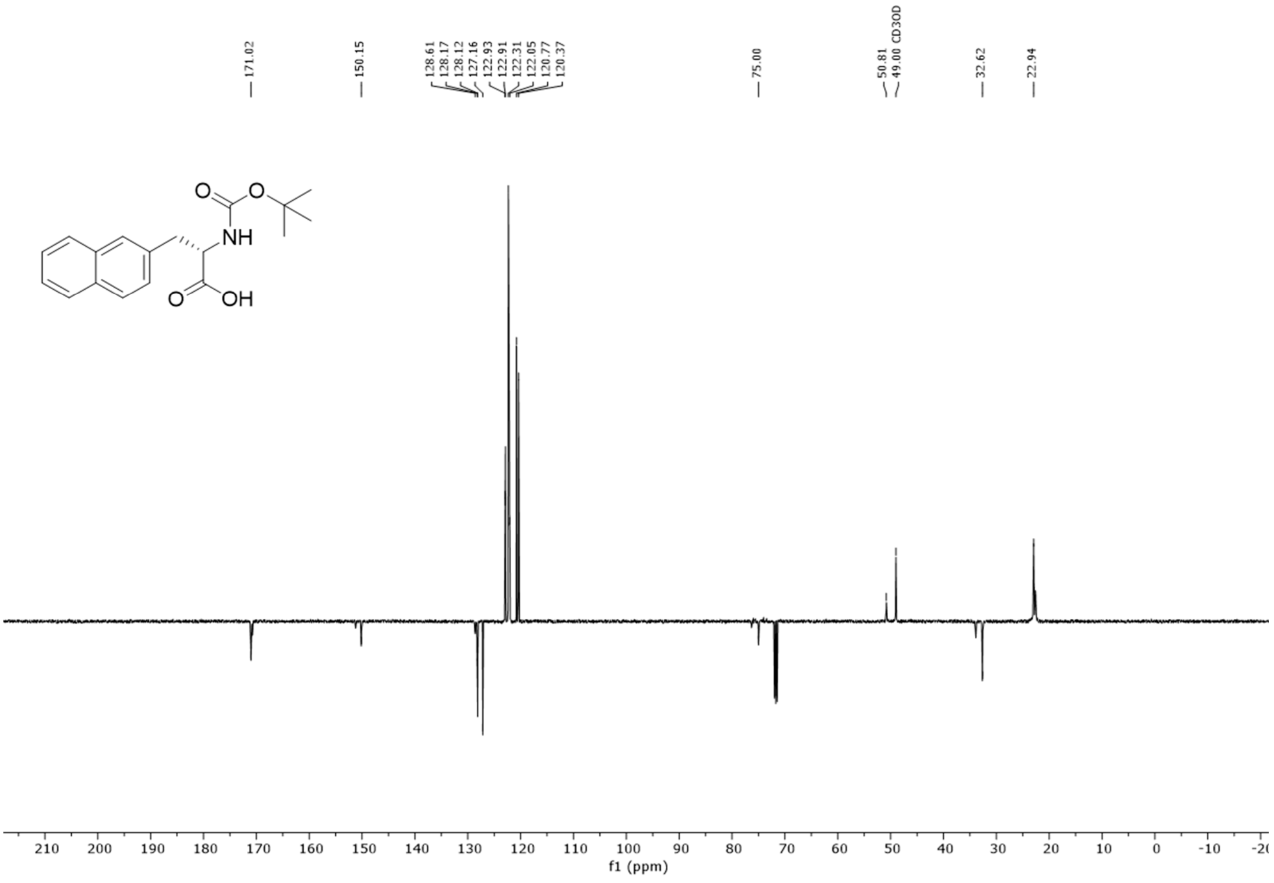


**Boc-Naphtylalanin-AE (III)**

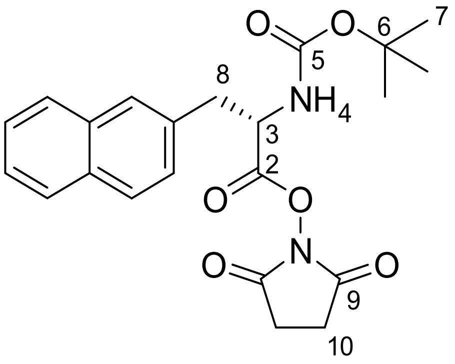

**II** Boc-naphthyl (1.10 g, 3.60 mmol, 1 equiv.) was dissolved with *N*-hydroxysuccinimde (0.61 g, 5.30 mmol, 1.5 equiv.) in dry $CH_2Cl_2$. *N*-(3-dimethylaminopropyl)-*N'*-ethyl-carbodiimide hydrochloride (EDC) (1.0 g, 5.30 mmol, 1.5 equiv.) was added to the solution and stirred under a nitrogen atmosphere overnight at room temperature. The mixture was washed with 1M HCl (20 mL) and with 1M $NaHCO_3$-Lsg (2 x 20 mL). The combined organic phases were dried over $Na_2SO_4$ and the solvent was then removed under reduced pressure. The product was obtained as a white powder (1.50 g, 3.60 mmol, 96%).

**$^1$H NMR** (500 MHz, Chloroform-*d*) δ 7.84 – 7.76 (m, 4H, H-ar), 7.49 – 7.41 (m, 3H, H-ar), 5.08 – 5.01 (m, 1H, H-3), 4.95 (d, $J$ = 8.5 Hz, 1H, H-4), 3.48 (dd, $J$ = 14.1, 6.0 Hz, 1H, Ha-8), 3.38 (dd, $J$ = 14.1, 6.1 Hz, 1H, Hb-8), 2.87 – 2.80 (m, 4H, H-10), 1.39 (s, 9H, H-7).
**$^{13}$C NMR** (126 MHz, $CDCl_3$) δ 168.8 (C-9), 167.9 (C-5), 154.8 (C-2), 133.6 (C-ar), 132.8 (C-ar), 132.3 (C-ar), 128.8 (C-ar), 128.5 (C-ar), 127.9 (C-ar), 127.8 (C-ar), 127.7 (C-ar), 126.3 (C-ar), 126.0 (C-ar), 80.8 (C-6), 52.4 (C-3), 38.3 (C-8), 28.3 (C-7), 25.7 (C-10).
**HRMS** (ESI) *m/z* calculated for $C_{22}H_{24}N_2O_6Na$: 435.1526, found: 435.1525 $[M+Na]^+$.
**DC:** $R_f$ = 0.44 ($CH_2Cl_2$/MeOH 5:1, v/v, ninhydrin).

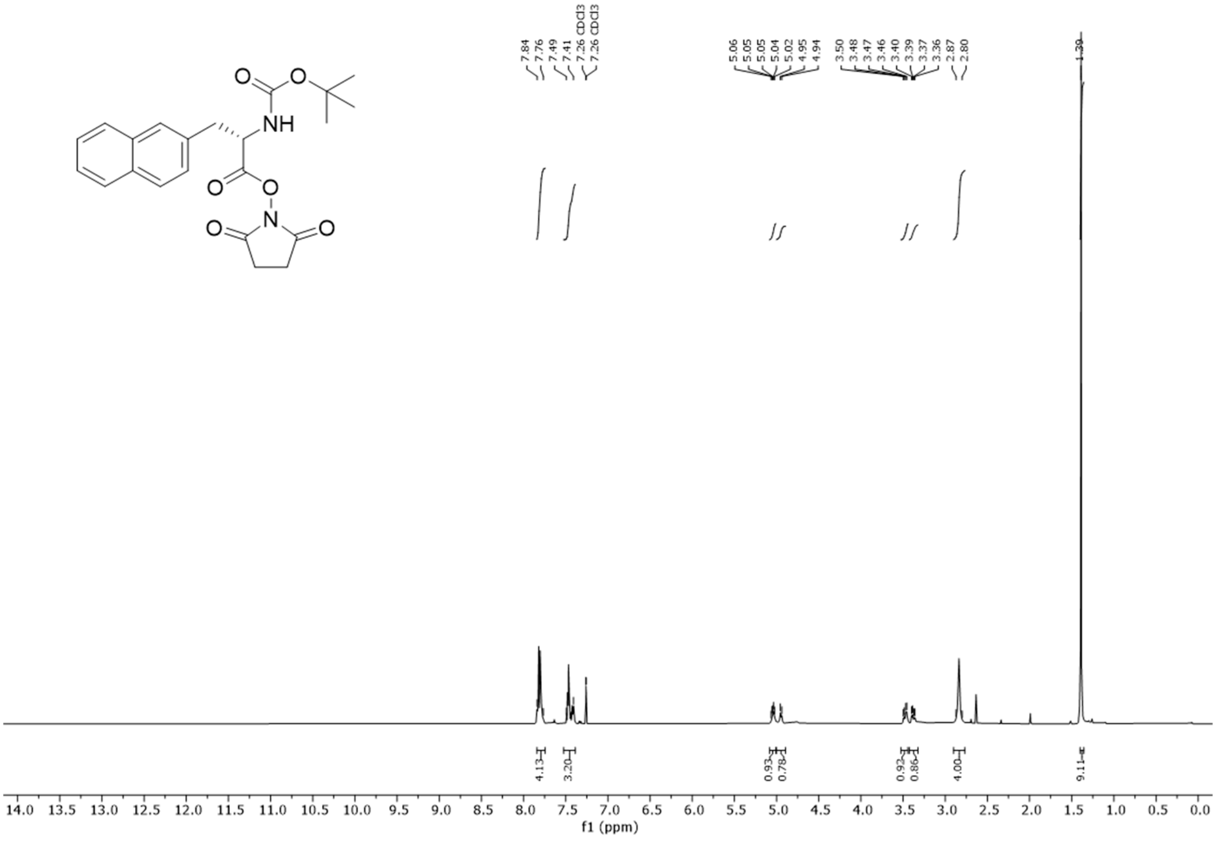

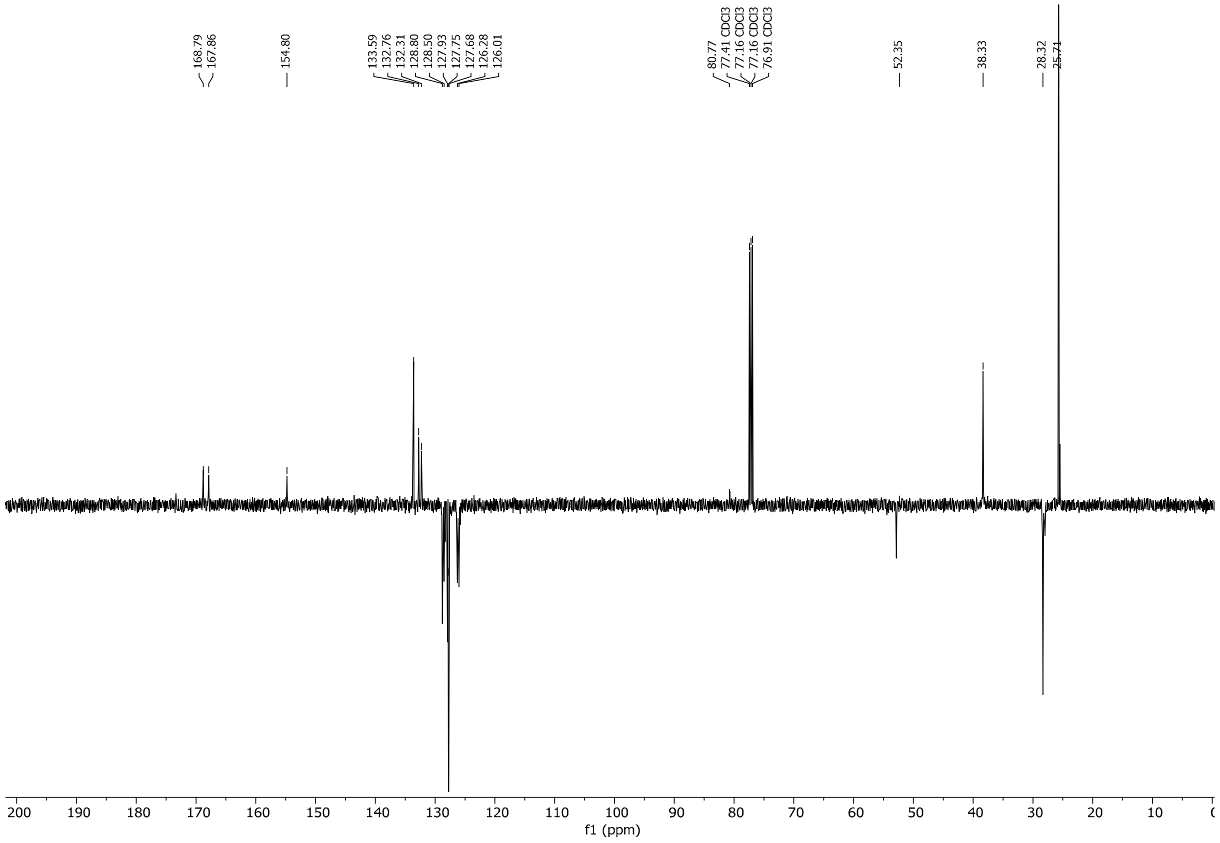


**Boc-Tranexamic acid (V)**

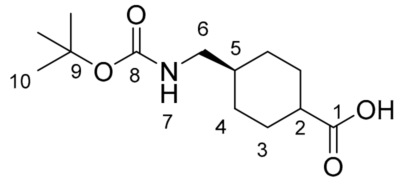


Tranexamic acid **IV** (0.80 g, 5.10 mmol, 1 equiv.) was suspended in dioxane/$H_2O$ (2:1) and cooled to 0°C. An aqueous solution of 1M NaOH (5.1 mL) was added followed by $Boc_2O$ (1.2 g, 5.60 mmol, 1.1 equiv.). The mixture was stirred for 18 h at room temperature. The mixture was concentrated under reduced pressure and the basic aqueous residue was washed with EtOAc (50 mL). The aqueous phase was adjusted to pH 1 with 1M HCl and washed with EtOAc (3 x 50 mL). The combined organic phases were dried over $Na_2SO_4$ and the solvent was removed under reduced pressure. The product was obtained as a colorless solid (1.20 g, 4.70 mmol, 94%).

**$^1$H NMR** (400 MHz, $CD_3OD$): δ 2.89 (d, *J* = 6.7 Hz, 2H, H-6), 2.24 – 2.16 (m, 1H, H-5), 2.02 – 1.97 (m, 2H, H-4), 1.84 – 1.79 (m, 2H, H-3), 1.43 (s, 9H, H-10), 1.41 – 1.33 (m, 3H, H-2, H-4), 1.02 – 0.91 (m, 2H, H-3).

**$^{13}$C NMR** (101 MHz, $CDCl_3$) δ 182.5 (C-1), 161.0 (C-8), 82.4 (C-9), 50.0 (C-6), 47.1 (C-5), 41.7 (C-2), 33.4 (C-3), 32.4 (C-10), 31.3 (C-4).

**HRMS** (ESI) *m/z* calculated for $C_{13}H_{23}NO_4$: 280.1519, found: 280.1511 $[M+Na]^+$.

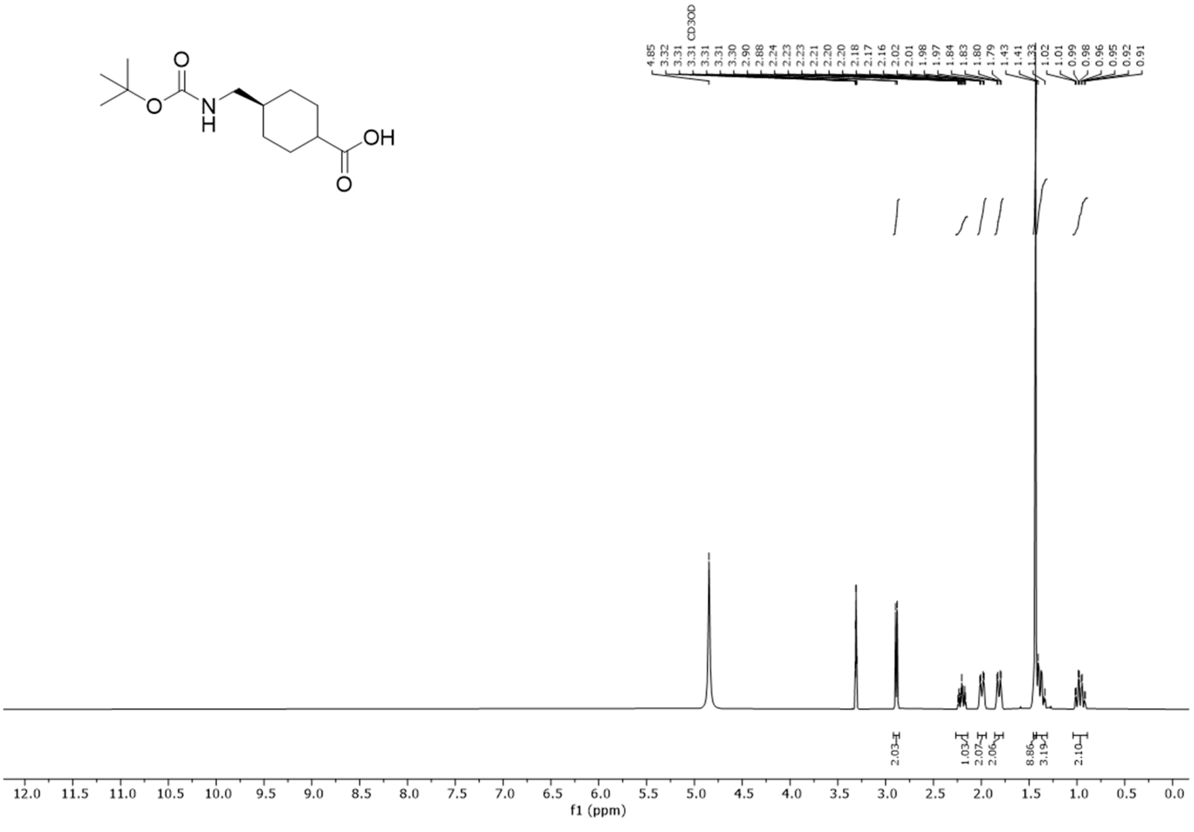

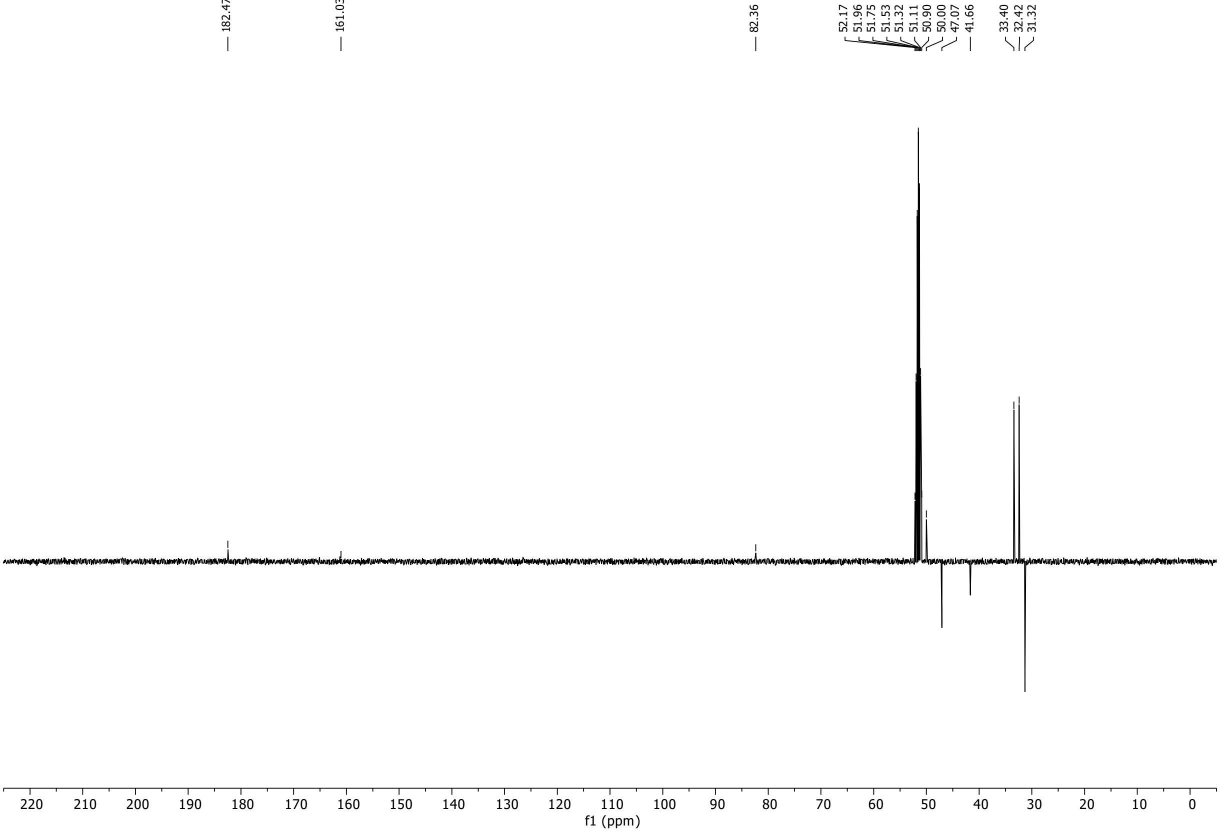

**Boc-Tranexamic acid-NHS ester (VI)**

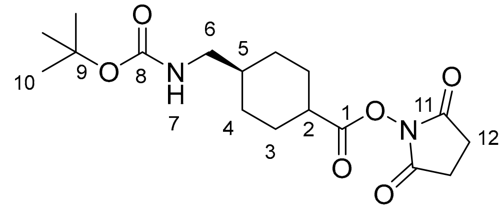


**V** (172 mg, 0.67 mmol, 1 equiv.) was dissolved in 10 mL of dry $CH_2Cl_2$ and *N*-(3-dimethylaminopropyl)-*N'*-ethyl-carbodiimide hydrochloride (EDC) (154 mg, 0.80 mmol, 1.2 equiv.) and *N*-hydroxysuccinimd (92 mg, 0.8 mmol, 1.2 equiv.) were added. The mixture was stirred under nitrogen atmosphere for 20 h at room temperature and then washed with water (3 x 2 mL). The combined organic phases were dried over $Na_2SO_4$ and the solvent was removed under reduced pressure. The product was obtained as a white powder (158 mg, 0.45 mmol, 66%).

**$^1$H NMR** (400 MHz, $CDCl_3$) : δ 4.59 – 4.51 (m, 1H, H-7), 2.93 (t, $^3J$ = 6.5 Hz, 2H, H-6), 2.76 (s, 4H, H-12), 2.55 – 2.47 (m, 1H, H-5), 2.13 – 2.08 (m, 2H, Ha-4, Ha-4), 1.81 – 1.78 (m, 2H, Ha-3, Ha-3), 1.55 – 1.44 (m, 2H, Hb-4, Hb-4), 1.39 – 1.35 (m, 10H, H-2, H-10), 1.00 – 0.90 (m, 2H, Hb-3, Hb-3).

**$^{13}$C NMR** (101 MHz, $CDCl_3$) δ 170.7 (C-1), 169.2 (C-11), 156.1 (C-8), 79.2 (C-9), 46.4 (C-6), 40.6 (C-5), 37.5 (C-2), 29.3 (C-3), 28.4 (C-10), 28.2 (C-4), 25.6 (C-12).

**HRMS** (ESI) *m/z* calculated for $C_{17}H_{26}N_2O_6$: 377.1683, found: 377.1684 $[M+Na]^+$.

**TLC:** $R_f$ = 0.58 ($CH_2Cl_2$/MeOH 20:1, v/v, ninhydrin).

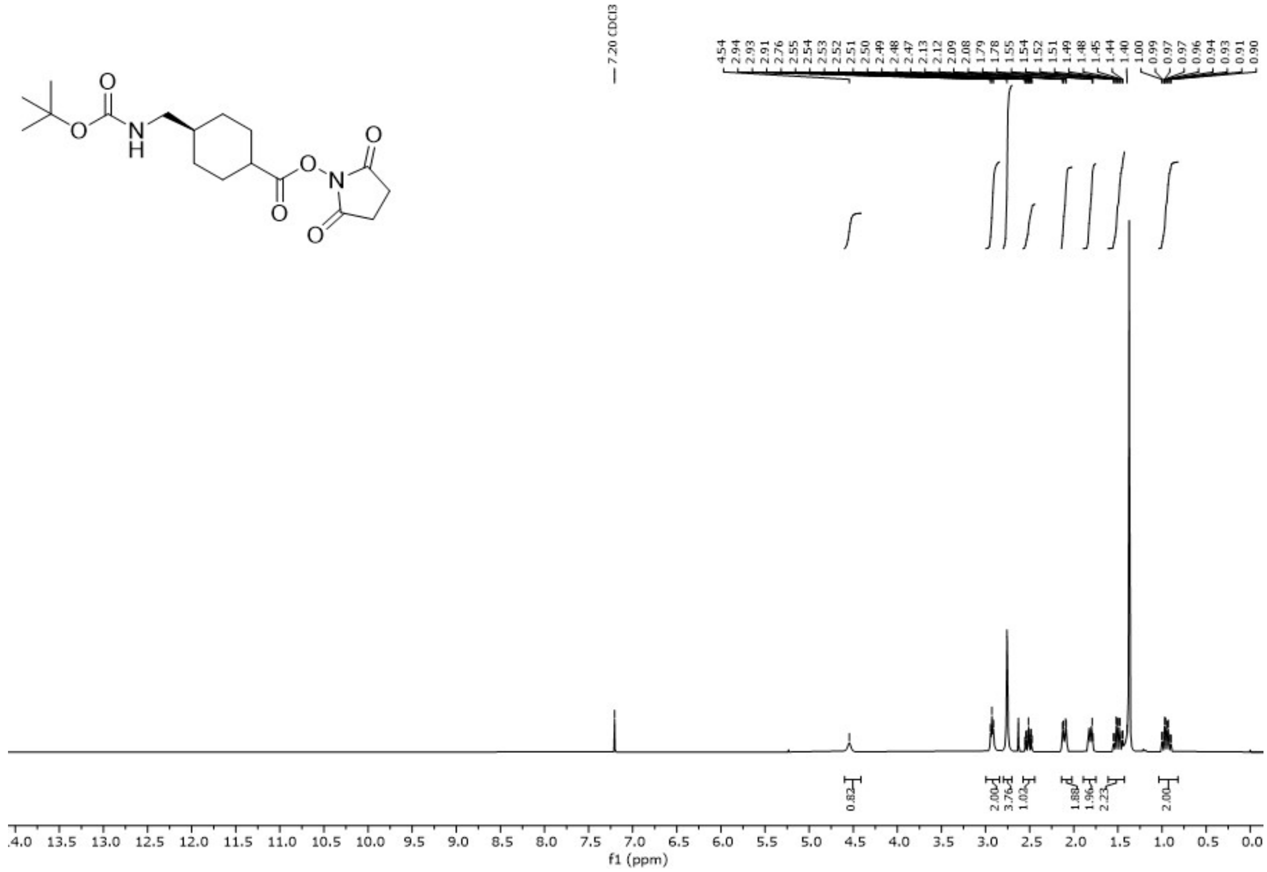

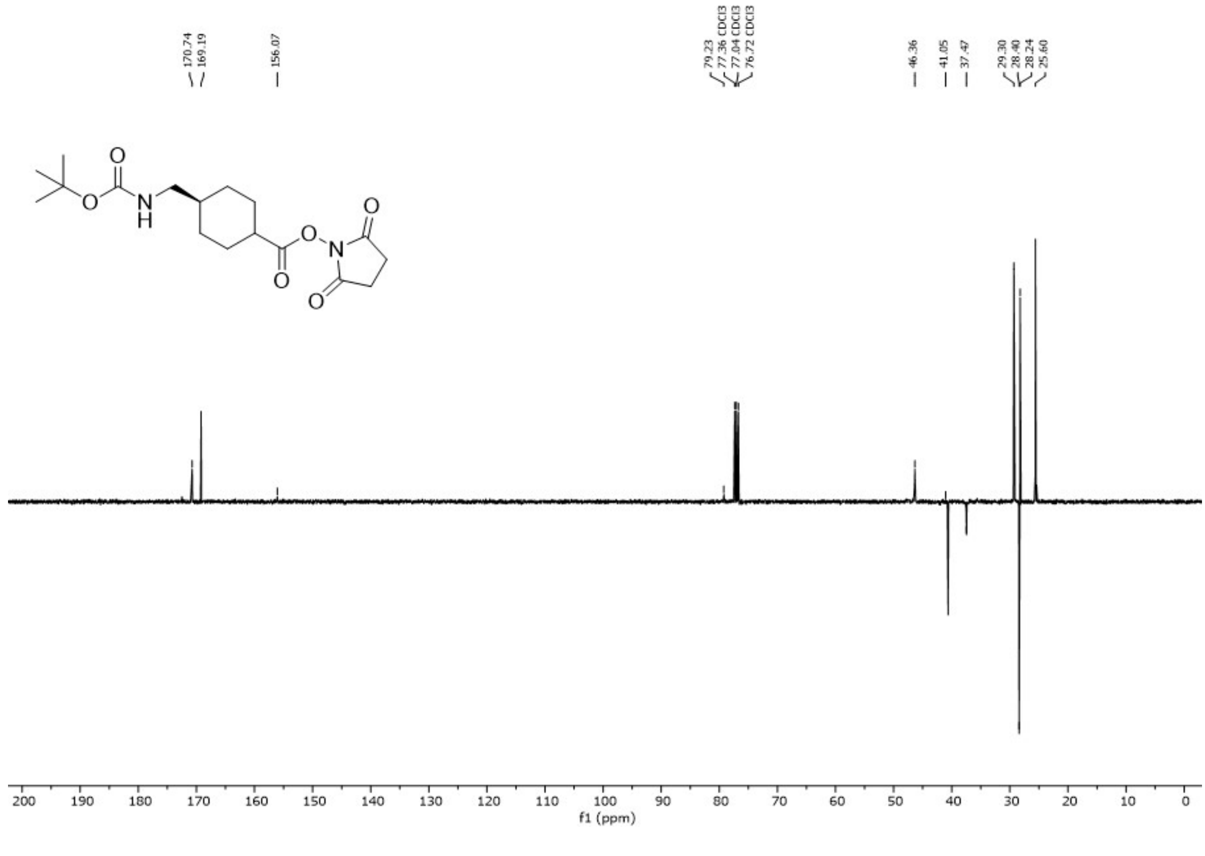

**Boc-AHX-NHS (VII)**

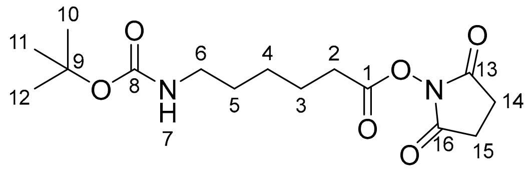

*N*-Boc-6-aminohexanoic acid (synthesized as described in the literature)[4] (2.07 g, 8.96 mmol, 1 equiv.) was dissolved with *N*-hydroxysuccinimd (1.55 g, 13.4 mmol, 1.5 equiv.) in dry $CH_2Cl_2$. *N*-(3-dimethylaminopropyl)-*N*'-ethyl-carbodiimide hydrochloride (EDC) (2.58 g, 13.4 mmol, 1.5 equiv.) was added to the solution and stirred under nitrogen atmosphere overnight at room temperature. The mixture was washed with 1M HCl (10 mL) and with 1M $NaHCO_3$-Lsg (2 x 10 mL). The combined organic phases were dried over $Na_2SO_4$ and the solvent was then removed under reduced pressure. The mixture was purified on silica gel (pentane/EtOAc 4:1 - 1:1). And the product was obtained as a white powder (1.23 g, 3.75 mmol, 42%).

**$^1$H NMR** (500 MHz, $CDCl_3$) $\delta$ [ppm] = 4.56 – 4.50 (m, 1H, H-7), 3.08 – 3.04 (m, 2H, H-6), 2.78 – 2.77 (m, 4H, H-14, H-15), 2.55 (t, $^3J$ = 7.4 Hz, 2H, H-2), 1.73 – 1.67 (p, $J$ = 7.4 Hz, 2H, H-3), 1.48 – 1.43 (m, 2H, H-5), 1.39 – 1.35 (m, 11H, H-4, H-10, H-11, H-12).
**$^{13}$C NMR** (101 MHz, $CDCl_3$) $\delta$ [ppm] = 169.2 (C1), 168.5 (C13, C16), 156.0 (C8), 79.1 (C9), 40.3 (C6), 30.9 (C2), 29.5 (C5), 28.4 (C10, C11, C12), 25.9 (C4), 25.6 (C14, C15), 24.2 (C3).
**HRMS** (ESI) *m/z* calculated for $C_{15}H_{24}N_2O_6$: 351.1526, found: 351.1523 $[M+Na]^+$.

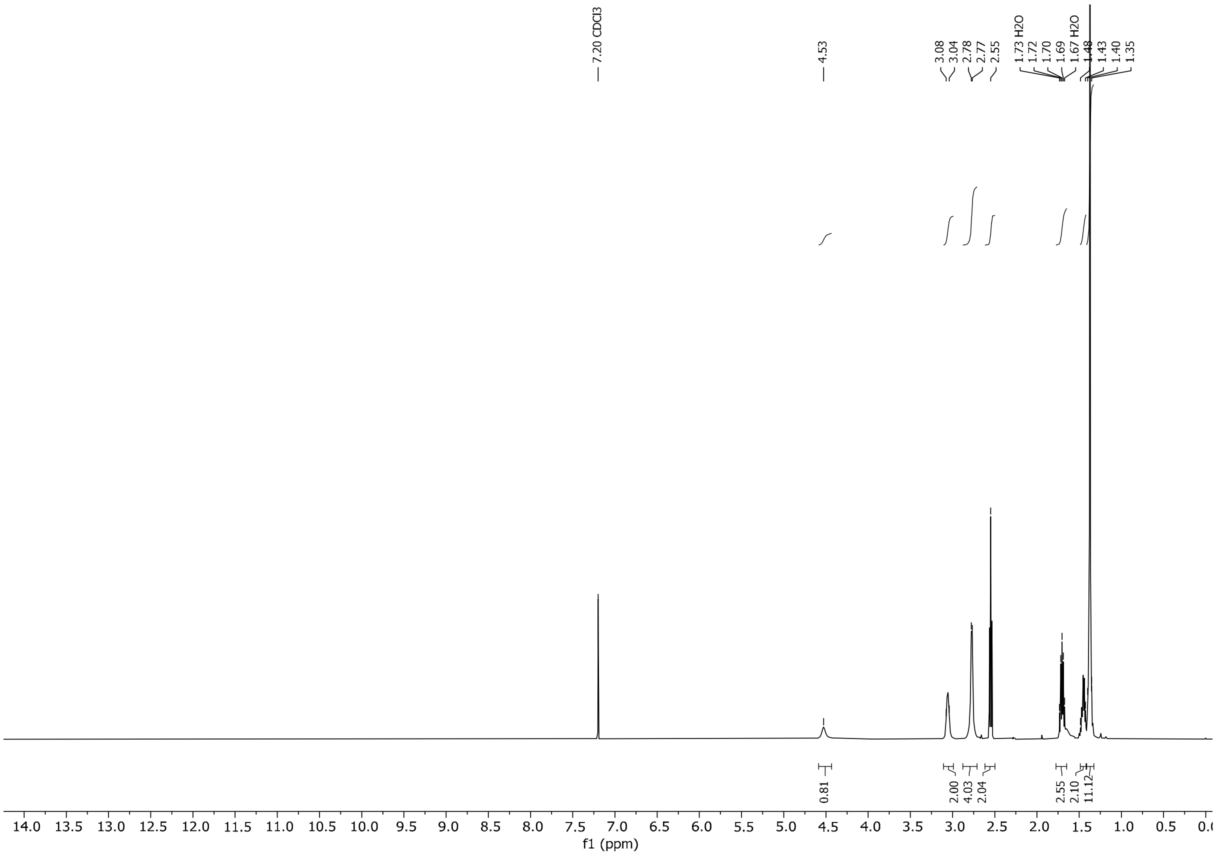
7.20 CDCl3
4.53
3.08
3.04
2.78
2.77
2.55
1.73 H2O
1.72
1.70
1.69
1.67 H2O
1.48
1.43
1.40
1.35
0.81
2.00
4.03
2.04
2.55
2.10
11.12
14.0 13.5 13.0 12.5 12.0 11.5 11.0 10.5 10.0 9.5 9.0 8.5 8.0 7.5 7.0 6.5 6.0 5.5 5.0 4.5 4.0 3.5 3.0 2.5 2.0 1.5 1.0 0.5
f1 (ppm)

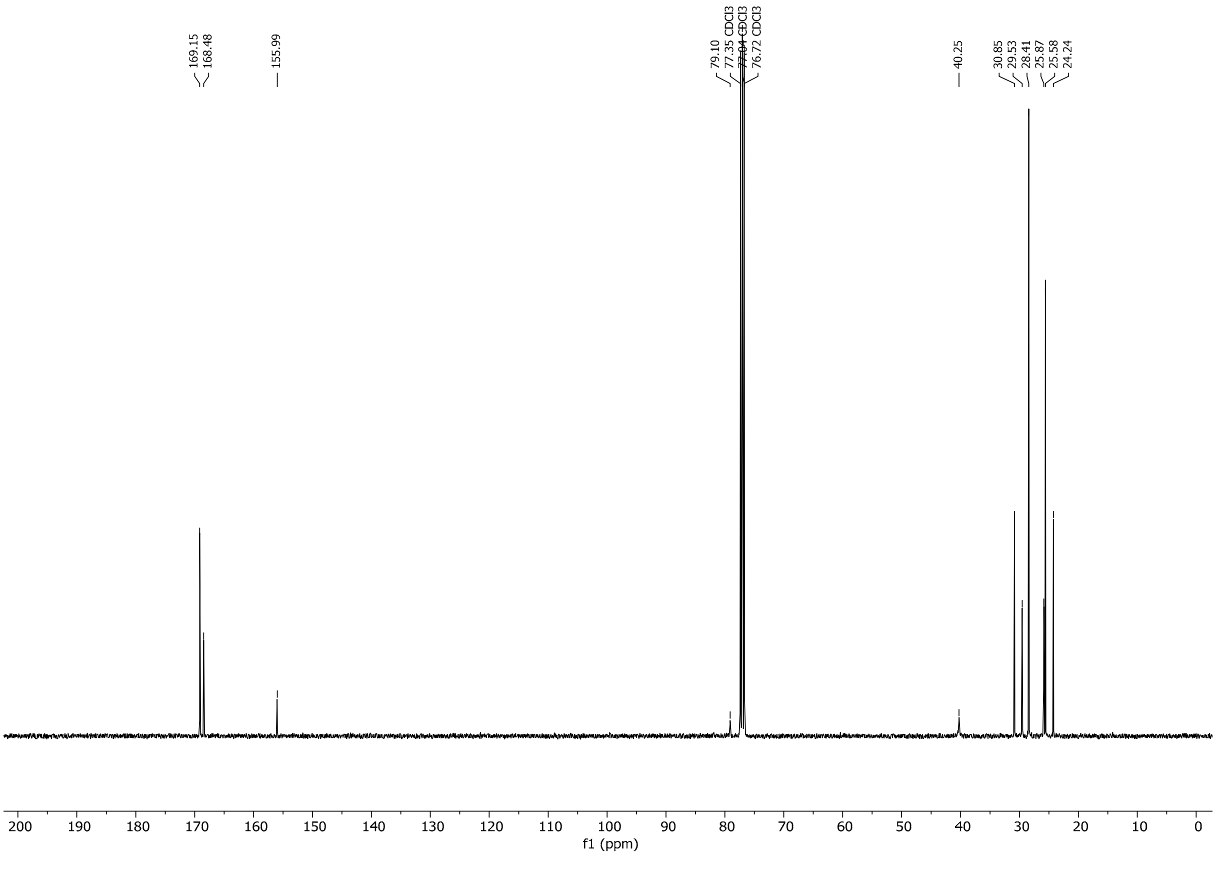
169.15
168.48
155.99
79.10
77.35 CDCl3
77.04 CDCl3
76.72 CDCl3
40.25
30.85
29.53
28.41
25.87
25.58
24.24
200 190 180 170 160 150 140 130 120 110 100 90 80 70 60 50 40 30 20 10 0
f1 (ppm)

**Boc-AHX-IPA (VIII)**

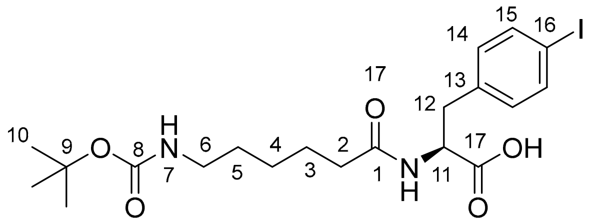

4-Iodo-L-phenylalanine (700 mg, 2.40 mmol, 1.0 equiv.) was dissolved in 10 mL DMF. After the addition of DIPEA (4.19 mL, 24.1 mmol, 10 equiv.), **VII** (947 mg, 2.90 mmol, 1.2 equiv.) was added. The reaction solution was stirred for 16 h. After removal of the solvent *in vacuo*, the residue was dissolved in $CH_2Cl_2$/MeOH and purified on silica gel ($CH_2Cl_2$/MeOH/TEA 10:1:0.2). The product was obtained as a white powder (991 mg, 2.00 mmol, 82%).

**$^1$H NMR** (400 MHz, MeOD): $\delta$ [ppm] = 7.64 (d, $^3J$ = 8.3 Hz, 2H, H-15), 7.04 (d, $^3J$ = 8.3 Hz, 2H, H-14), 4.69 (dd, $^3J$ = 9.5, 4.9 Hz, 1H, H-11), 3.20 (dd, $^3J$ = 13.9, 4.9 Hz, 1H, Ha-12), 3.01 (t, $^3J$ = 7.1 Hz, 2H, H-6), 2.91 (dd, $J$ = 13.9, 9.5 Hz, 1H, Hb-12), 2.17 (t, $^3J$ = 7.4 Hz, 2H, H-2), 1.57 – 1.40 (m, 13H, H-5, H-3, H-10), 1.26 – 1.19 (m, 2H, H-4).
**$^{13}$C NMR** (101 MHz, MeOD): $\delta$ [ppm] = 173.8 (C-1), 172.7 (C-17), 172.4 (C-8), 136.5 (C-15), 136.4 (C-13), 130.6 (C-14), 90.5 (C-16), 77.7 (C-9), 52.4 (C-11), 39.1 (C-6), 35.8 (C-12), 34.5 (C-2), 28.5 (C-3), 26.7 (C-10), 25.1 (C-4), 24.4 (C-5).
**HRMS** (ESI) *m/z* calculated for $C_{20}H_{29}IN_2O_5$: 527.1013, found: 527.1010 $[M+Na]^+$.
**TLC:** $R_f$ = 0.35 ($CH_2Cl_2$/MeOH 10:1, v/v, ninhydrin).

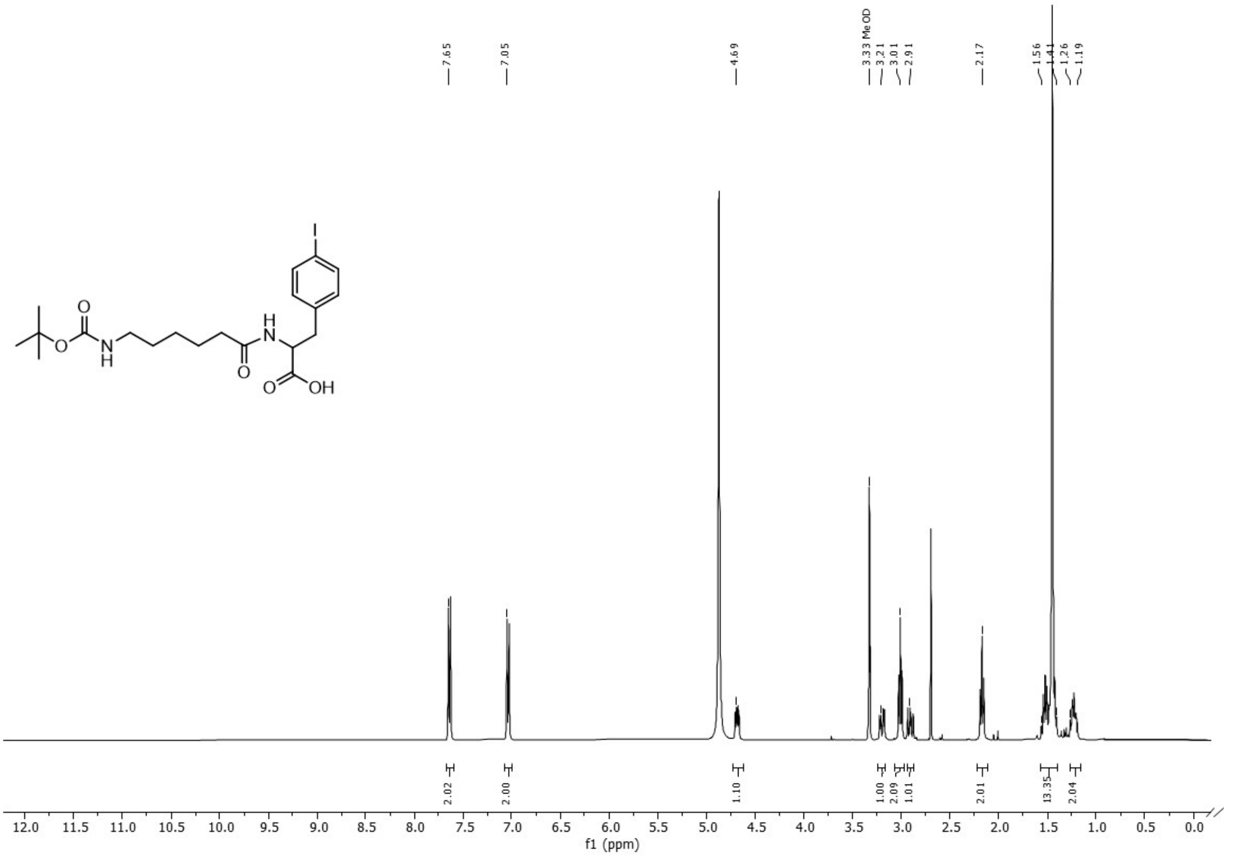
7.65
7.05
4.69
3.33 MeOD
3.21
3.01
2.91
2.17
1.56
1.41
1.26
1.19
2.02
2.00
1.10
1.00
2.09
1.01
2.01
13.35
2.04
12.0 11.5 11.0 10.5 10.0 9.5 9.0 8.5 8.0 7.5 7.0 6.5 6.0 5.5 5.0 4.5 4.0 3.5 3.0 2.5 2.0 1.5 1.0 0.5 0.0
f1 (ppm)

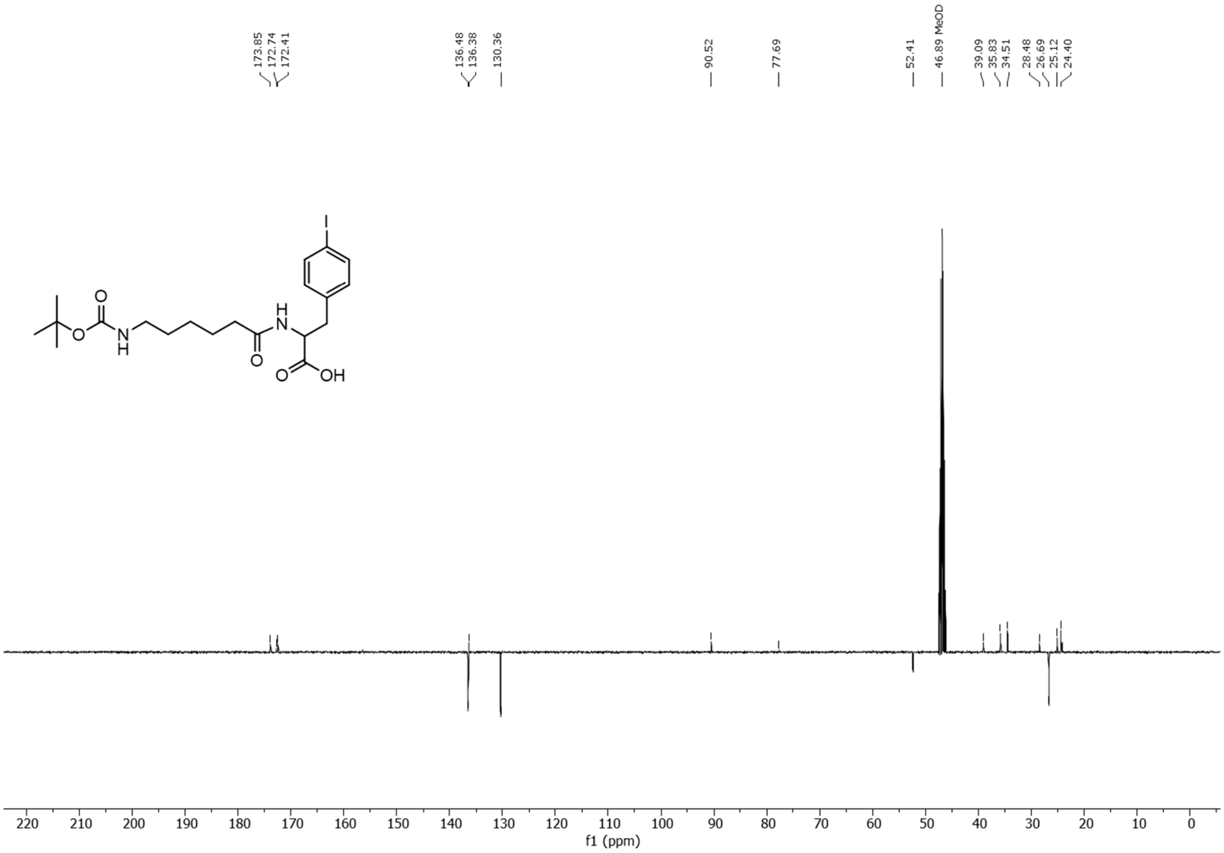
173.85
172.74
172.41
136.48
136.38
130.36
90.52
77.69
52.41
46.89 MeOD
39.09
35.83
34.51
28.48
26.69
25.12
24.40
220 210 200 190 180 170 160 150 140 130 120 110 100 90 80 70 60 50 40 30 20 10 0
f1 (ppm)

## Docking studies

Two protein structures 4mcp and C2D6 from the PDB database were used for the experiments described below. Maestro (12.7) was used as software. Docking scores are used as a mathematical value to evaluate the interaction between ligands and PSMA. A particularly good interaction is characterized by a large negative value. For the evaluation of the interactions, not only the docking score was considered, but also the position in the active center was evaluated, as well as interactions with essential amino acids and ions. PSMA-617 (without its DOTA residue) was used a reference compound.

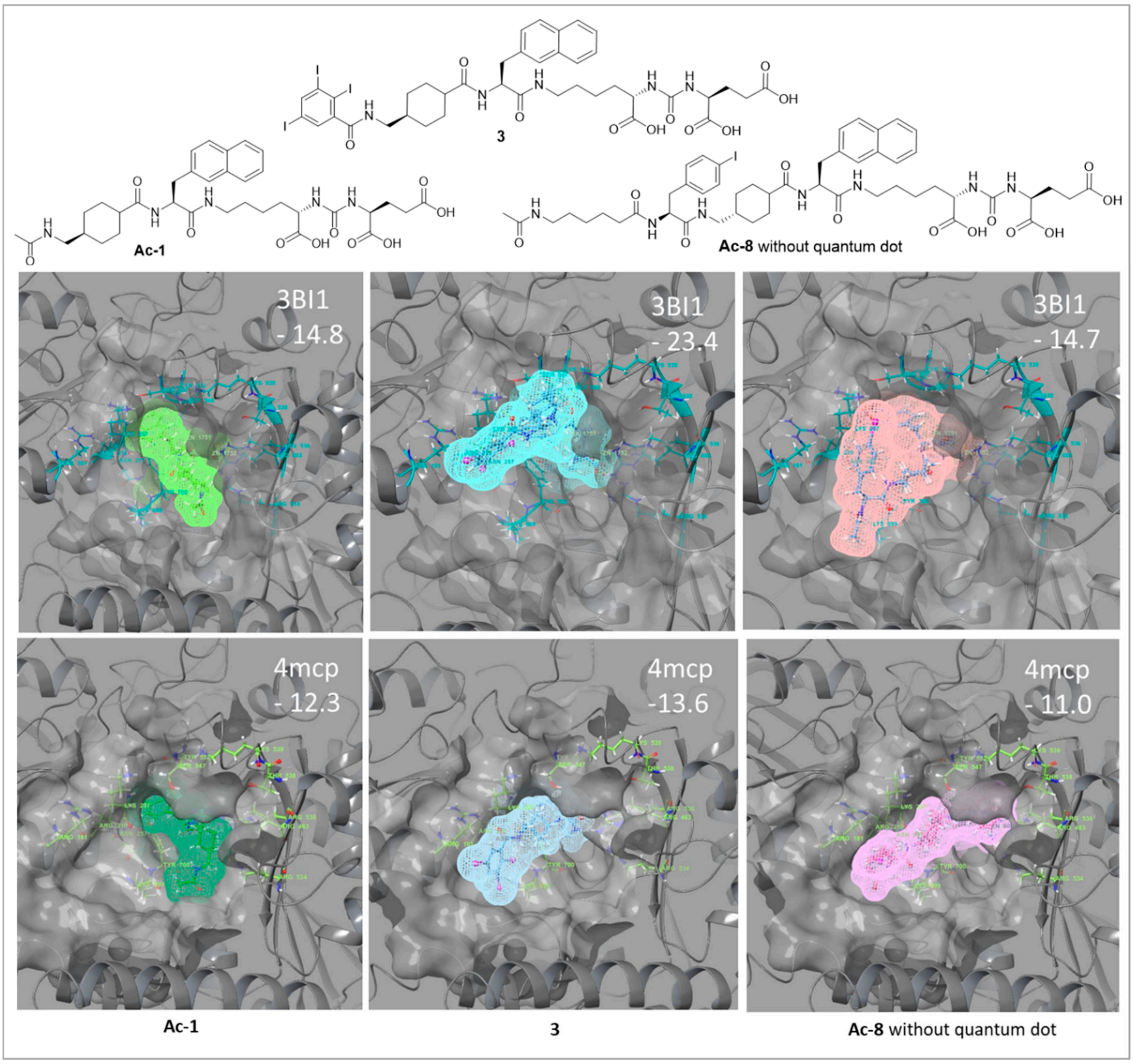


Figure S2. Chemical structures of docked compounds, docking poses and docking scores for 3BI1 (upper panel) and 4mcp as protein structures.

## References


(1) Holzapfel, M.; Mutas, M.; Chandralingam, S.; von Salisch, C.; Peric, N.; Segelke, T.; Fischer, M.; Chakraborty, I.; Parak, W. J.; Frangioni, J. V.; et al. Nonradioactive Cell Assay for the Evaluation of Modular Prostate-Specific Membrane Antigen Targeting Ligands via Inductively Coupled Plasma Mass Spectrometry. *J Med Chem* **2019**, *62* (23), 10912-10918. DOI: 10.1021/acs.jmedchem.9b01606.

(2) Kerpa, S.; Schulze, V. R.; Holzapfel, M.; Cvancar, L.; Fischer, M.; Maison, W. Decoration of 1,4,7,10-tetraazacyclododecane-1,4,7,10-tetraacetic acid (DOTA) with N-oxides increases the T(1) relaxivity of Gd-complexes. *ChemistryOpen* **2024**, e202300298. DOI: 10.1002/open.202300298.
(3) Mixdorf, J. C.; Hoffman, S. L. V.; Aluicio-Sarduy, E.; Barnhart, T. E.; Engle, J. W.; Ellison, P. A. Copper-Mediated Radiobromination of (Hetero)Aryl Boronic Pinacol Esters. *J Org Chem* **2023**, *88* (4), 2089-2094. DOI: 10.1021/acs.joc.2c02420.
(4) Kim, J. H.; Ali, K. H.; Oh, Y. J.; Seo, Y. H. Design, synthesis, and biological evaluation of histone deacetylase inhibitor with novel salicylamide zinc binding group. *Medicine (Baltimore)* **2022**, *101* (17), e29049. DOI: 10.1097/MD.0000000000029049.
